# The Dual Role of Connexins in Stroke, Neurotrauma, Neurodegenerative and Psychiatric Disorders: A Global Systematic Review

**DOI:** 10.3390/molecules31081341

**Published:** 2026-04-19

**Authors:** Stanislav Rodkin, Mitkhat Gasanov, Alexander Tushev, Elena Belousova, Yulia Gordeeva, Chizaram Nwosu, Anastasia Tolmacheva

**Affiliations:** 1 Research Laboratory “Medical Digital Images Based on the Basic Model”, Department of Bioengineering, Institute of Living Systems, Don State Technical University, 344000 Rostov-on-Don, Russia; 2 Hospital Therapy Department, Medical Institute, Yaroslav-the-Wise Novgorod State University, 173003 Veliky Novgorod, Russia; 3 Neurology and Neurosurgery Department, Rostov State Medical University, 344022 Rostov-on-Don, Russia; 4 Hospital Therapy Department, Kazan State Medical University, 420012 Kazan, Russia; 5 Faculty Therapy Department Named after Professor G.D. Zalessky, Novosibirsk State Medical University, 630091 Novosibirsk, Russia; 6 Medical Rehabilitation Department, Novosibirsk Regional Clinical Hospital of War Veterans No. 3, 630005 Novosibirsk, Russia

**Keywords:** connexin, hemichannels, gap junctions, astrocytes, neurons, stroke, traumatic brain injury, spinal cord injury, peripheral nerve injury, neurodegenerative diseases, Alzheimer’s disease, Parkinson’s disease, amyotrophic lateral sclerosis, Huntington’s disease, psychiatric disorders, depression, bipolar disorder, schizophrenia, epilepsy

## Abstract

**Background:** Connexins (Cx) are a family of transmembrane proteins that form gap junctions and connexin hemichannels (HCs), enabling direct intercellular communication within the nervous system. Connexin 43 (Cx43), the principal astrocytic connexin, exhibits a context-dependent dual role: under physiological conditions it maintains tissue homeostasis and metabolic support, whereas under pathological conditions excessive activation of Cx43 hemichannels promotes neuroinflammation, excitotoxicity, blood–brain barrier disruption, and secondary neural tissue damage. Other connexin isoforms also contribute to the pathogenesis of neurological and psychiatric disorders through alterations in neuronal synchronization, glial signaling, and myelin integrity. **Objective:** To systematize current evidence on the role of key connexin isoforms in acute nervous system injuries—including stroke, traumatic brain injury, spinal cord injury, and peripheral nerve injury—as well as chronic disorders such as neurodegenerative diseases, epilepsy, and psychiatric disorders, with particular emphasis on the functional duality of connexin channels and the therapeutic potential of their selective modulation. **Methods:** A systematic literature search was conducted in the PubMed, Scopus, and Web of Science databases in accordance with the PRISMA framework and the PRISMA Extension for Scoping Reviews guidelines. The review included data from experimental models, postmortem brain studies, genetic association analyses, and pharmacological intervention studies. The retrieved studies were screened, assessed for eligibility, and integrated using a qualitative narrative synthesis approach. **Results:** In acute neural injuries, hyperactivation of Cx43 hemichannels amplifies inflammatory signaling, edema formation, and neuronal death, whereas selective HCs inhibitors reduce lesion volume and improve functional outcomes in experimental models. Connexin 36 (Cx36) contributes to cortical spreading depolarization and seizure propagation, while Connexin 32 (Cx32) and Connexin 47 (Cx47) are critically involved in oligodendrocyte function and white-matter demyelination. In PNI, Cx43 upregulation contributes to neuropathic pain, whereas mutations in Cx32 cause hereditary demyelinating neuropathies. In neurodegenerative diseases—including Alzheimer’s disease, Parkinson’s disease, and amyotrophic lateral sclerosis—Cx43 hemichannel activity promotes neuroinflammation and pathological protein accumulation, while reduced Cx32/Cx47 expression disrupts metabolic support of axons. In psychiatric disorders such as major depressive disorder, bipolar disorder, and schizophrenia, decreased astrocytic connexin expression (Cx43 and Cx30) has been associated with impaired glial–neuronal communication and cognitive–emotional dysfunction. In epilepsy, increased Cx43/Cx30 expression contributes to neuronal hypersynchronization and blood–brain barrier dysfunction, whereas selective hemichannel blockade suppresses seizure activity. **Conclusions:** Cx—particularly Cx43—occupies a central position in the molecular mechanisms of secondary neural injury and network dysfunction. The dual functional properties of gap junctions and hemichannels determine their context-dependent effects across neurological and psychiatric diseases. Selective inhibition of pathological HCs activity shows significant neuroprotective and anticonvulsant potential and represents a promising direction for the development of targeted therapeutic strategies. Further studies are required to determine optimal therapeutic time windows, tissue-specific effects, and the long-term safety of Cx modulation.

## 1. Introduction

**Figure 1:**
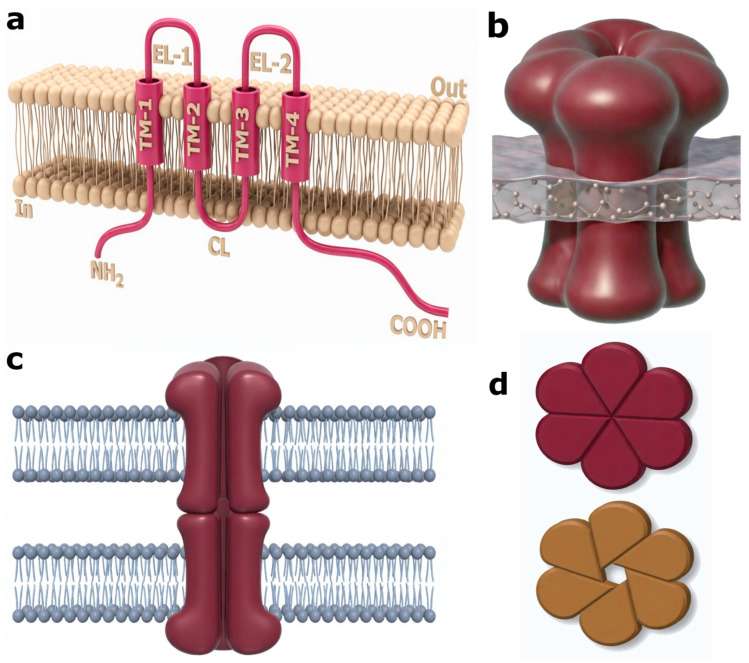
Structure and organization of Cx and gap junctions. (**a**) Schematic representation of Cx topology: a transmembrane protein with four hydrophobic domains (TM1–TM4), two extracellular loops, one intracellular loop, and cytoplasmic N- and C-terminal regions. (**b**) Structure of a hexameric connexon: six Cx molecules assemble symmetrically to form a ring-shaped hollow structure—a hemichannel. (**c**) Gap junction: two hexameric connexons located in the membranes of adjacent cells align precisely with each other to form a continuous intercellular channel enabling direct exchange of ions and small molecules. (**d**) Comparison of closed and open hemichannel states: illustration of conformational rearrangements that regulate channel permeability and control functional opening and closure.

Connexins (Cx) are a family of transmembrane proteins that form gap junctions (GJs) and HCs and serve as key mediators of intercellular communication in the nervous system. Each connexin exhibits a conserved topology consisting of four α-helical transmembrane domains (TM1–TM4), two extracellular loops (EL1 and EL2) containing conserved cysteine residues that form disulfide bonds enabling specific docking of connexons between adjacent cells, one intracellular loop (CL), and short N-terminal and long variable C-terminal cytoplasmic domains that serve as platforms for post-translational modifications and protein–protein interactions (Figure 1a). Six connexin subunits oligomerize into a hexameric connexon, which may function as an independent HC (Figure 1b) or dock with a connexon from an adjacent cell (Figure 1c), forming an intercellular channel with a diameter of approximately 1.2–1.8 nm that allows the exchange of molecules up to ~1–1.5 kDa. An important property of Cx channels is their ability to undergo conformational regulation: both HCs and complete GJs can transition between closed and open states under the influence of extracellular calcium, pH, membrane potential, redox status, and other factors. In the closed state, N-terminal helices (NTH) or other structural elements occlude the pore, preventing the passage of ions and small molecules; channel opening involves significant conformational rearrangements, including the displacement of NTH toward the pore wall, thereby enabling permeability (Figure 1d). Through these channels, cells exchange ions, metabolites, and signaling molecules, thereby synchronizing neuronal and glial activity and maintaining homeostasis in neural tissue. Although Cx-based gap junction channels are permeable primarily to ions and small molecules, Cx also participates in intercellular transfer of larger cellular components, including mitochondria, through indirect mechanisms such as tunneling nanotubes, extracellular vesicles, and connexin-associated membrane interactions rather than through the channel pore itself. The diversity of Cx isoforms, which are heterogeneously expressed across tissues, largely determines their unique physiological and pathological properties [1,2]. Dysregulation of these proteins is associated with a wide spectrum of disorders, including neurotrauma of various etiologies, stroke, neurodegenerative diseases, and psychiatric disorders. The diversity of Cx isoforms, their ability to form heterotypic and homotypic combinations, and their tissue- and cell-specific expression patterns collectively determine the unique functional properties of these channels under both physiological and pathological states.

The most extensively studied and functionally significant connexin in neural tissue is Cx43, the principal astrocytic Cx. During acute injury, Cx43 demonstrates pronounced functional duality: when incorporated into GJs it can support metabolic coupling, mitochondrial transfer, and regenerative processes [3,4,5], whereas hyperactivation of Cx43-HCs leads to massive release of adenosine triphosphate (ATP), glutamate, activation of pro-inflammatory cascades, excitotoxicity, brain edema, and secondary cell death [6,7,8,9,10]. Selective inhibition of Cx43 hemichannels significantly reduces lesion volume, attenuates inflammation, and improves functional deficits across multiple experimental models of acute CNS injury, including transient or permanent middle cerebral artery occlusion (MCAO), oxygen–glucose deprivation/reperfusion (OGD/R), controlled cortical impact (CCI), fluid percussion injury (FPI), and spinal cord contusion or compression models [7,8,11,12].

Other connexin isoforms also play critical roles. Cx36, predominantly expressed in neurons, regulates electrical synchronization and cortical spreading depolarization (CSD); its blockade reduces infarct size in stroke [13] and seizure activity in epilepsy [14]. Cx32, expressed in oligodendrocytes and Schwann cells, is essential for myelination and metabolic support of axons; its dysfunction underlies demyelinating neuropathies [15] and contributes to white matter pathology in stroke [16,17]. Cx30 and Cx47 participate in astrocyte–oligodendrocyte communication and maintenance of the myelin sheath; alterations in these proteins have been reported in neurodegenerative diseases and psychiatric disorders [18,19,20].

In chronic neurodegenerative diseases such as Alzheimer’s disease, Parkinson’s disease, amyotrophic lateral sclerosis, and Huntington’s disease, systemic dysregulation of connexins contributes to disease progression. Hyperactivation of Cx43 HCs enhances neuroinflammation and the accumulation of pathological proteins, reduced Cx32 expression disrupts oligodendrocyte-mediated metabolic support of motor neurons, and alterations in Cx36 and Cx30 affect synaptic plasticity and clearance of toxic protein aggregates [18,21,22,23,24]. In psychiatric disorders, decreased expression of Cx43 and Cx30 in the cortex and hippocampus correlates with disruption of the astrocytic syncytium, neuroinflammation, and cognitive–emotional deficits [25,26,27,28].

In our previous studies, we investigated the mechanisms regulating the expression of several key pro-apoptotic proteins under conditions of neuronal stress [29,30,31,32]. In addition, in a recent study, we analyzed changes in the expression and localization of Cx43 in neurons and glial cells following TBI [33].

The aim of the present review is to systematize current knowledge on the tissue-specific expression and functions of major Cx isoforms in the nervous system and to analyze their contribution to the pathogenesis of central and peripheral nervous system injuries, stroke, neurodegenerative diseases, and psychiatric disorders. Particular attention is given to the dual functional properties of HCs and GJs, potential targets for pharmacological modulation, and unresolved questions requiring further investigation.

## 2. Methods

The present review was conducted in accordance with the Preferred Reporting Items for Systematic Reviews and Meta-Analyses (PRISMA) framework and the PRISMA Extension for Scoping Reviews (PRISMA-ScR) guidelines [34]. The review protocol was not prospectively registered. The review is primarily narrative and integrative in nature, as the main emphasis was placed on qualitative analysis, interpretation, and integration of available evidence rather than on quantitative meta-analytic synthesis of results. The objective of this study was to perform a systematic search and comprehensive synthesis of current evidence regarding the role of different Cx isoforms in the pathophysiology of neurotrauma, neurodegenerative and psychiatric disorders, as well as stroke.

All stages of the study were conducted independently by the authors, including literature search, assessment of relevance and methodological quality, data extraction, and the subsequent analysis and synthesis of the collected information. The literature search primarily covered publications from 2015 to 2026, allowing the inclusion of the most recent and relevant evidence available at the time of manuscript preparation. Priority was given to recent studies, whereas earlier landmark publications were included when necessary to provide conceptual and historical context.

### 2.1. Source Search

Information sources were identified through a systematic search of the international bibliographic databases PubMed, Scopus, and Web of Science. The search was conducted without language restrictions or limitations regarding full-text availability but was restricted by publication year (2015–2026) to ensure comprehensive coverage of relevant studies while maintaining a focus on contemporary evidence. In selected cases, earlier publications were also included.

The following keywords and their combinations were used: “connexin”, “gap junction”, “hemichannel”, “Cx43”, “Cx36”, “brain injury”, “spinal cord injury”, “neurodegeneration”, “Alzheimer’s disease”, “Parkinson’s disease”, “psychiatric disorders”, “schizophrenia”, “depression”, “stroke”, “glial cells”, and “neuroinflammation”. Boolean operators (*AND*, *OR*, *NOT*), as well as synonyms and related terms, were applied to refine and expand the search strategy. The detailed parameters of the search strategy are presented in Table 1 and Table S1.

**Figure 2:**
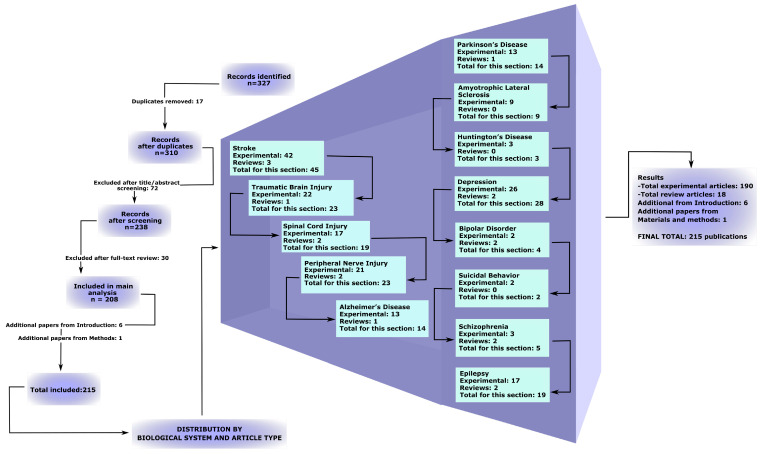
Expanded PRISMA-ScR flowchart illustrating the process of study identification, screening, eligibility assessment, and inclusion in the review, with details by publication type in the relevant sections.

The initial search in the PubMed, Web of Science, and Scopus databases identified 327 publications. After the removal of 17 duplicates, 310 records remained. During the screening of titles and abstracts, 72 studies were excluded, followed by an additional 30 exclusions after full-text assessment. Ultimately, 208 scientific articles were included in the main qualitative analysis. Additionally, 6 studies were included in the introduction but not used in subsequent sections presenting the main results, and 1 source was included in Section 2. The detailed selection process is illustrated in the PRISMA-ScR flow diagram (Figure 2, Supplementary Table S2). An expanded PRISMA-ScR flowchart providing additional details of the study selection process can be found in the Supplementary Figure S1.

### 2.2. Assessment of Study Quality

Following the initial screening, the relevance of publications was evaluated based on their titles and abstracts. Articles that met the thematic criteria underwent full-text analysis. Study quality was assessed according to the following inclusion criteria:

1. Clear and logically structured presentation of the obtained data;
2. Presence of appropriate statistical analysis of results;
3. Adequate selection of experimental models, methods, and sample size;
4. Consistency between the applied methodology and the stated research objectives;
5. Correct interpretation of the obtained results.

Publications with methodological limitations, absence of peer review, or insufficient scientific significance were excluded. The final decision regarding the inclusion of each study was made by the authors based on a comprehensive assessment of methodological quality and informational value.

### 2.3. Conceptualization and Data Synthesis

All selected articles underwent detailed analysis followed by conceptualization and systematization of the extracted information. Particular attention was paid to the expression and regulation of different Cx isoforms, their contribution to the pathogenesis of central and peripheral nervous system injuries, stroke, neurodegenerative diseases, and psychiatric disorders, as well as their potential as targets for pharmacological modulation.

The collected data were structured as a narrative synthesis complemented by tabular and graphical visualization. Data integration was performed using a thematic approach, including identification of key patterns, analysis of inconsistencies within the literature, and recognition of existing knowledge gaps. The results of this synthesis allowed the identification of major mechanisms underlying the involvement of Cx in the pathogenesis of the aforementioned disorders and highlighted promising directions for future research.

## 3. Results

### 3.1. Role of Connexins in Stroke

**Figure 3:**
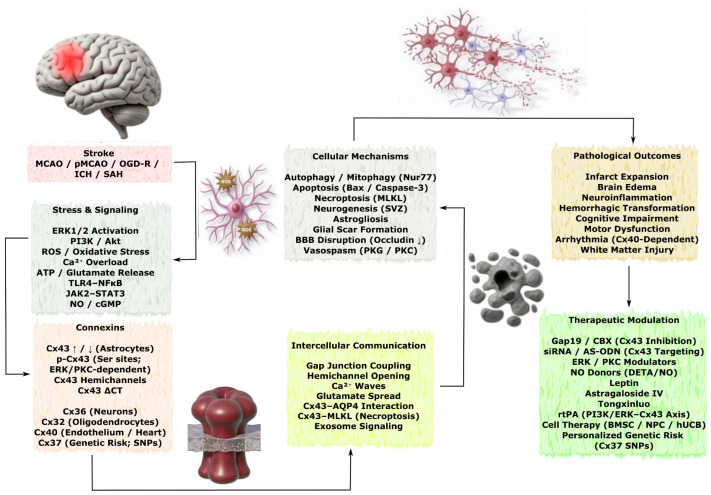
Connexin-Mediated Mechanisms in Stroke. This schematic diagram summarizes the role of connexins in the molecular and cellular mechanisms underlying ischemic and hemorrhagic stroke. Experimental models such as middle cerebral artery occlusion (MCAO), permanent MCAO (pMCAO), oxygen–glucose deprivation/re-oxygenation (OGD-R), intracerebral hemorrhage (ICH), and subarachnoid hemorrhage (SAH) induce ischemia, reperfusion injury, and hemorrhagic damage. These insults trigger oxidative stress, Ca^2+^ overload, and activation of intracellular signaling pathways, including ERK1/2, PI3K/Akt, TLR4–NFκB, JAK2–STAT3, and nitric oxide (NO)/cGMP signaling, accompanied by ATP and glutamate release. These processes regulate the expression, phosphorylation, and function of connexins in neural and vascular cells. Cx43, primarily expressed in astrocytes, undergoes dynamic changes in expression and phosphorylation at multiple serine residues through ERK- and protein kinase C (PKC)-dependent mechanisms, leading to altered GJ coupling and hemichannel activity. Additional connexins involved in stroke pathology include neuronal Cx36, oligodendrocytic Cx32, endothelial and cardiac Cx40, and Cx37, whose genetic polymorphisms have been associated with stroke susceptibility. Connexin-dependent signaling modulates intercellular communication through GJs, hemichannel opening, Ca^2+^ wave propagation, glutamate spread, and extracellular vesicle signaling, as well as protein interactions such as Cx43–AQP4 and Cx43–MLKL. These mechanisms influence multiple cellular processes, including autophagy and mitophagy, apoptosis, necroptosis, neurogenesis, astrogliosis, glial scar formation, blood–brain barrier disruption, and vascular dysfunction. Collectively, they contribute to major pathological outcomes after stroke, including infarct expansion, brain edema, neuroinflammation, hemorrhagic transformation, cognitive impairment, motor dysfunction, arrhythmia, and white matter injury. Therapeutic strategies targeting connexins or related signaling pathways—such as Gap19, carbenoxolone (CBX), siRNA or antisense oligodeoxynucleotides (AS-ODN), ERK/PKC modulators, nitric oxide donors, natural compounds, reperfusion therapy with rtPA, and cell-based therapies—may modulate these processes and promote neuroprotection and functional recovery. Arrows indicate direction of change: ↑ increased expression/activity; ↓ decreased expression/activity. Abbreviations: MCAO, middle cerebral artery occlusion; pMCAO, permanent MCAO; OGD-R, oxygen–glucose deprivation/re-oxygenation; ICH, intracerebral hemorrhage; SAH, subarachnoid hemorrhage; ROS, reactive oxygen species; ERK1/2, extracellular signal-regulated kinase 1/2; PI3K, phosphoinositide 3-kinase; PKC, protein kinase C; NO, nitric oxide; BBB, blood–brain barrier; AQP4, aquaporin-4; MLKL, mixed lineage kinase domain-like protein; SVZ, subventricular zone; CBX, carbenoxolone; AS-ODN, antisense oligodeoxynucleotide; siRNA, small interfering RNA; rtPA, recombinant tissue plasminogen activator; SNP, single nucleotide polymorphism.

Stroke is one of the leading cardiovascular diseases and represents an acute disruption of cerebral blood circulation that triggers a cascade of molecular and cellular reactions and initiates a broad spectrum of neurodegenerative processes. These alterations lead to neuronal and glial cell death through interconnected mechanisms in which neuroinflammation, excitotoxicity, oxidative stress, and mitochondrial dysfunction promote activation of apoptotic pathways and disruption of neuroglial communication [35]. The key signaling pathways, Cx-dependent mechanisms of intercellular interaction, and major pathological consequences of stroke are schematically illustrated in Figure 3.

Given the marked heterogeneity of stroke subtypes and their temporal progression, this section first examines ischemic stroke (primarily modeled by middle cerebral artery occlusion—MCAO, permanent MCAO—pMCAO, and global ischemia) and then hemorrhagic stroke (intracerebral hemorrhage—ICH and subarachnoid hemorrhage—SAH). Special attention is paid to the temporal evolution of Cx changes across the hyperacute/acute, subacute, and chronic phases, as the functional consequences of connexin modulation differ substantially depending on the timing and stroke subtype. Most of the data discussed below derive from focal ischemic stroke models (primarily transient or permanent MCAO), while findings from global ischemia, ICH and SAH are presented separately where relevant.

Cxs, including Cx43, Cx36, Cx40, Cx32, and Cx37, represent fundamental molecular components in the pathogenesis of stroke, regulating signaling processes in astrocytes, neurons, endothelial cells, and other elements of the NVU. Their expression forms a dynamic regulatory system that rapidly responds to physiological and pathological changes. Studies using both in vivo and in vitro models highlight the ambivalent biological effects of Cx proteins: depending on their expression levels, phosphorylation status, and interactions with molecular targets, they may initiate either neuroprotective or cytotoxic responses, ultimately modulating cell death, neuroinflammation, and BBB integrity [36].

#### 3.1.1. Dual Role of Cx43 in Ischemic Stroke

Cx43 plays a dual role in the pathogenesis of stroke. This duality is particularly evident in ischemic stroke models, most commonly represented by transient or permanent MCAO, where Cx43 exhibits distinct effects during the acute and chronic phases of injury. Under physiological conditions, Cx43 forms GJs that enable the exchange of ions, metabolites, and signaling molecules between astrocytes, neurons, and oligodendrocytes. This function is essential for maintaining central nervous system (CNS) homeostasis and neuroglial communication. However, experimental studies have demonstrated that during ischemic stroke, modeled by MCAO or OGD/R, both the expression level and functional profile of Cx43 change significantly. For example, an enriched environment (EE) that promotes neuroplasticity and reduces infarct volume, oxidative stress, and neuroinflammation in rats subjected to MCAO exerts its protective effects through inhibition of Cx43 expression and suppression of the TLR4/MyD88/NF-κB signaling pathway in the ischemic penumbra, demonstrating a Cx43-dependent neurotoxic effect during the acute phase of stroke [6]. Similar results were obtained using the selective inhibitor of Cx43-associated hemichannels, the mimetic peptide Gap19. In MCAO models, this inhibitor reduced infarct volume, neuronal apoptotic death, and inflammation through phosphorylation of JAK2/STAT3 [8], as well as by decreasing the expression of Toll-like receptor 4 (TLR4), tumor necrosis factor-α (TNF-α), and inflammatory cytokines [9]. Importantly, it is primarily Cx43 hemichannels, rather than GJs, that are most frequently associated with neurotoxicity during stroke. Their hyperactivation triggers extensive pathomorphological, biochemical, and molecular-genetic cellular changes leading to neuronal and glial cell death [8,9].

Nevertheless, Cx43 can also mediate neuroprotective signaling that promotes regeneration of damaged neural tissue after stroke. Under OGD/R conditions, reduced Cx43 expression is accompanied by translocation of the protein from the cell membrane to the cytoplasm, astrocytic retraction, increased intracellular Ca^2+^ levels, and activation of the CaMKII/CREB pathway, resulting in increased expression of Ephrin-A4 and impaired astrocytic function. Conversely, substantial upregulation of Cx43 expression under these pathological conditions stabilizes astrocytes and promotes regenerative processes in cortical neurons, facilitating neurite outgrowth [3].

In chronic ischemia models associated with multiple strokes resulting from cancer and pneumonia, increased Cx43 immunoreactivity in the penumbra has been interpreted as a neuroprotective astrocytic response aimed at enhancing intercellular neuroglial communication under hypoxic conditions. In contrast, in acute embolic stroke models, activation of Cx43 was associated with neuronal death [37].

#### 3.1.2. Cx43 in Hemorrhagic Stroke

In models of ICH induced by collagenase injection or hemin stimulation, Gap19 reduced Cx43 expression via the ubiquitin–proteasome pathway, enhanced nuclear translocation of Yes-associated protein (YAP), and increased the expression of anti-inflammatory genes SOCS1 and SOCS3, thereby reducing hematoma volume, reactive astrogliosis, and motor and cognitive impairments [7].

#### 3.1.3. Temporal Dynamics and Phosphorylation of Cx43

The complex role of Cx43 is reflected in its dynamic spatiotemporal expression during stroke. In the acute phase of permanent MCAO (pMCAO), increased Cx43 levels in the central ischemic region have been detected 2–3 h after pMCAO and are accompanied by astrocyte activation and a progressive inflammatory response. However, by 6 h, Cx43 levels decrease sharply, likely due to massive cell death and protein degradation. Cx43 expression correlates with the neuronal damage marker Fluoro-Jade C, detectable as early as 1 h after pMCAO, while decreased levels of microtubule-associated protein 2 (MAP2) and reduced thionin staining further confirm ongoing neurodegeneration [38]. In rats with perinatal hypoxic–ischemic (HI) brain injury, a pronounced inflammatory response associated with microglial activation and reactive astrogliosis was observed, mediated by upregulation of Cx43 expression that gradually declined during transition to the chronic phase [39]. However, mice with heterozygous Cx43 knockout (Cx43+/−) demonstrate increased infarct volume, enhanced apoptotic signaling, and reduced astrogliosis four days after MCAO [40,41]. Similarly, astrocyte-specific Cx43 knockout (Cre+) is associated with adverse molecular and cellular outcomes in stroke, including increased infarct volume, neuroinflammation, and apoptosis [42].

Cx43-mediated GJs play an important role in neuroprotection during ischemia by maintaining intracellular homeostasis within the pathophysiological microenvironment induced by stroke. Under physiological conditions, GJs maintain the neuroglial syncytium and participate in the transport of metabolites, neurotransmitters, and ions. During ischemia, preserved GJs may contribute to both neuroprotective and neurotoxic processes [2]. Mice with a truncated C-terminal domain of Cx43 (Cx43ΔCT) exhibit increased brain injury, reduced astrogliosis, and enhanced infiltration of inflammatory cells into the peri-infarct region. The C-terminal domain is likely essential for maintaining channel conductance, hemichannel activity, and propagation of Ca^2+^ waves, making it a critical structural element in protective mechanisms during cerebral ischemia [43]. Phosphorylation of Cx43 at serine residues near the C-terminus represents a key regulatory mechanism controlling its functional properties. In pMCAO models, mice carrying a Cx43 phosphorylation-deficient mutation (MK4)—which prevents phosphorylation at PKC/CK1/MAPK sites—display enhanced neuroprotective responses compared with wild-type animals, including reduced infarct volume, increased astrocyte reactivity, decreased microglial activation, and improved cognitive outcomes [44]. Bilateral carotid artery occlusion increased phosphorylated Cx43 levels through activation of the ERK pathway, leading to the formation of heteromeric Cx43/Cx40 complexes that contribute to brain injury. Inhibition of ERK or application of siRNA produced neuroprotective effects and stabilized the BBB [45].

#### 3.1.4. Cx43 Interactions with AQP4 and Blood–Brain Barrier Integrity

Several studies indicate that Cx43, through close interaction with aquaporin-4 (AQP4), may mediate neuroprotective mechanisms. In MCAO models, co-expression of Cx43 and AQP4 in the subventricular zone (SVZ) and peri-infarct cortex correlated with enhanced neurogenesis. This effect was absent in mice lacking Cx43 and AQP4 or when Cx43 was inhibited by the mimetic peptide CMP. These groups also exhibited elevated levels of inflammatory markers IL-1β and TNF-α in the peri-infarct cortex and SVZ compared with wild-type animals [46]. However, another study showed that the p38MAPK inhibitor SKF-86002 reduced brain edema, apoptosis, oxidative stress, inflammation, and BBB permeability by decreasing expression of Cx43 and AQP4 in MCAO models. Co-immunoprecipitation experiments demonstrated a physical interaction between Cx43 and AQP4 in astrocytes [47]. Transgenic mice lacking Cx43 expression or its C-terminal domain exhibit numerous microhemorrhages and increased BBB permeability associated with altered density and mobility of Cx43 and AQP4 plaques in perivascular astrocytic endfeet [48]. Additional studies have reported decreased Cx43 levels in isolated brain capillaries accompanied by increased AQP4 expression [49].

#### 3.1.5. Role of Other Connexins in Stroke Pathogenesis

Other connexins also contribute significantly to stroke pathogenesis. Although Cx43 remains the most extensively studied isoform, other connexins play important roles depending on the stroke subtype and phase. For instance, neuronal Cx36 participates in cortical spreading depolarization (CSD), a detrimental phenomenon that exacerbates neuronal damage following cerebral ischemia. In Cx36-deficient mice, both the number and duration of CSD events were markedly reduced, resulting in smaller infarct size and improved motor recovery after ischemia, primarily in models of focal cerebral ischemia [13]. Pharmacological blockade of neuronal GJs with mefloquine or genetic deletion of Cx36 prevents neuronal death in models of photothrombotic ischemia and NMDA-induced neurodegeneration, without affecting NMDA receptor expression or activity [50]. In the hippocampus following global ischemia, expression of Cx36 and Cx32 increases in parvalbumin-positive GABAergic interneurons, contributing to their survival and resistance to ischemic stress. Regulation of these Cx proteins occurs mainly at translational or post-translational levels, as mRNA expression remains unchanged. Knockout of Cx32 leads to increased vulnerability to ischemia, suggesting a neuroprotective signaling role mediated by Cx32-associated GJs [16]. In rat organotypic hippocampal cultures, Cx36-expressing interneurons demonstrate resistance to OGD by maintaining electrical coupling through GJs, as confirmed by colocalization of Cx36 with glutamate decarboxylase 67 (GAD67) and α7 nicotinic acetylcholine receptors (α7-nAChR) [51].

Cx32, expressed in oligodendrocytes and interneurons, may contribute to neuronal injury through negative regulation of autophagy, a critical protective mechanism during cerebral ischemia. Experimental studies using OGD/R and MCAO models demonstrate increased Cx32 expression, whereas its inhibition enhances autophagy through activation of the nuclear receptor Nur77. Upon activation, Nur77 translocates to mitochondria and initiates mitophagy, ultimately attenuating neuronal injury [17]. In studies of ischemic white matter injury induced by bilateral carotid artery stenosis (BCAS), a model of chronic cerebral hypoperfusion, decreased expression of Cx32 and Cx30 correlated with impaired oligodendrogenesis and cognitive deficits. Disruption of astrocyte–oligodendrocyte GJs was also observed, as indicated by altered colocalization of Cx30 and Cx32 under BCAS conditions [52].

Cx40, predominantly expressed in endothelial cells and the sinoatrial node, is associated with post-stroke cardiac complications. In pMCAO models, decreased Cx40 expression in the sinoatrial node correlated with cardiac arrhythmias. Genetic modification of cardiac cells using the viral vector rAAV9-Gja5, which increases Cx40 expression, improved cardiac conduction and reduced arrhythmias [53]. Cx40 also acts as a negative regulator of cerebral vasospasm (CVS) via activation of the NO/cGMP/PKG signaling pathway following SAH. Administration of the nitric oxide donor DETA/NO or the PKG activator 8-Br-cGMP increased Cx40 expression and induced vascular relaxation under ischemic conditions. Conversely, PKG inhibition (KT5823) or blockade of Cx40 (40Gap27) produced opposite effects [54]. In SAH models, Cx43 has also been implicated in cerebral vasospasm through enhanced GJ-mediated intercellular communication and activation of the PKC signaling pathway. Application of the PKC inhibitor chelerythrine and Cx43 siRNA induced vascular constriction, whereas oxyhemoglobin (OxyHb) increased GJ activity and Cx43 expression in vitro [55]. Increased heteromeric Cx43/Cx45 GJs after SAH are also associated with cerebral vasospasm, whereas inhibition of this Cx interaction promotes vasodilation [56].

Genetic association studies in ischemic stroke patients have also linked genetic predisposition to Cx37. In the Chinese Han population, SNPs rs1764390 and rs1764391 in the CONNEXIN37 gene are associated with increased stroke risk, particularly under additive and dominant genetic models. The AG and GG genotypes of rs1764390 and the CC genotype of rs1764391 increase susceptibility to stroke, while the interaction between rs1764391 and smoking further elevates risk, highlighting the role of Cx37 in inflammatory and atherosclerotic processes contributing to stroke [57,58]. Moreover, interaction between rs1764391 and rs918592 of the PDE4D gene further increases stroke risk, indicating complex genetic interactions [58].

#### 3.1.6. Therapeutic, Regenerative and Additional Aspects of Connexin Modulation

Cx proteins are important molecular regulators of neuroinflammation and apoptotic signaling during stroke. One of the central mechanisms driving these processes is the Cx43–atypical chemokine receptor 3 (ACKR3) axis in astrocytes. In SAH models, disruption of the Cx43–ACKR3 signaling pathway has been proposed as a promising therapeutic strategy for neuroprotection [59]. Gap19 administration in ICH models suppresses pro-inflammatory TLR4–NF-κB and JAK2–STAT3 pathways, reducing levels of IL-1β and TNF-α and promoting an anti-inflammatory astrocytic phenotype [7]. The neuroprotective hormone leptin, secreted by adipocytes, also effectively decreases Cx43 levels through ERK1/2 phosphorylation, reducing glutamate release and neuronal apoptotic death both in vivo and in vitro [60]. In turn, astragaloside IV, a biologically active triterpene glycoside, exerts protective effects against cerebral ischemia/reperfusion (I/R) injury by increasing expression of Cx36 and PKA, while simultaneously decreasing the Bax/Bcl-2 ratio, thereby inhibiting the mitochondrial apoptotic pathway [61]. It has also been shown that Cx36 expression is significantly increased under OGD conditions, contributing to enhanced neuronal apoptosis. This effect is associated with the activation of Ca^2+^/calmodulin-dependent protein kinase II, which enhances Cx36-mediated intercellular communication through GJs and promotes the propagation of pro-apoptotic signals between cells. Pharmacological inhibition or downregulation of Cx36 significantly increases neuronal cell viability, reducing the level of apoptosis [62].

Neuroprotective effects of Cx proteins are also mediated through their involvement in neurogenesis and regeneration. Intra-carotid administration of bone marrow stromal cells (BMSCs) increases expression of Cx43 and bone morphogenetic proteins 2/4 (BMP2/4) in the ischemic boundary zone, correlating with increased astrocyte proliferation, enhanced synaptophysin expression, and significant functional recovery after right MCAO [4]. Injection of neural progenitor cells (NPCs) in microsphere embolism models restored Cx43 levels in brain capillaries, improving astrocyte–endothelial interactions within the NVU and reducing cognitive impairment [49].

Traditional Chinese medicine also provides interesting insights. Analysis of the compound Tongxinluo (TXL) demonstrated strong neuroprotective potential in cerebral ischemia mediated through the Cx43/Calpain II/Bax/Caspase-3 signaling pathway, reducing neuronal apoptosis and increasing Cx43 expression on days 3, 7, and 14 after I/R injury. Administration of the Cx43 inhibitor CBX abolished these effects, confirming a Cx43-dependent neuroprotective mechanism [63]. Acupuncture, a non-pharmacological intervention widely used in traditional Chinese medicine, has also been shown to modulate astrocytic Cx43-dependent signaling via the ERK1/2 pathway. In models of embolic stroke with delayed rtPA thrombolysis, acupuncture reduced infarct volume, restored BBB integrity, and improved neurological outcomes. Although distinct from classical pharmacological approaches, these findings further support the concept that connexin signaling can be therapeutically modulated through diverse strategies influencing intracellular signaling cascades and glial functional states rather than direct channel blockade [64].

In addition, Cx are increasingly recognized as important mediators of exosome-mediated intercellular communication in the CNS, a rapidly expanding area of research with considerable therapeutic potential. Activation of the cAMP/PKA signaling pathway enhances the incorporation of Cx43 into exosomes, thereby facilitating their uptake by astrocytes, attenuating blood–brain barrier disruption and cerebral edema, and improving cognitive function, spatial learning, and memory following cerebral ischemia [65]. Transplantation of human umbilical cord blood cells (hUCB) in models of perinatal hypoxia–ischemia reduces reactive astrogliosis and Cx43 expression, attenuating inflammation and promoting post-ischemic plasticity, which correlates with improved motor function [39].

Formation of the glial scar, in which Cx43 participates through regulation of astrocyte proliferation, may exert both neuroprotective and cytotoxic effects. While it can confine the lesion site and limit damage spread, it may also inhibit neuronal plasticity. In focal cerebral ischemia, increased Cx43 expression and mRNA levels in reactive astrocytes within the lesion area correlate with their proliferative activity, indicating a role of Cx43 in glial tissue remodeling during scar formation [66].

The functional profile of Cx proteins is closely linked to mechanisms maintaining BBB integrity. Administration of rtPA increases phosphorylation of Cx43 through PI3K and ERK pathways, enhancing BBB permeability and the risk of hemorrhagic transformation. Inhibitors of Cx43 signaling, including LY294002 and U0126, attenuate these effects by reducing phosphorylated Cx43 expression in endothelial cells following hypoxia/re-oxygenation (H/R) [67]. Increased BBB permeability has also been reported in animals lacking Cx43 expression or its C-terminal domain, which results in microhemorrhages [48].

Cognitive deficits and locomotor impairments are also associated with dysregulation of astrocytic communication. In the hippocampus following MCAO, increased expression of Cx43 and glial fibrillary acidic protein (GFAP) correlates with impaired memory performance measured using the Morris water maze test. Pharmacological inhibition of astrocytes with fluorocitrate restores expression of synaptophysin and CREB-regulated transcription coactivator 1 (CRTC1), improving cognitive function through upregulation of these proteins and suppression of neuroinflammation [68].

In addition to apoptosis, other forms of programmed cell death occur during cerebral ischemia, including necroptosis. In a distal MCAO (dMCAO) model, expression of Cx43 and mixed lineage kinase domain-like protein (MLKL) increases in the ventral posterolateral nucleus of the thalamus as well as in vitro. Interaction of MLKL with Cx43 inhibits K48-linked ubiquitination of Cx43 and induces hemichannel opening, increasing intracellular Ca^2+^ levels and promoting neuronal necroptosis. The E3 ubiquitin ligase Von Hippel–Lindau (VHL) competes with MLKL for binding to Cx43, thereby regulating its degradation [69].

Collectively, the studies reviewed in this section underscore the complex, phase- and subtype-dependent, and often dual role of connexins in the pathogenesis of ischemic and hemorrhagic stroke. While the majority of mechanistic insights derive from MCAO-based models of ischemic stroke, data from ICH and SAH models indicate both shared and distinct connexin-mediated pathways. Depending on the isoform, cellular localization, phosphorylation status, and disease stage, connexins can exert either neuroprotective or neurotoxic effects. A comprehensive overview of the experimental models, connexin isoforms, modulation approaches, key results, and corresponding references is systematically summarized in Table 2.

### 3.2. Role of Connexins in Neurotrauma

#### 3.2.1. Traumatic Brain Injury

**Figure 4:**
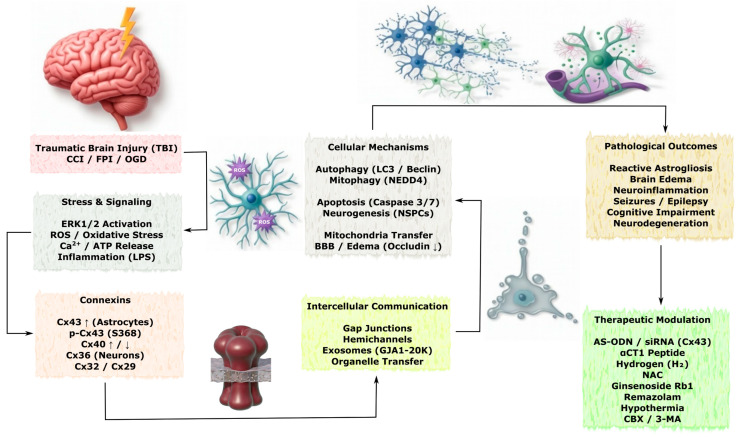
Connexin-Mediated Mechanisms in TBI. This schematic diagram summarizes the role of connexins in the molecular and cellular mechanisms underlying TBI. TBI induces oxidative stress, inflammatory signaling, and activation of intracellular pathways, including ERK1/2 leading to dysregulation and phosphorylation of Cx43, particularly at serine 368 (S368), in astrocytes. Altered Cx43 expression and function modulate GJs, hemichannels, and extracellular vesicle release, thereby affecting intercellular communication. These changes regulate autophagy, mitophagy, mitochondrial transfer, apoptosis, neurogenesis, and BBB integrity. Connexin-dependent signaling contributes to astrogliosis, brain edema, neuroinflammation, epileptogenesis, and cognitive impairment. Therapeutic interventions targeting connexins or related pathways modulate these processes and promote neuroprotection and functional recovery. Arrows indicate direction of change: ↑ increased expression/activity; ↓ decreased expression/activity. Abbreviations: TBI, traumatic brain injury; Cx, connexin; Cx43, connexin 43; Cx40, connexin 40; Cx36, connexin 36; Cx32, connexin 32; Cx29, connexin 29; p-Cx43, phosphorylated connexin 43; S368, serine 368; ERK1/2, extracellular signal-regulated kinase 1/2; ROS, reactive oxygen species; BBB, blood–brain barrier; NSPCs, neural stem/progenitor cells; GJA1-20K, 20-kDa isoform of gap junction alpha-1 protein; LC3, microtubule-associated protein 1 light chain 3; PTZ, pentylenetetrazol; AS-ODN, antisense oligodeoxynucleotide; siRNA, small interfering RNA; CBX, carbenoxolone; 3-MA, 3-methyladenine; NAC, N-acetylcysteine; GS-Rb1, ginsenoside Rb1; H_2_, molecular hydrogen.

TBI is one of the leading causes of disability and mortality worldwide and initiates complex cascades of molecular and cellular events, including alterations in the expression of connexins and the activity of associated GJs. Among them, Cx43, predominantly expressed in astrocytes, plays a fundamental role in the pathogenesis of TBI, whereas other isoforms—Cx36, Cx40, Cx32, and Cx29—participate in diverse processes, demonstrating dynamic functional roles in neurons and glial cells [70]. Cx-dependent mechanisms of intercellular communication and the major pathological consequences of TBI are schematically illustrated in Figure 4.

Given the heterogeneity of TBI mechanisms, the following section distinguishes between closed (non-penetrating) and open (penetrating) injuries and highlights specific preclinical models such as fluid percussion injury (FPI), controlled cortical impact (CCI), weight drop, and needle stab wound. Results obtained from one model should not be directly generalized to all forms of TBI without careful consideration of their specific limitations.

##### Closed (Non-Penetrating) TBI Models

Most available experimental data on Cxs derive from closed TBI models reproducing focal contusion or mixed focal–diffuse injury components, including fluid percussion injury (FPI), controlled cortical impact (CCI), and weight drop models.

Fluid percussion injury

Studies have shown that Cx43 expression markedly increases following TBI, particularly in the hippocampus and cortex, reaching a peak 6–24 h after injury in models of fluid percussion injury (FPI). Immunoreactivity of phosphorylated Cx43 (p-Cx43) in the ipsilateral hippocampus increases within 1 h, peaks at approximately 6 h, and remains elevated for up to 24 h, localizing mainly in astrocytes surrounding pyramidal neurons in the CA3 region. Double immunostaining revealed colocalization of p-Cx43 with phosphorylated ERK (p-ERK), indicating that activation of the ERK1/2 signaling pathway mediates Cx43 phosphorylation and modulates GJ permeability after injury [71].

Other connexins also demonstrate dynamic regulation in FPI models. For example, Cx36 was detected in hippocampal neurons as early as 1 h after TBI but decreased after three days, demonstrating a transient wave-like expression pattern associated with impaired neuronal communication and increased neuronal hyperexcitability [72].

Controlled cortical impact

Elevated Cx43 levels are closely associated with astrogliosis and the development of brain edema after TBI. In experimental studies, treatment with AS-ODNs targeting Cx43 reduced brain water content and inhibited astrocyte proliferation and swelling in a controlled cortical impact (CCI) model, accompanied by decreased Cx43 expression, indicating an important role of Cx43 in regulating post-traumatic cellular water balance [73].

Several studies indicate that Cx43 participates in regulation of autophagy after CCI. In these models, levels of p-Cx43 and LC3-II peaked approximately 6 h after injury in the hippocampus, with LC3-II localized mainly in pyramidal neurons. These observations demonstrate a bidirectional relationship between p-Cx43 and autophagy, since inhibition of p-Cx43 suppressed autophagy, whereas inhibition of autophagy resulted in accumulation of cytotoxic p-Cx43. In addition, activation of autophagy promoted internalization of GJs into the neuronal cytoplasm [74,75].

Cx43 also regulates mitochondrial quality control mechanisms during TBI. Hydrogen therapy (H_2_) effectively suppressed TBI-induced cytotoxic effects by activating mitophagy in lipopolysaccharide (LPS)-treated astrocytes and increasing expression of NEDD4, an E3 ubiquitin ligase that interacts with Cx43 and promotes its ubiquitin-dependent degradation. These changes were accompanied by improved cognitive function, whereas overexpression of Cx43 abolished the H_2_-mediated neuroprotective effects and enhanced apoptotic and inflammatory responses [76].

Alterations in connexin expression after CCI are also associated with oxidative stress pathways. Cx40 expression correlated with markers of oxidative stress, including malondialdehyde (MDA) and nitric oxide (NO), together with decreased levels of glutathione (GSH). Administration of N-acetylcysteine (NAC) reversed these effects, reduced Cx40 expression, and attenuated brain edema and cognitive deficits [77]. Similarly, administration of ginsenoside Rb1 (GS-Rb1) inhibited Cx40 expression through ERK1/2 phosphorylation, reducing contusion volume and neurological deficits, whereas the ERK inhibitor U0126 abolished these effects [78]. In addition, microRNA miR-302 suppresses ERK1/2-mediated phosphorylation of Cx43 in SH-SY5Y cells exposed to pulsatile shear stress, thereby reducing apoptotic cell death and alleviating cognitive deficits associated with TBI-induced brain injury [79].

In addition to its involvement in injury-related mechanisms, connexin signaling also contributes to the regulation of post-traumatic neurogenesis and stem cell responses. Transplantation of neural stem cells (NSCs) into the cortex following CCI increased Cx43 expression at the transplantation site and along the injury border during the early post-traumatic period, suggesting enhanced intercellular communication between NSCs and surrounding brain tissue via Cx43-dependent mechanisms [80]. Cx43 also regulates proliferation and function of neural stem/progenitor cells (NSPCs), which contribute to cognitive recovery after TBI. Increased Cx43 expression was detected in vimentin-positive cells of the subgranular zone of the dentate gyrus following injury. Moreover, treatment of primary NSPC cultures with the α-connexin carboxyl-terminal peptide (αCT1), a selective Cx43 modulator, reduced NSPC proliferation, increased caspase-3/7 expression, and decreased total Cx43 and p-Cx43 (S368) expression in a dose-dependent manner [81].

Weight-drop models

In a free-fall injury model, Cx43 expression increased rapidly and reached a peak within the first day after trauma. In contrast, expression of occludin, a transmembrane protein of tight junctions, decreased and reached minimal levels by day 3 post-TBI. This dynamic correlated with maximal brain edema and pronounced pathological alterations in neural cells, including cellular swelling, degeneration, and apoptotic and necrotic changes [82].

Connexin-associated mechanisms in weight-drop models of TBI are also linked to regulation of autophagy. Administration of BMSCs suppressed expression of Cx43, Beclin-1, and light chain 3 (LC3), thereby reducing autophagic activity in the hippocampus [83]. Similarly, Cx43 contributes to the regulation of neuronal autophagy during cortical stress responses, since pharmacological blockade of GJs with CBX or inhibition of autophagy using 3-methyladenine (3-MA) reduced cognitive impairment and restored long-term potentiation (LTP) in hippocampal slices after TBI. Additionally, CBX, oxidized ATP (OxATP)—a P2X7 receptor (P2X7R) antagonist—and activation of the glutamate transporter GLT-1 by ceftriaxone (Cef) reduced expression of Beclin-1 and increased GLT-1 levels, while CBX additionally suppressed P2X7R expression [84].

Modulation of connexin expression also influences post-traumatic functional recovery. Therapeutic hypothermia, reduced Cx43 expression, and increased levels of the glutamate transporter-1 (GLT-1) in the hippocampus contribute to stabilization of brain water homeostasis and attenuation of cognitive deficits after injury [85].

##### Cx43 in Open/Penetrating and Juvenile TBI Models

In a needle-induced cortical injury model, increased Cx43 expression was detected in the peripheral zone of damage from day 6 to day 15 post-injury, coinciding with reactive astrogliosis. In contrast, Cx43 knockout resulted in a more pronounced inflammatory response characterized by increased activation of astrocytes and microglia, indicating that Cx43 may modulate inflammatory signaling following penetrating brain injury [86].

##### Juvenile TBI

In juvenile TBI (jTBI), inhibition of Cx43 using small interfering RNA (siRNA) reduced reactive astrocyte activation and improved locomotor recovery, although no significant changes in edema were detected using T2-weighted and diffusion-weighted MRI at 1 and 3 days after injury [87].

##### Mechanistic Convergence Across TBI Models

The functional profile of Cx43 is largely determined by its phosphorylation, with S368 representing a key regulatory site. This modification plays a crucial role in the structural and functional organization of GJs. TBI induces phosphorylation of Cx43 at S368, enhancing hemichannel activity and GJ conductance and increasing seizure susceptibility induced by pentylenetetrazol (PTZ). Transgenic modification of this site preventing phosphorylation exerted anticonvulsant effects [88].

Cxs also contribute to intercellular metabolic support after injury. In a compressed gas neuronal injury model, mitochondrial transfer from astrocytes to neurons overexpressing the alternative Cx43 isoform GJA1-20K reduced apoptosis, decreased p-Cx43 levels, and activated neuronal regenerative pathways [5]. Similarly, astrocyte-derived exosomes containing GJA1-20K reduced neuronal apoptosis and stabilized mitochondrial function, whereas exosomes lacking GJA1-20K did not demonstrate neuroprotective effects [89].

Cxs are also involved in regulation of autophagy-related pathways across different TBI models. Colocalization of p-Cx43, P2X7R, and GLT-1 with GFAP further supports the involvement of Cx43-dependent mechanisms in autophagy regulation [84]. Interestingly, another connexin, Cx40, also participates in autophagic processes after TBI. Reduced Cx40 expression correlated with increased formation of autophagic vacuoles, elevated LC3-II and p62 expression, and degenerative neuronal changes. Pharmacological inhibition of autophagy with chloroquine prevented the post-traumatic decrease in Cx40 levels [90].

Pharmacological modulation of Cxs may also influence astrocyte phenotype polarization. Remazol restored cell viability, normalized cytoskeletal organization, and reduced expression of Cx43 and p-Cx43. In OGD/R models simulating traumatic conditions, remazol decreased markers of the pro-inflammatory A1 astrocyte phenotype (C3) while increasing markers of the neuroprotective A2 phenotype (S100A10). In cortical injury models, remazol reduced brain water content, cellular pathological alterations, reactive oxygen species (ROS) levels, and Cx43 expression—predominantly in A2 astrocytes—thereby improving neurological outcomes [91].

Collectively, the studies reviewed in this section demonstrate the complex and model-dependent role of Cxs in TBI. While most data derive from closed non-penetrating models (FPI, CCI, weight drop), results from open/penetrating injuries and juvenile TBI highlight both shared and distinct connexin-mediated pathways. Depending on the isoform, phosphorylation status, and injury model, connexins can exert either neuroprotective or neurotoxic effects. A comprehensive overview of the experimental models, connexin isoforms, modulation approaches, key results, and corresponding references is systematically summarized in Table 3.

#### 3.2.2. Spinal Cord Injury

**Figure 5:**
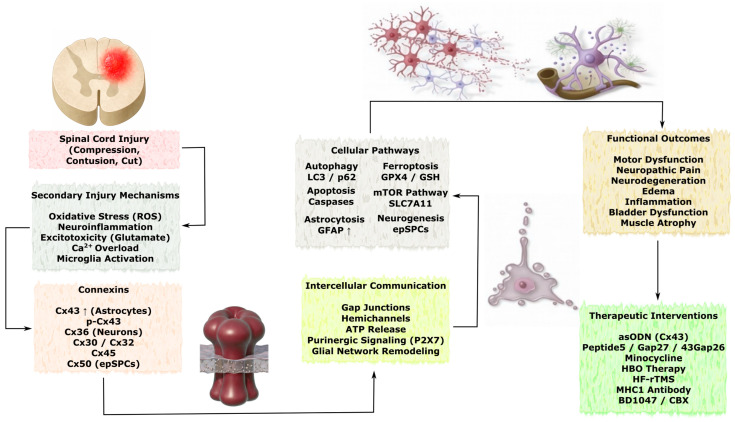
Connexin-Dependent Pathophysiological Mechanisms in SCI. This schematic representation illustrates the involvement of connexins in the pathogenesis of SCI. Mechanical trauma triggers secondary injury processes, including oxidative stress, excitotoxicity, neuroinflammation, and calcium overload, resulting in altered expression and activity of multiple connexins, particularly astrocytic Cx43. Dysregulated Cx43-mediated GJs and hemichannels promote ATP release and purinergic receptor activation, thereby amplifying inflammatory signaling and glial reactivity. Connexin-dependent intercellular communication modulates autophagy, ferroptosis, apoptosis, mTOR signaling, and neurogenesis, influencing tissue remodeling and neuronal survival. Additional connexins, including Cx50 and Cx45, regulate stem/progenitor cell fate and neuromuscular connectivity. These mechanisms contribute to motor dysfunction, neuropathic pain, neurodegeneration, bladder impairment, and muscle atrophy. Pharmacological and physical interventions targeting connexins attenuate secondary damage and improve functional outcomes. Arrows indicate direction of change: ↑ increased expression/activity. Abbreviations: SCI, spinal cord injury; Cx, connexin; Cx43, connexin 43; Cx36, connexin 36; Cx30, connexin 30; Cx32, connexin 32; Cx45, connexin 45; Cx50, connexin 50; p-Cx43, phosphorylated connexin 43; ROS, reactive oxygen species; ATP, adenosine triphosphate;; GFAP, glial fibrillary acidic protein; epSPCs, ependymal stem/progenitor cells; mTOR, mechanistic target of rapamycin; SLC7A11, solute carrier family 7 member 11; GPX4, glutathione peroxidase 4; GSH, glutathione; MDA, malondialdehyde; 4-HNE, 4-hydroxynonenal; HBO, hyperbaric oxygen therapy; HF-rTMS, high-frequency repetitive transcranial magnetic stimulation; CBX, carbenoxolone; AS-ODN, antisense oligodeoxynucleotide; Sig-1R, sigma-1 receptor.

TBI and SCI share several pathological mechanisms, including secondary tissue damage, neuroinflammation, oxidative stress, excitotoxicity, activation of reactive astrocytes and microglia, and neuronal death. However, SCI also possesses unique molecular and cellular pathogenic features, many of which arise from disruption of intercellular communication. In this context, several connexins—including Cx43, Cx36, Cx50, Cx30, Cx32, and Cx45—play active roles. These Cx isoforms exhibit complex expression dynamics and diverse biological effects in astrocytes, neurons, oligodendrocytes, and ependymal cells following SCI [92]. The principal Cx-dependent mechanisms of intercellular communication and the major pathological consequences of SCI are schematically illustrated in Figure 5.

Cx43, the most widely expressed connexin in nervous tissue, plays a central role in the pathogenesis of SCI, exhibiting both neuroprotective and neurodegenerative effects depending on the cellular context and stage of injury. Under physiological conditions, Cx43 contributes to metabolic and signaling homeostasis in the spinal cord. However, traumatic injury to the spinal cord induces cascades of Cx43-dependent processes often associated with reactive astrogliosis, oxidative stress, and exacerbation of neuroinflammation [93]. For example, combined deletion of Cx43/Cx30 significantly reduced neuropathic pain manifestations, including thermal hyperalgesia and mechanical allodynia, following SCI, and was accompanied by reduced astrogliosis. In contrast, deletion of Cx30 alone did not inhibit these processes. Notably, standard treatment with minocycline, an inhibitor of microglial activation, produced a weaker analgesic effect compared with Cx43 deletion [94].

The temporal and spatial dynamics of Cx43 expression after SCI often display a wave-like pattern, reflecting complex mechanisms of intercellular communication remodeling under traumatic stress. In a rat compression injury model, Cx43 immunoreactivity varied depending on the antibodies used and the time after injury. During days 1–3, gray matter regions with moderate neuronal loss exhibited reduced labeling with one antibody, suggesting epitope masking associated with molecular modifications of Cx43. By day 7, Cx43 was absent at the lesion epicenter but was detected in GFAP-positive astrocytes within the subpial rim and around blood vessels, suggesting involvement in tissue reorganization. Colocalization of GFAP/Cx43 indicates a transitional phase in astrocyte activation accompanied by altered Cx43 expression [95].

SCI-induced upregulation of Cx43 is associated with excessive release of ATP through hemichannels, which activates purinergic P2X7R and amplifies neuroinflammation. Studies have shown that deletion of Cx43 combined with Cx30 knockout resulted in reduced ATP release in perilesional regions, accompanied by reduced astrogliosis, reduced microglial activation, reduced lesion volume, and improved motor function. These findings highlight the critical role of astrocytic Cx43 hemichannels in the progression of secondary injury following SCI [10]. In models of spinal cord compression and partial transection, administration of AS-ODN targeting Cx43 reduced edema, astrocyte activation, neutrophil extravasation, and leakage of fluorescently labeled bovine serum albumin, while also improving locomotor function [96].

Similarly, treatment with Peptide5, a mimetic peptide that selectively inhibits Cx43 hemichannels, demonstrated strong protective effects on motoneurons following SCI. This effect was associated with reduced Cx43 expression, increased phosphorylation of Cx43, decreased levels of pro-inflammatory cytokines TNF-α and IL-1β, reduced astrogliosis, and improved hindlimb locomotor recovery [11]. In an ex vivo spinal cord contusion model, the peptides Gap27 and Peptide5 stabilized water homeostasis in neural tissue by decreasing Cx43 expression and reducing astrocyte reactivity. Notably, a dose-dependent effect was observed: low concentrations of Peptide5 prevented hemichannel opening without disrupting GJs, whereas higher concentrations also uncoupled GJ communication [97]. Repeated administration of Peptide5 improved motor function, reduced mechanical allodynia, suppressed neuroinflammation and neuronal death, and decreased levels of both Cx43 and p-Cx43, further confirming inhibition of Cx43-associated hemichannels [12].

Recent studies also suggest that ferroptosis, a form of programmed cell death associated with lipid peroxidation, may be regulated by Cx43-mediated signaling pathways. In SCI models, administration of Gap27 decreased P-mTOR/mTOR expression, restored levels of SLC7A11, increased concentrations of GSH and GPX4, and reduced lipid peroxidation products such as MDA and 4-HNE, thereby alleviating neurological deficits [98]. The use of a chimeric antibody MHC1, which selectively inhibits Cx43 hemichannels, also reduced secondary damage after SCI by decreasing astrocyte activation, stabilizing the lesion area, and preventing neuronal death [99].

As observed in TBI, Cx43 also participates in the regulation of autophagy following spinal cord injury. In spinal cord axotomy models, treatment with HF-rTMS inhibited Cx43 expression while increasing levels of autophagy markers LC3-II and p62 in astrocytes through activation of the mTOR pathway, ultimately improving motor function [100].

Neuropathic pain, a common consequence of SCI, is also associated with Cx43 activity. Evidence suggests a close signaling interaction between Cx43 and sigma-1 receptors (Sig-1R). Inhibition of Cx43 using BD1047, as well as blockade of GJs and hemichannels using carbenoxolone or 43Gap26, significantly reduced allodynia by disrupting Cx43–Sig-1R interactions. Importantly, expression of other connexins, such as Cx32 and Cx36, remained unchanged, supporting the specific role of astrocytic Cx43 in this process [101]. Another study suggests that Cx36 may also contribute to mechanisms associated with neuropathic pain through regulation of glycinergic transmission. However, reduced Cx36 expression in the ipsilateral dorsal horn of the spinal cord was observed not after SCI but in a peripheral nerve injury model. The decrease in Cx36 levels correlated with tactile allodynia, which was further exacerbated by intrathecal injection of Cx36-targeting siRNA but suppressed by the NMDA receptor antagonist MK-801. Notably, Cx36 colocalized with the glycine transporter 2 (GlyT2) in glycinergic interneurons but not with markers of GABAergic neurons [102].

Promising results have also been obtained using HBO which improved neurological outcomes and increased expression of vascular endothelial growth factor (VEGF) while simultaneously reducing both transcription and translation of Cx43 during the early stages after SCI. Interestingly, after two weeks of HBO treatment, Cx43 expression increased significantly, suggesting phase-dependent regulation of Cx43 expression during spinal cord injury [103].

Another connexin, Cx50, expressed in ependymal spinal cord progenitor cells (epSPCs), regulates neurogenesis and cell fate under both physiological and pathological conditions, including spinal cord injury. Overexpression of Cx50 correlated with high levels of GFAP and low expression of Tuj1, promoting glial differentiation. Cx50 was localized in the cytoplasm and nucleus of astrocytes and oligodendrocytes, and its nuclear expression varied depending on the differentiation stage. Cx50 was detected in the gray matter of intact spinal cord and in the injury epicenter after SCI. Transplantation of epSPCs isolated from injured spinal cord with low Cx50 expression accelerated recovery of motor function, suggesting that Cx50 may act as a negative regulator of regenerative processes following SCI [104,105]. In addition, Cx45, together with Cx43, plays an important role in the recovery of connectivity between injured spinal neurons and skeletal muscle. In mouse models with Cx43/Cx45 double knockout, spinal cord axotomy or incomplete contusion resulted in reduced muscle mass loss and improved motor performance. Interestingly, these effects were more pronounced in male than in female mice, suggesting sex-dependent mechanisms of Cx45/Cx43-mediated suppression of neuromuscular interactions after SCI [106].

Finally, in rat models of suprasacral SCI (SSCI) and sacral SCI, expression of Cx43 and c-kit increased in the SSCI group and correlated with detrusor hyperreflexia, whereas the opposite pattern was observed in rats with sacral SCI. These findings indicate complex regulatory mechanisms controlling bladder excitability following SCI mediated by Cx43-dependent signaling pathways [107].

Collectively, the studies reviewed in this section demonstrate the complex and model-dependent role of connexins in spinal cord injury. While most data derive from compression and contusion models, findings from axotomy and ependymal progenitor studies highlight both shared and distinct connexin-mediated pathways. Depending on the isoform, cellular context, and stage of injury, connexins can exert either neuroprotective or neurotoxic effects. A comprehensive overview of the experimental models, connexin isoforms, modulation approaches, key results, and corresponding references is systematically summarized in Table 4.

#### 3.2.3. Peripheral Nerve Injury

**Figure 6:**
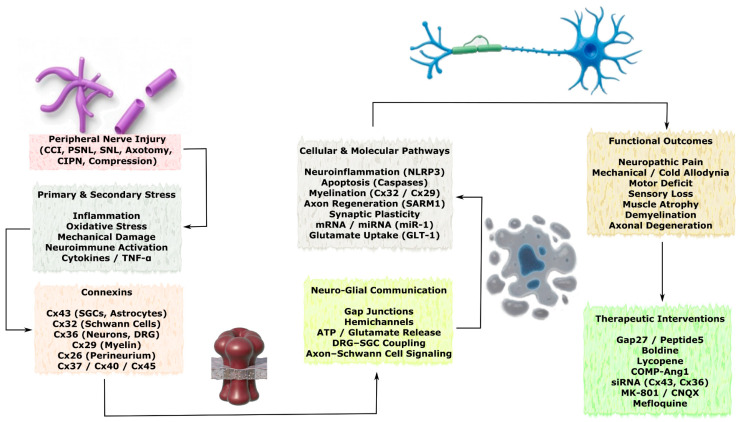
Connexin-Mediated Mechanisms in PNI. This schematic diagram illustrates the role of connexins in the molecular and cellular mechanisms underlying PNI. Traumatic, compressive, or chemically induced nerve damage triggers inflammatory and oxidative stress responses, leading to dysregulation of multiple connexins in neurons, satellite glial cells, Schwann cells, and astrocytes. Altered expression and function of Cx43, Cx32, Cx36, and other connexins modulate GJs and hemichannels, thereby affecting neuro–glial communication, ATP and glutamate signaling, and inflammasome activation. Connexin-dependent pathways regulate myelination, axonal degeneration and regeneration, synaptic plasticity, and microRNA-mediated gene expression. These mechanisms contribute to neuropathic pain, sensory and motor deficits, demyelination, and muscle atrophy. Pharmacological and genetic interventions targeting connexins or related signaling pathways attenuate inflammation, restore intercellular communication, and promote functional recovery. Abbreviations: PNI, peripheral nerve injury; Cx, connexin; Cx43, connexin 43; Cx32, connexin 32; Cx36, connexin 36; Cx29, connexin 29; Cx26, connexin 26; Cx37, connexin 37; Cx40, connexin 40; Cx45, connexin 45; DRG, dorsal root ganglion; SGCs, satellite glial cells; ATP, adenosine triphosphate; BDNF, brain-derived neurotrophic factor; miR-1, microRNA-1; NLRP3, NOD-like receptor family pyrin domain-containing 3; GLT-1, glutamate transporter 1; SARM1, sterile alpha and TIR motif-containing protein 1; ROS, reactive oxygen species; TNF-α, tumor necrosis factor alpha; CCI, chronic constriction injury; PSNL, partial sciatic nerve ligation; SNL, spinal nerve ligation; CIPN, chemotherapy-induced peripheral neuropathy; CFA, complete Freund’s adjuvant; COMP-Ang1, cartilage oligomeric matrix protein–angiopoietin-1; siRNA, small interfering RNA.

Peripheral nerve injury (PNI) initiates a complex cascade of molecular and cellular events associated with multiple neurological disturbances, including neuropathic pain as well as sensory and motor deficits, which may result in severe health consequences ranging from disability to mortality. In the peripheral nervous system (PNS), intercellular communication mediated by Cx-associated hemichannels and gap junctions plays an essential role under both physiological conditions and pathological states related to neurotrauma [108]. Connexins such as Cx43, Cx32, Cx36, Cx29, Cx37, Cx26, Cx40, and Cx45 influence inflammatory responses, neuropathic pain, axonal regeneration, and restoration of neuromuscular connectivity following peripheral nerve injuries. The principal Cx-dependent mechanisms of intercellular communication and pathological consequences of PNI are schematically illustrated in Figure 6.

In a CCI model of the sciatic nerve, increased expression of Cx43 and brain-derived neurotrophic factor (BDNF) was observed together with decreased levels of miR-1. These findings suggest that miR-1 may act as a negative post-transcriptional regulator of Cx43 and BDNF expression and thereby contribute to the development of neuropathic alterations [109]. In a model of inferior alveolar nerve injury, persistent mechanical allodynia in the vibrissal pad and upper eyelid skin was associated with activation of SGCs and increased Cx43 expression in the trigeminal ganglion. Cx43 expression was detected in SGCs surrounding neurons innervating the vibrissal pad, and inhibition of Cx43-associated GJs using Gap27 reduced both SGC activation and allodynia [110]. Similarly, in another study, CCI of the sciatic nerve induced increased Cx43 expression in the spinal cord, which was suppressed by intrathecal administration of Peptide5, effectively reducing mechanical hypersensitivity, inflammatory responses, and levels of NLRP3 inflammasome components. However, Peptide5 therapy was ineffective in CIPN caused by oxaliplatin or paclitaxel, likely because its neuroprotective effects partially depend on modulation of NLRP3 signaling, which remained unchanged in these models [111]. Oral administration of boldine, an inhibitor of connexin hemichannels, significantly reduced expression of Cx43 and Cx45 in Schwann cells and attenuated muscle atrophy after peroneal nerve transection. Combined nerve cross-repair with boldine treatment increased nerve conduction velocity and promoted structural and functional recovery of muscle fibers [112].

In the PSNL model, decreased Cx43 expression was observed in the ipsilateral dorsal horn of the spinal cord and was associated with mechanical hypersensitivity. This hypersensitivity was further exacerbated by intrathecal administration of Cx43-targeting siRNA. These effects were partially reversed by the glutamate receptor antagonists MK-801 and CNQX. Reduced Cx43 levels were accompanied by decreased expression of the glutamate transporter GLT-1 and impaired glutamate uptake [113]. In another PSNL study, Cx43 expression decreased in spinal astrocytes but was restored by treatment with lycopene, a non-provitamin carotenoid, which inhibited TNF-dependent suppression of Cx43 expression and reduced neuropathic pain [114]. Conversely, in a spinal nerve ligation (SNL) model, intrathecal Cx43-targeting siRNA reduced hypersensitivity and correlated with decreased Cx43 expression, while levels of Cx36 and GFAP remained unchanged [115]. These findings suggest that pathological signaling during PNI may regulate Cx43 expression in spinal astrocytes.

Cx32, produced by Schwann cells and oligodendrocytes, occupies a central position in the mechanisms of myelination in the PNS, both under physiological conditions and during neuronal stress induced by traumatic injury. Mutations in the gene encoding Cx32 cause extensive demyelinating neuropathies in the PNS [15,116]. In healthy sciatic nerve, Cx32 is localized at the nodes of Ranvier and Schmidt–Lanterman incisures, where it maintains axonal homeostasis. Experimental studies have demonstrated that Cx32 knockout leads to progressive demyelinating neuropathy primarily affecting motor fibers, whereas no abnormalities in CNS myelin were observed, indicating the specificity of Cx32-dependent demyelination processes for the PNS [15]. Similarly, mutations of Cx32 in transgenic mice produced demyelination in both the PNS and CNS. The mutant Cx32 protein displayed abnormal intracellular localization and failed to form characteristic GJ plaques. Importantly, expression and localization of Cx47 and Cx29 in glial cells remained unchanged, suggesting loss of function of mutant Cx32 without transdominant effects on other connexins [116].

Distinct patterns of Cx expression have also been described in peripheral nerves. In the sciatic nerve, Cx26 is expressed in the perineurium, Cx43 in the perineurium and epineurium, and Cx32 in paranodal regions of the nodes of Ranvier. Following injury, these connexins exhibit stress-induced dynamic changes. For example, Cx32 expression temporarily disappears and subsequently recovers, whereas Cx43 expression rapidly increases and then decreases, showing a pattern similar to fluctuations in Cx26 levels. These findings indicate a wave-like pattern of Cx26, Cx32, and Cx43 expression in response to traumatic injury in the PNS [117]. In a model of diabetic peripheral neuropathy, treatment with COMP-Ang-1 increased expression of Cx32 and Cx26 and promoted regeneration of endoneurial microvessels in sciatic nerve fibers, while simultaneously reducing inflammation, decreasing Cx43 expression, and limiting axonal degeneration through restoration of GJ communication [118]. Mutations in Cx26 have also been associated with sensorineural deafness accompanied by demyelination, degeneration of spiral ganglion neurons, and abnormal neural innervation [119].

Notably, interesting findings have been reported regarding Cx36 expression in dorsal root ganglia (DRG). In DRG neurons and SGCs, Cx36 was detected with uniform staining in cell bodies and membranes and colocalized with β-III tubulin and glutamine synthetase, whereas Cx43 was detected exclusively in SGCs. CCI reduced Cx36 mRNA levels in DRG, which contributed to neuropathic pain development [120]. In oxaliplatin-induced CIPN, genetic deletion of Cx36 suppressed tactile hypersensitivity, suggesting that Cx36 modulates pain through neuronal GJ signaling [121].

In a partial infraorbital nerve transection (pT-ION) model, the Cx36 inhibitor mefloquine attenuated cold allodynia, although it did not affect mechanical allodynia. This effect was associated with suppression of pT-ION-induced increases in Cx36, ionotropic kainate receptor 2 (GluK2), transient receptor potential ankyrin 1 (TRPA1), and p-ERK expression in the trigeminal ganglion. Selective suppression of Cx36 in Nav1.8-positive nociceptors also reduced cold allodynia, confirming the involvement of Cx36 in orofacial pain via glutamatergic and TRPA1 signaling pathways [122]. Following axotomy, motor neurons become electrically coupled through GJs associated with Cx36, Cx37, Cx40, Cx43, and Cx45, which may support regenerative processes until reinnervation occurs [123,124]. In addition, SARM1, an enzyme responsible for axon degeneration, is regulated through Cx43 in HEK-293T cells and through Cx36 in DRG neurons. Knockout of Cx36 resulted in axonal degeneration during neuroinflammation due to disruption of Cx36-mediated negative regulation of SARM1 expression [125].

Cx29, produced by Schwann cells and oligodendrocytes, is localized in deeper layers of sciatic nerve myelin, unlike Cx32 which is located in the outer myelin layers. Interestingly, Cx29 has been detected near hexagonal “rosettes” of intramembranous particles (IMPs), suggesting unique functions distinct from classical GJ-associated roles. Notably, knockout of Cx32 did not alter Cx29 levels, indicating an independent functional role [126]. In immortalized mouse neuroblastoma cell lines, Cx29 and Cx32 were co-expressed and participated in the structural organization of GJs [127].

In addition, CCI of the sciatic nerve induced increased Cx37 mRNA expression in both proximal and distal nerve segments at 1–2 weeks after injury, contributing to thermal hyperalgesia. Subsequently, Cx37 expression returned to baseline levels as neuropathic pain decreased [128]. Finally, increased expression of Cx26, Cx36, and Cx40, but not Cx43, was observed in neurons and SGCs of the trigeminal ganglion following injections of capsaicin or complete Freund’s adjuvant (CFA) into the temporomandibular joint. This upregulation likely represents an adaptive response to acute or chronic inflammation aimed at enhancing neuroglial communication [129].

Collectively, the studies reviewed in this section demonstrate the complex and context-dependent role of connexins in PNI. Cx43 is prominently involved in neuroinflammation, glial activation, and neuropathic pain in the DRG and spinal cord, while Cx32 plays a central role in myelination and demyelinating neuropathies. Other isoforms, including Cx36, Cx29, Cx37, and Cx26, contribute to neuronal coupling, axonal regeneration, and pain signaling in a model- and cell-type-specific manner. A comprehensive overview of the experimental models, connexin isoforms, modulation approaches, key results, and corresponding references is systematically summarized in Table 5.

### 3.3. Role of Connexins in Neurodegenerative Diseases

Although acute brain injuries such as stroke and TBI are characterized by rapid onset of tissue damage, many of the underlying molecular and cellular mechanisms overlap with those observed in chronic neurodegenerative disorders. Connexin-mediated intercellular communication represents one of the shared pathways linking these conditions, as GJs and hemichannels regulate astrocyte reactivity, neuroinflammatory signaling, calcium wave propagation, metabolic coupling, and mitochondrial homeostasis. In acute injury models, dysregulation of connexins contributes to propagation of excitotoxic and inflammatory signals within the neurovascular unit, whereas in chronic neurodegenerative diseases similar mechanisms may facilitate the spread of protein aggregation–associated stress, glial activation, and progressive neuronal dysfunction. Importantly, in disorders such as Alzheimer’s disease, Parkinson’s disease, amyotrophic lateral sclerosis, and Huntington’s disease, alterations in connexin expression and function are often considered secondary responses to pathological processes including protein misfolding, chronic inflammation, synaptic dysfunction, and neuronal loss. Therefore, connexin-dependent pathways are more commonly interpreted as modulators of disease progression and intercellular propagation of pathology rather than primary initiating mechanisms. Understanding these shared signaling principles may help identify convergent therapeutic targets capable of modulating both acute injury responses and chronic neurodegenerative processes.

In this section, the available experimental and clinical evidence describing connexin-associated mechanisms in major neurodegenerative disorders is systematically reviewed, with particular emphasis on Alzheimer’s disease, Parkinson’s disease, amyotrophic lateral sclerosis, and Huntington’s disease. Special attention is given to the cell-type-specific expression of connexins in astrocytes, neurons, oligodendrocytes, and vascular cells, as well as to their involvement in neuroinflammation, protein aggregation–associated toxicity, mitochondrial dysfunction, impaired neuroglial coupling, and progressive synaptic and neuronal loss. Where possible, distinctions are made between mechanisms that may contribute to the amplification and spread of pathology and those that may represent compensatory or neuroprotective responses.

#### 3.3.1. Alzheimer’s Disease

**Figure 7:**
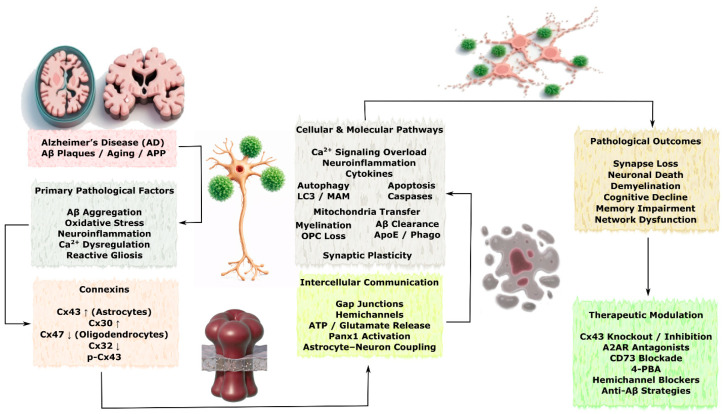
Connexin-Mediated Mechanisms in AD. This schematic diagram summarizes the role of connexins in the molecular and cellular mechanisms underlying AD. Accumulation of amyloid-β (Aβ) peptides and age-related stress induce astrocyte and glial activation, leading to dysregulated expression and phosphorylation of connexins, particularly Cx43 and Cx30 accompanied by reduced levels of Cx47 and Cx32 in oligodendrocytes. Altered connexin-mediated GJs and hemichannels promote excessive release of ATP and glutamate, intracellular calcium (Ca^2+^) overload, and pannexin-1 (Panx1) activation, thereby amplifying neuroinflammatory signaling. Connexin-dependent pathways regulate mitochondrial-associated membrane (MAM) formation, autophagy, mitochondrial transfer, amyloid clearance, synaptic plasticity, and myelin maintenance. Interactions between Cx43, adenosine A2A receptors (A2AR), and CD73 generate potential positive feedback loops that exacerbate amyloid toxicity and neurodegeneration. These mechanisms contribute to synapse loss, neuronal death, demyelination, and progressive cognitive decline. Therapeutic strategies targeting connexins and related signaling pathways may attenuate neuroinflammation and promote neuroprotection in AD. Arrows indicate direction of change: ↑ increased expression/activity; ↓ decreased expression/activity. Abbreviations: AD, Alzheimer’s disease; Aβ, amyloid-beta; Cx, connexin; Cx43, connexin 43; Cx30, connexin 30; Cx47, connexin 47; Cx32, connexin 32; p-Cx43, phosphorylated connexin 43; ATP, adenosine triphosphate; Ca^2+^, calcium ion; Panx1, pannexin-1; ROS, reactive oxygen species; MAM, mitochondria-associated membranes; MFN2, mitofusin-2; ApoE, apolipoprotein E; OPCs, oligodendrocyte progenitor cells; A2AR, adenosine A2A receptor; CD73, ecto-5′-nucleotidase; 4-PBA, 4-phenylbutyric acid.

Alzheimer’s disease (AD) is a neurodegenerative disorder characterized by progressive cognitive decline accompanied by accumulation of amyloid plaques, neuronal loss, and reactive gliosis, in which astrocytes play a central role. Numerous studies have demonstrated the important involvement of connexins in the progression and modulation of AD-related pathology, with Cx43 representing one of the most extensively studied isoforms, along with Cx30 and Cx47, which participate in complex intra- and intercellular signaling mechanisms [130]. The principal Cx-dependent mechanisms of intercellular communication and pathological consequences of AD are schematically illustrated in Figure 7.

Studies have shown that significant upregulation of astrocytic Cx43 and Cx30 in the cortex and thalamus of aged animals correlates with Aβ deposition, increased numbers of reactive astrocytes, neuronal degeneration and death, and cognitive impairment associated with memory dysfunction. In contrast, expression of Cx47 and its colocalization with Cx43 were reduced, indicating disruption of astrocyte–oligodendrocyte communication. This was accompanied by depletion of OPCs and mature oligodendrocytes, as well as myelin deficits. It has been proposed that alterations in Cx43-mediated signaling may shift neuronal homeostasis toward impaired myelination and enhanced Aβ accumulation, suggesting involvement of connexin-dependent mechanisms in disease progression rather than initiation [18]. Interestingly, in the spinal cord of the 5XFAD mouse model of AD, similar processes were observed, including accumulation of Cx43 and Cx30 localized around amyloid plaques. However, in this study, Cx47 expression in oligodendrocytes increased, while Cx32 expression tended to decrease, and myelin deficits were detected in areas with Aβ aggregation [131].

In an experimental model mimicking familial AD, activation of astrocytic hemichannels associated with Cx43 was observed in hippocampal slices containing Aβ plaques. In a subpopulation of reactive astrocytes directly contacting Aβ plaques, activity of Panx1 hemichannels was detected and correlated with neuroinflammation. Activation of Cx43 hemichannels, in turn, occurred through Ca^2+^-dependent mechanisms, potentially contributing to a feed-forward cycle involving release of ATP and glutamate through these channels followed by intracellular Ca^2+^ accumulation. Cx43 knockout was associated with protective effects, reducing neuroinflammation, oxidative stress, and neuronal degeneration, indicating that hemichannel activity may contribute to amplification of pathological signaling in the hippocampus [21]. Genetic deletion of Cx43 or pharmacological blockade of its hemichannels using the peptide TAT-Cx43^266–283 was also shown to reduce reactive microglia and attenuate Aβ-induced neurotoxicity and cognitive impairment [132].

Similar findings were obtained in astrocyte cultures exposed to the peptide Aβ25–35, which caused structural and functional disruption of Cx43 GJs, increased hemichannel activity, and elevated intracellular Ca^2+^ levels. These effects were reversed by treatment with 4-phenylbutyrate, which restored Cx43-mediated astrocytic intercellular communication. Molecular modeling also suggested the possibility of direct interaction between Aβ and Cx43 [133]. In the APP/PS1 mouse model, animals older than four months showed increased expression of Cx43 and Cx30, both localized near dense Aβ plaques. This phenomenon intensified with age and did not correlate with microglial activation [134]. In the same model, Cx43 knockout significantly reduced astrogliosis and increased synaptic density while attenuating cognitive impairment [135].

In cortical tissue from patients with AD, elevated Cx43 expression has been detected in regions containing β/A4-amyloid plaque aggregates. Cx43 was localized predominantly within astrocytic GJs, suggesting a dual functional role: it may contribute to neuroprotection by stabilizing neuronal homeostasis but may also exert cytotoxic effects that sustain Aβ-mediated neuronal degeneration [136]. Another study demonstrated a close association between AD and transcription of the gene encoding Cx43, which appears to participate in regulatory networks controlling numerous Aβ-related genes. Astrocytes lacking Cx43 exhibited reduced levels of apolipoprotein E (ApoE), impaired Aβ phagocytosis, and increased Aβ accumulation in co-cultured neurons. However, under Aβ-induced stress, these astrocytes displayed pronounced neuroprotective effects, indicating complex and context-dependent roles of Cx43 in AD pathogenesis [137].

Cx43 has also been implicated in regulation of MAMs, which are altered in AD. In a mouse model of AD, expression of Cx43 and mitofusin-2 (MFN2)—a biomarker of MAMs—was significantly increased, and strong colocalization between these proteins was observed. Knockout of Cx43 reduced MAM contacts and exerted neuroprotective effects by activating autophagy, leading to decreased Aβ levels, reduced neuroinflammation, and diminished neuronal apoptosis [138]. Moreover, mitochondrial transfer from astrocytes to neurons and endothelial cells mediated by CD38 and Cx43 was enhanced under Aβ-induced stress. Increased numbers of CD38- and Cx43-positive cells were detected in the hippocampus, in in vitro blood–brain barrier models, and in isolated astrocytes [139]. Cx43-associated hemichannels are also modulated by purinergic signaling, including A2AR. Increased expression of both Cx43 and A2AR has been detected in the brains of patients with AD. In an in vitro AD model, astrocytes exposed to Aβ1–42 exhibited increased activity of Cx43 hemichannels that released ATP; this effect could be blocked by A2AR inhibitors. A2AR regulates both Cx43 expression and its phosphorylated form under Aβ-associated toxicity, while inhibition of CD73, which converts ATP to adenosine, reduced Cx43 hemichannel activity. Notably, A2AR and Cx43 can form direct physical interactions, suggesting the presence of regulatory feedback mechanisms that may amplify neurodegenerative signaling in AD [140]. Another study demonstrated that inhibition of A2AR in hippocampal slices from mice receiving intracerebroventricular injection of Aβ1–42 or in the APP/PS1 model prevented excessive activation of Cx43 hemichannels, whereas under control conditions A2AR inhibition increased their activity [141].

Collectively, the studies reviewed in this section highlight the complex and often dual role of connexins, particularly Cx43 and Cx30, in the pathogenesis of Alzheimer’s disease. While upregulation of Cx43 and Cx30 in reactive astrocytes is frequently associated with neuroinflammation, impaired glutamate uptake, and synaptic dysfunction around amyloid plaques, other connexins such as Cx47 and Cx32 show decreased expression, suggesting disruption of astrocyte–oligodendrocyte communication and myelin maintenance. Depending on the cellular context, disease stage, and experimental model, connexin-mediated signaling can either exacerbate Aβ toxicity and neuronal death or exert protective effects through regulation of autophagy, mitochondrial function, and intercellular communication. A comprehensive overview of the experimental models, connexin isoforms, modulation approaches, key results, and corresponding references is systematically summarized in Table 6.

#### 3.3.2. Parkinson’s Disease

**Figure 8:**
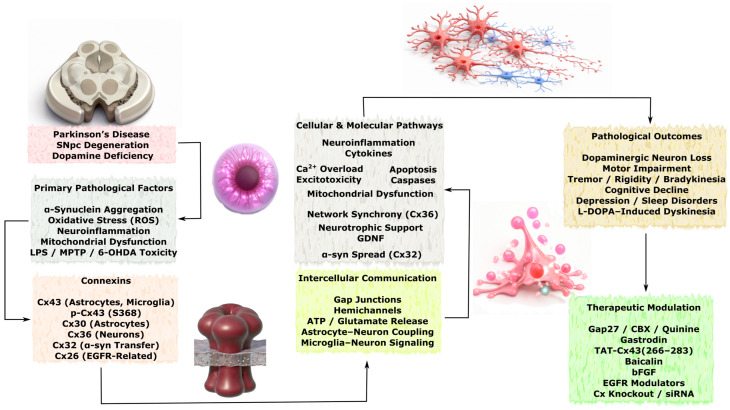
Connexin-Mediated Mechanisms in PD. This schematic diagram summarizes the role of connexins in the molecular and cellular mechanisms underlying PD. Degeneration of dopaminergic neurons in the substantia nigra pars compacta (SNpc) and dopamine deficiency are associated with oxidative stress, mitochondrial dysfunction, α-synuclein (α-syn) aggregation, and neuroinflammatory responses. These processes lead to dysregulated expression and phosphorylation of multiple connexins, particularly Cx43, Cx30, Cx36, Cx32, and Cx26, in astrocytes, microglia, and neurons. Altered connexin-mediated GJs and hemichannels may promote excessive release of ATP and glutamate, Ca^2+^ overload, and amplification of inflammatory signaling. Connexin-dependent pathways regulate neuronal network synchronization, mitochondrial integrity, α-syn transfer, and neurotrophic support. These mechanisms are associated with dopaminergic neuron vulnerability, motor impairment, cognitive and affective disturbances, and L-DOPA–induced dyskinesia. Pharmacological and genetic modulation of connexins and related signaling pathways may attenuate neuroinflammation and enhance neuroprotection in PD. Abbreviations: PD, Parkinson’s disease; SNpc, substantia nigra pars compacta; Cx, connexin; Cx43, connexin 43; Cx30, connexin 30; Cx36, connexin 36; Cx32, connexin 32; Cx26, connexin 26; p-Cx43, phosphorylated connexin 43; ATP, adenosine triphosphate; Ca^2+^, calcium ion; α-syn, alpha-synuclein; ROS, reactive oxygen species; LPS, lipopolysaccharide; MPTP, 1-methyl-4-phenyl-1,2,3,6-tetrahydropyridine; 6-OHDA, 6-hydroxydopamine; GDNF, glial cell line-derived neurotrophic factor; bFGF, basic fibroblast growth factor; CBX, carbenoxolone; EGFR, epidermal growth factor receptor; LID, L-DOPA–induced dyskinesia.

Parkinson’s disease (PD) is a common neurodegenerative disorder characterized by progressive loss of dopaminergic neurons in the substantia nigra, leading to gradual impairment of motor and cognitive functions. While the primary pathological hallmarks of PD include α-synuclein aggregation, mitochondrial dysfunction, and progressive degeneration of nigrostriatal dopaminergic pathways, alterations in connexin-mediated intercellular communication have been increasingly associated with mechanisms that may influence neuroinflammation, neuronal vulnerability, and network dysfunction. Several connexin isoforms, including Cx43, Cx30, Cx36, and Cx32, have been implicated in processes related to neuroinflammatory signaling, oxidative stress, and neuronal network synchronization in PD models [142]. The principal Cx-dependent mechanisms of intercellular communication and pathological consequences of PD are illustrated in Figure 8.

In patients with advanced PD, a significant reduction in Cx43 expression has been observed in the cortex and basal ganglia, accompanied by degeneration of the astrocytic network in the frontal cortex and correlated with symptoms such as depression and insomnia. Disruption of astrocytic coupling has therefore been proposed as one of the factors potentially contributing to disease progression, rather than a primary pathogenic trigger [143]. Conversely, in a rotenone-induced PD model, increased expression and phosphorylation of Cx43 were detected in the basal ganglia, particularly in the substantia nigra pars reticulata and the globus pallidus, with the increase occurring primarily through post-transcriptional regulation. This change was associated with enhanced intercellular communication mediated by Cx43-dependent mechanisms [144]. Interestingly, gastrodin, an active compound derived from traditional Chinese herbal medicine, reduced Cx43 levels and inhibited its phosphorylation in the striatum and hippocampus, exerting a neuroprotective effect in PD models [145].

Another study demonstrated that intranigral injections of LPS, used to induce PD-like pathology, decreased total Cx43 expression while increasing p-Cx43, leading to hyperactivation of hemichannels and a subsequent neurotoxic cascade caused by excessive ATP and glutamate release. Administration of Gap27 prevented dopaminergic neuronal death and restored dopamine and its metabolites by inhibiting Cx43 activity and neuroinflammation while activating neurotrophic neuroprotective mechanisms in PD [22]. Similar results were observed in 6-OHDA models of PD. In these models, degeneration of dopaminergic neurons was accompanied by divergent changes in Cx43 and phosphorylated Cx43 (Cx43-pS368) expression. Total Cx43 levels increased, whereas Cx43-pS368 levels decreased. These effects were attenuated by Gap27, further demonstrating a neuroprotective role of connexin inhibition in PD [146].

Another connexin, Cx30, also exhibits diverse effects in PD. Knockout of Cx30 accelerated loss of dopaminergic neurons in the striatum in a PD model induced by MPTP. This was accompanied by reduced astrogliosis, while microglial activation remained unaffected. At the same time, expression of pan-reactive astrocytic genes and the neurotrophic factor GDNF decreased. These findings suggest that Cx30-dependent signaling pathways are essential for astrocyte-mediated neuroprotection during PD-associated neurodegeneration [147]. In the same PD model, administration of bFGF into the striatum increased Cx43 expression and the number of Cx43-positive puncta near the implantation site, although functional astrocytic coupling was not enhanced [148].

Alterations in Cx36 expression have also been reported in PD models. Increased Cx36 levels were detected in the striatum and motor cortex, particularly in enkephalin-positive (ENK^+^) medium spiny neurons, whereas Cx36 expression decreased in parvalbumin-positive (PV^+^) interneurons in the 6-OHDA PD model. Such an imbalance may contribute to desynchronization between the cortex and basal ganglia, thereby promoting PD progression [149]. In another study, Cx36 expression was found to be higher in LID than in PD or control conditions in the striatum and motor cortex, particularly in ENK^+^ and PV^+^ neurons. Pharmacological blockade of GJs using carbenoxolone or quinine alleviated abnormal involuntary movements [150]. Additional studies have shown that Cx36 and tyrosine hydroxylase expression decrease in the cortex and striatum after 6-OHDA injections but increase following treatment with the neuroprotective compound baicalin [151].

Cx32 also plays a distinctive role in neurodegenerative diseases including PD. Both in vitro and in vivo studies have demonstrated that Cx32 may contribute to PD progression by facilitating uptake and intercellular transfer of α-syn. Direct interaction between Cx32 and α-synuclein has been confirmed in postmortem brain samples from patients with PD [152]. Additionally, increased expression of Cx36 has been detected in the putamen, external and internal segments of the globus pallidus, but not in the subthalamic nucleus. Increased GJ activity and number in these regions enhanced synchronization of neuronal activity, which becomes particularly pronounced under conditions of dopamine deficiency and may contribute to the progression of motor symptoms in PD [153].

Finally, notable findings have been reported regarding Cx26. In an in vitro PD model induced by MPP^+^, increased expression of genes associated with the EGFR signaling pathway, including GJB2 Cx26, was observed in SH-SY5Y neurons. These results suggest that EGFR signaling and Cx26 expression may contribute to neuronal degeneration in PD-like conditions [154].

Collectively, the studies reviewed in this section highlight the complex and often dual role of connexins in Parkinson’s disease. While Cx43 is the most extensively studied isoform and is frequently associated with neuroinflammation and dopaminergic neuron vulnerability, other Cxs such as Cx36, Cx30, and Cx32 also play important roles in neuronal synchronization, glial support, and α-synuclein transfer. Most data derive from toxin-based models (LPS, 6-OHDA, MPTP, rotenone), while findings from postmortem human brain and genetic studies provide additional insights. Depending on the isoform, cellular localization, and disease stage, connexins can exert either neuroprotective or neurotoxic effects. A comprehensive overview of the experimental models, connexin isoforms, modulation approaches, key results, and corresponding references is systematically summarized in Table 7.

#### 3.3.3. Amyotrophic Lateral Sclerosis

**Figure 9:**
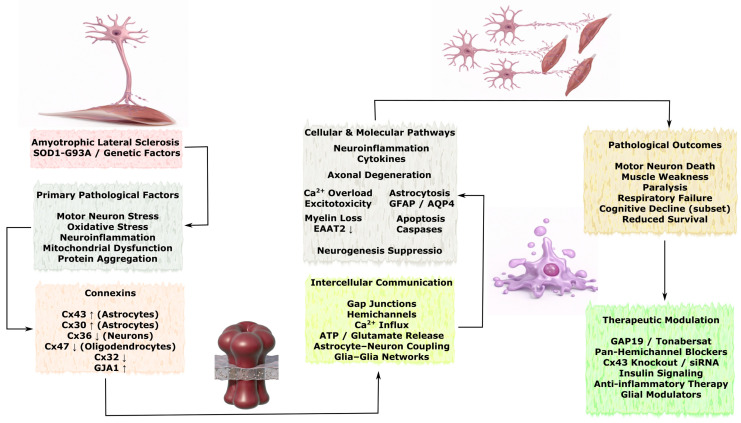
Connexin-Mediated Mechanisms in ALS. This schematic diagram summarizes the role of connexins in the molecular and cellular mechanisms underlying ALS. Genetic and molecular stressors, including mutant superoxide dismutase 1 (SOD1-G93A), are associated with progressive degeneration of motor neurons and activation of astrocytes, microglia, and oligodendrocytes. These processes lead to dysregulated expression of multiple connexins, particularly astrocytic Cx43 and Cx30, accompanied by reduced levels of Cx36, Cx47, and Cx32. Altered connexin-mediated GJs and hemichannels may promote dysregulated calcium (Ca^2+^) signaling and increased ATP and glutamate release, and amplification of neuroinflammation. Connexin-dependent pathways regulate excitotoxicity, mitochondrial dysfunction, axonal degeneration, and astrocytic water homeostasis. These mechanisms are associated with increased motor neuron vulnerability, progressive muscle weakness, paralysis, and reduced survival. Pharmacological and genetic modulation of connexins, including hemichannel blockers and Cx43 inhibition, attenuates neuroinflammation and promotes neuroprotection in experimental models of ALS. Arrows indicate direction of change: ↑ increased expression/activity; ↓ decreased expression/activity. Abbreviations: ALS, amyotrophic lateral sclerosis; Cx, connexin; Cx43, connexin 43; Cx30, connexin 30; Cx36, connexin 36; Cx47, connexin 47; Cx32, connexin 32; SOD1, superoxide dismutase 1; SOD1-G93A, glycine-to-alanine substitution at position 93 of SOD1; GJA1, gap junction protein alpha 1; ATP, adenosine triphosphate; Ca^2+^, calcium ion; EAAT2, excitatory amino acid transporter 2; GFAP, glial fibrillary acidic protein; AQP4, aquaporin 4; iPSC, induced pluripotent stem cell; GAP19, connexin 43 hemichannel inhibitory peptide; ROS, reactive oxygen species.

Amyotrophic lateral sclerosis (ALS) is a severe neurodegenerative disease characterized by progressive degeneration of motor neurons in the CNS, ultimately leading to paralysis and death. Astrocytes play a major role in ALS pathogenesis through multiple mechanisms, including intercellular communication with neurons. Numerous studies have demonstrated alterations in connexin expression associated with astrocyte reactivity and neuroinflammatory signaling, particularly involving Cx43, Cx30, Cx36, Cx47, and Cx32 in ALS pathophysiology. The principal Cx-dependent mechanisms of intercellular communication and pathological consequences of ALS are summarized in Figure 9.

For example, the SOD1 G93A mutation causes progressive upregulation of Cx43 expression in the spinal cord of mice and in both the motor cortex and spinal cord of patients with ALS. Increased Cx43 expression has also been observed in astrocytes isolated from SOD1 G93A mice and in astrocytes derived from iPSCs of ALS patients. This increase correlates with enhanced GJ coupling, increased hemichannel activity, and elevated intracellular Ca^2+^ levels. Importantly, inhibition of Cx43 using pan-connexin blockers or selective Cx43 hemichannel inhibitors such as Gap19 and tonabersat produced neuroprotective effects in co-culture systems of motoneurons and SOD1 G93A astrocytes [23,155,156]. Long-term treatment with tonabersat reduced astrogliosis and microgliosis and decreased neuronal death in SOD1 G93A mice, producing effects similar to those observed in Cx43 knockout models [155]. In patients with ALS, increased expression of the GJA1 gene (encoding Cx43) has been detected in motoneurons and correlates with neuroinflammatory processes, whereas genes negatively correlated with GJA1 are associated with neurogenesis-related pathways [157]. It has been proposed that degeneration of motoneurons activates glial cells, which in turn induce Cx43 overexpression, potentially contributing to amplification of neurotoxic signaling and formation of a feed-forward pathogenic loop in ALS [158].

In a SOD1-mutant mouse model of ALS, high expression of Cx30 was detected even before the onset of clinical symptoms. Deletion of Cx30 exerted neuroprotective effects, delaying disease progression, preserving motor neurons in the anterior horn of the spinal cord, and reducing neuroinflammation associated with reactive glial activation. Interestingly, Cx43 expression decreased in the absence of Cx30, suggesting complex reciprocal regulatory interactions between these connexins [159]. Conversely, Cx36 displayed the opposite trend in ALS models. Its expression was significantly reduced in both transgenic mice carrying the SOD1 mutation and in patients with ALS. Furthermore, genetic deletion of Cx36 reduced neuronal death induced by overexpression of mutant SOD1 G93A [160].

Additional studies have demonstrated that expression of Cx47, Cx32, and the EAAT2 decreases in the anterior horns of the spinal cord during the progressive and terminal stages of ALS, particularly in oligodendrocytes expressing high levels of mutant SOD1. In contrast, Cx43, Cx30, GFAP, and AQP4 exhibit the opposite pattern of increased expression. These alterations are associated with neuronal loss, axonal degeneration, and enhanced activation of reactive microglia [19].

An interesting aspect of ALS pathogenesis involves the potential neuroprotective effects of hyperinsulinemia. A computational modeling study suggested that insulin may interact with Cx43 and Cx31, binding to the N-terminal domain of monomeric connexins and blocking the open channel of hexameric Cx31 hemichannels. This mechanism may partially explain the protective association observed between type 2 diabetes mellitus and ALS risk [161].

Collectively, the studies reviewed in this section highlight the complex and often dual role of connexins in amyotrophic lateral sclerosis. While upregulation of astrocytic Cx43 and Cx30 is frequently associated with neuroinflammation, excitotoxicity, and motor neuron toxicity, other connexins such as Cx36 and Cx32 show decreased expression in advanced disease stages. Most mechanistic data derive from SOD1-G93A transgenic models and iPSC-derived astrocytes, while findings from human postmortem tissue provide additional clinical relevance. Depending on the isoform, cellular context, and disease stage, connexins can exert either neuroprotective or neurotoxic effects. A comprehensive overview of the experimental models, connexin isoforms, modulation approaches, key results, and corresponding references is systematically summarized in Table 8.

#### 3.3.4. Huntington’s Disease

**Figure 10:**
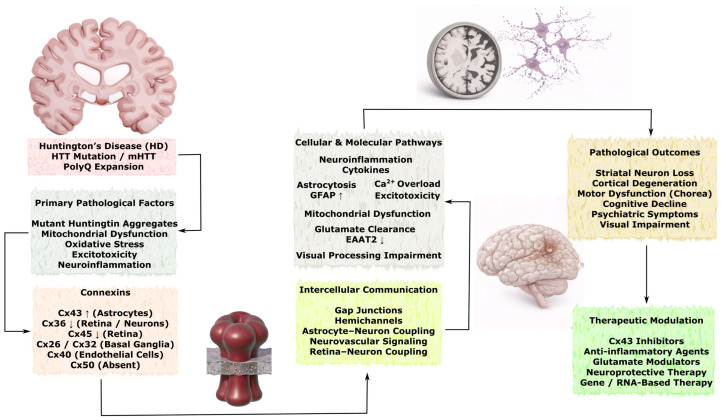
Connexin-Mediated Mechanisms in HD. This schematic diagram summarizes the role of connexins in the molecular and cellular mechanisms underlying HD. Expansion of polyglutamine repeats in the huntingtin gene leads to accumulation of mutant huntingtin (mHTT), which is associated with neuronal dysfunction, mitochondrial impairment, oxidative stress, and neuroinflammation in the striatum, cortex, and retina. These pathological processes are accompanied by dysregulated expression of multiple connexins, particularly increased astrocytic Cx43 and reduced levels of Cx36 and Cx45 in retinal and neuronal networks, as well as altered expression of Cx26, Cx32, and Cx40 in basal ganglia and vascular cells. Altered connexin-mediated GJs and hemichannels may impair astrocyte–neuron communication, glutamate clearance, and neurovascular signaling, thereby contributing to excitotoxic stress and inflammatory responses. Connexin-dependent pathways regulate calcium (Ca^2+^) homeostasis, apoptotic signaling, and synaptic function. These mechanisms contribute to progressive striatal and cortical degeneration, motor impairment, cognitive decline, psychiatric symptoms, and visual dysfunction. Targeting connexins and related signaling pathways may represent a potential strategy for neuroprotection in HD. Arrows indicate direction of change: ↑ increased expression/activity; ↓ decreased expression/activity. Abbreviations: HD, Huntington’s disease; Cx, connexin; Cx43, connexin 43; Cx36, connexin 36; Cx45, connexin 45; Cx26, connexin 26; Cx32, connexin 32; Cx40, connexin 40; Cx50, connexin 50; mHTT, mutant huntingtin; PolyQ, polyglutamine expansion; CN, caudate nucleus; GP, globus pallidus; ATP, adenosine triphosphate; Ca^2+^, calcium ion; ROS, reactive oxygen species; EAAT2, excitatory amino acid transporter 2; GFAP, glial fibrillary acidic protein.

Huntington’s disease (HD) is a severe hereditary neurodegenerative disorder caused by a mutation in the huntingtin (HTT) gene, leading to accumulation of mutant huntingtin protein and progressive neuronal degeneration in the basal ganglia, cortex, and other brain regions, including the retina. The primary pathogenic mechanism involves expansion of polyglutamine repeats in the HTT protein, resulting in protein misfolding, transcriptional dysregulation, mitochondrial dysfunction, and progressive neuronal vulnerability. Alterations in connexin-mediated intercellular communication have been increasingly associated with secondary mechanisms that may influence propagation of neuroinflammatory signaling, astrocyte reactivity, and neuronal network dysfunction in HD. Several connexin isoforms—including Cx43, Cx36, Cx45, Cx26, Cx32, and Cx40—have been implicated in processes related to glial activation, excitotoxic signaling, and neurovascular regulation. The principal Cx-dependent mechanisms of intercellular communication and pathological consequences of HD are schematically illustrated in Figure 10.

Studies in patients with HD have revealed alterations in connexin expression in the caudate nucleus (CN) and globus pallidus (GP). For instance, Cx50 was not detected, whereas Cx40 was localized exclusively in endothelial cells of blood vessels. Cx26 and Cx32 displayed similar distribution patterns in both the CN and GP, showing weak labeling in the CN and more pronounced staining in the GP, without significant deviation from normal expression levels. In contrast, Cx43 expression was markedly increased in the CN, displaying a heterogeneous distribution pattern accompanied by increased reactive astrogliosis. However, in the GP, Cx43 expression remained uniformly distributed within the neuropil, similar to that observed in healthy brain tissue [24].

Studies of the retina in HD mouse models have shown that Cx36 levels were slightly reduced in the outer plexiform layer, correlating with degeneration of photoreceptor terminals. Conversely, Cx45 expression was markedly decreased, and this reduction may be associated with impaired visual processing observed in HD [162]. Additionally, investigations of the midcingulate cortex in patients with HD revealed decreased expression of the EAAT2 accompanied by affective symptoms and Cx43-associated astrogliosis [163].

Collectively, the studies reviewed in this section indicate that alterations in connexin expression in Huntington’s disease are primarily associated with reactive astrogliosis and secondary pathological processes rather than primary disease initiation. Increased Cx43 expression is consistently observed in the caudate nucleus and globus pallidus, while Cx36 and Cx45 show reduced levels in the retina. Most data derive from postmortem human brain tissue and the R6/2 transgenic mouse model. Depending on the isoform and brain region, Cxs may contribute to neuroinflammation, impaired astrocyte–neuron communication, and visual dysfunction. A comprehensive overview of the experimental models, connexin isoforms, modulation approaches, key results, and corresponding references is systematically summarized in Table 9.

### 3.4. Role of Connexins in Psychiatric Disorders

Psychiatric disorders are characterized by complex alterations in neuronal and glial function affecting synaptic transmission, network connectivity, and neuroinflammatory signaling. In contrast to neurodegenerative diseases, psychiatric conditions typically lack a single primary molecular lesion and instead involve multifactorial dysregulation of neurotransmitter systems, stress-response pathways, and neuroglial communication. Increasing evidence suggests that Cx-mediated GJs and hemichannels may influence astrocyte–neuron coupling, inflammatory signaling, and network synchronization in these disorders. However, current data largely indicate that alterations in Cx expression and function represent secondary or modulatory mechanisms associated with glial dysfunction, rather than primary disease-initiating factors. Cx-dependent pathways may contribute to altered neuroplasticity, stress vulnerability, and dysregulated neurotransmission observed across mood and psychotic disorders.

#### 3.4.1. Mood Disorders

##### Depression

**Figure 11:**
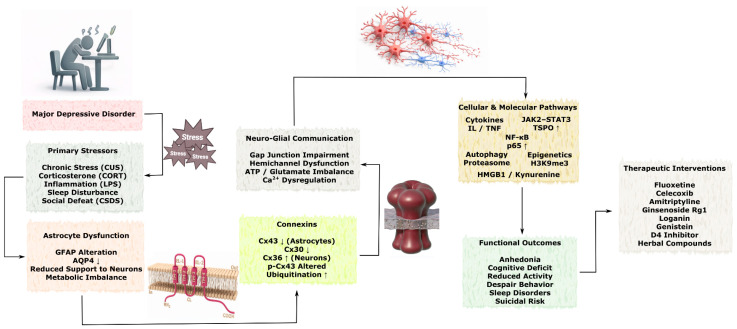
Connexin-Mediated Mechanisms in MDD. This schematic diagram summarizes the role of connexins in the molecular and cellular mechanisms underlying MDD. Chronic stress, glucocorticoid exposure, and inflammatory stimuli induce astrocytic dysfunction in the prefrontal cortex, hippocampus, and orbitofrontal cortex, leading to reduced expression and impaired function of Cx43 and Cx30 accompanied by altered neuronal Cx36 signaling. Disruption of connexin-mediated GJs and hemichannels promotes neuroinflammation, excessive cytokine release, and dysregulated astrocyte–neuron communication. Reduced Cx43 activates JAK2–STAT3 and NF-κB signaling pathways, enhances translocator protein (TSPO) expression, and facilitates ubiquitin–proteasome- and autophagy-mediated degradation of connexins. Epigenetic repression further contributes to long-term astrocytic dysfunction. In parallel, increased neuronal Cx36 activity amplifies inflammatory signaling and excitatory neurotransmission. These processes impair synaptic activity, network connectivity, and cognitive function, resulting in depressive-like behaviors. Pharmacological antidepressants, natural compounds, and selective hemichannel inhibitors restore connexin expression and function, suppress neuroinflammation, and promote behavioral recovery, highlighting connexins as promising therapeutic targets in MDD. Arrows indicate direction of change: ↑ increased expression/activity; ↓ decreased expression/activity. Abbreviations: MDD, major depressive disorder; Cx, connexin; Cx43, connexin 43; Cx30, connexin 30; Cx36, connexin 36; PFC, prefrontal cortex; LPS, lipopolysaccharide; CUS, chronic unpredictable stress; CORT, corticosterone; IL, interleukin; TNF-α, tumor necrosis factor alpha; JAK2, Janus kinase 2; STAT3, signal transducer and activator of transcription 3; NF-κB, nuclear factor kappa B; TSPO, translocator protein; AQP4, aquaporin 4; HMGB1, high-mobility group box 1; CREB, cAMP response element-binding protein; BDNF, brain-derived neurotrophic factor; YAP, Yes-associated protein; GSK-3β, glycogen synthase kinase 3 beta; CBX, carbenoxolone; miR, microRNA.

Major depressive disorder (MDD) is one of the most prevalent and disabling neuropsychiatric conditions, characterized by anhedonia, pessimism, cognitive impairment, and an elevated risk of suicide. Current evidence indicates that the pathogenesis of MDD involves not only neuronal dysfunction but also pronounced alterations in astrocyte function, particularly disturbances in intercellular communication mediated by GJs and hemichannels. A key molecular component of these processes is the astrocytic connexin Cx43 [164], and to a lesser extent Cx30 and the neuronal connexin Cx36. Neuroinflammation plays a major role in exacerbating these abnormalities, forming a bidirectional pathological relationship between inflammatory signaling and connexin dysfunction [25]. The principal Cx-dependent mechanisms of intercellular communication and pathological consequences of depression are illustrated in Figure 11.

One of the most consistently observed alterations in depression is the reduction in expression and functional activity of astrocytic Cx43 in the prefrontal cortex (PFC), hippocampus, and orbitofrontal cortex. Experimental models involving pharmacological blockade of Cx43—for example, with the peptides Gap27 [165], Gap26, or carbenoxolone [166] —or conditional knockout of Cx43 in the PFC induce depressive-like behaviors, including reduced sucrose preference, decreased spontaneous locomotor activity, and increased immobility in the forced swim test and tail suspension test. These behavioral alterations are accompanied by pronounced activation of peripheral and central inflammatory responses, reflected by elevated levels of pro-inflammatory cytokines (IL-1β, IL-6, TNF-α, IL-2, IL-10, and IL-18), indicating a strong association between Cx43 dysfunction and inflammatory signaling pathways [165].

Neuroinflammation induced by systemic LPS administration also reduces Cx43 expression and GJ permeability in the PFC, thereby aggravating depressive symptoms. These findings support the existence of a “vicious cycle”, in which inflammation suppresses Cx43 function, while GJ dysfunction further amplifies inflammatory responses [165]. At the molecular level, reduced Cx43 expression activates the JAK2–STAT3 signaling pathway, leading to increased expression of the mitochondrial transporter protein TSPO, a key biomarker of neuroinflammation, and the development of depressive-like behavior [167]. Additionally, disruption of GJ function enhances activation of NF-κB (phosphorylation of p65), whereas inhibition of this pathway restores Cx43 phosphorylation and improves intercellular communication [168].

An important mechanism in depression pathogenesis also involves regulation of Cx43 degradation. In models of CUS [169] and CORT exposure [170], accelerated degradation of Cx43 occurs through both ubiquitin–proteasome and autophagy–lysosomal pathways, which is accompanied by enhanced neuroinflammation [169,170]. Pharmacological inhibition of Cx43 ubiquitination, for example using MG132, attenuates inflammatory responses and depressive-like behavior [169]. Epigenetic mechanisms also contribute to the regulation of Cx43 expression. In depressed suicide victims, enrichment of the repressive histone mark H3K9me3 has been detected in the promoter regions of Cx43 and Cx30 genes in the PFC, suggesting long-term repression of astrocytic connexins [171].

In addition to Cx43, astrocytic Cx30 also plays an important role in region-specific alterations associated with depression. In the chronic social defeat stress (CSDS) model, expression of Cx30 and Cx43 decreases in the medial PFC and hippocampus, accompanied by reduced neuronal activity and depressive-like behavior. Overexpression of these connexins in these regions restores neuronal activity and behavioral outcomes, whereas their suppression in otherwise healthy animals induces depressive phenotypes [26]. Conversely, increased Cx30 expression in the cerebellum has been observed in depressed suicide victims, highlighting the strong regional specificity of astrocytic dysfunction [171].

In contrast to astrocytic connexins, the neuronal connexin Cx36 exhibits the opposite trend in depression. In the hippocampus under chronic stress conditions, Cx36 expression increases, enhancing release of HMGB1, elevating pro-inflammatory cytokine levels, and increasing neuronal excitability. This also disrupts kynurenine metabolism, leading to enhanced glutamatergic transmission. Pharmacological inhibition of Cx36 reduces inflammation and attenuates depressive-like behavior, suggesting a pathogenic role for this connexin [172].

The systemic nature of astrocytic dysfunction in depression is further supported by clinical data. In patients with chronic insomnia disorder, frequently comorbid with depression, decreased serum levels of Cx43, Cx30, and AQP4 have been detected, correlating with impaired cognitive function and reduced slow-wave sleep [173]. Postmortem studies also demonstrate more than a 60% reduction in Cx43 levels in the orbitofrontal cortex of patients with depression and alcoholism. This reduction is accompanied by decreased area and size of Cx43-immunoreactive puncta, indicating impaired astrocytic communication through GJs and hemichannels [174].

Both conventional antidepressants and plant-derived compounds are capable of modulating connexin function, supporting their therapeutic potential. Fluoxetine normalizes the increased phosphorylation of Cx43 in the hippocampus induced by CORT [175] and enhances antidepressant effects under conditions of Cx43 knockdown [176], likely through cAMP-dependent mechanisms [177]. Celecoxib, a COX-2 inhibitor, suppresses NF-κB activation, normalizes Cx43 phosphorylation, improves GJ function in the PFC, and restores connectivity within the default mode network [168]. In the context of neuropathic pain, the antidepressant effect of amitriptyline correlates with decreased Cx43 expression in the hippocampus, suggesting that Cx43 may serve as a biomarker of therapeutic response [178].

Plant-derived compounds demonstrate particularly strong effects on astrocytic connexins. Ginsenoside Rg1 enhances Cx43 biosynthesis, reduces its degradation, suppresses ubiquitination, and limits nuclear translocation of YAP, thereby attenuating neuroinflammation and restoring functional connectivity in the PFC and hippocampus [166,169,170,179,180,181]. Alkaloids derived from Mahonia fortunei increase Cx43 expression through suppression of miR-205, followed by activation of the CREB/BDNF signaling pathway, leading to improvements in behavior and neurotransmission [182,183]. Loganin increases Cx43 expression, reduces its phosphorylation, and activates the GSK-3β/β-catenin signaling pathway, demonstrating synergistic antidepressant effects with fluoxetine and a faster onset of therapeutic action [184]. Genistein suppresses miR-221/222, thereby increasing Cx43 expression and alleviating depressive behavior [185], whereas hypericin normalizes Cx43 phosphorylation and the ultrastructure of GJs, improving intercellular communication [186]. Korean red ginseng also increases Cx43 expression and improves astrocytic GJ function in the PFC, contributing to attenuation of depressive symptoms [187].

Among emerging therapeutic approaches, the selective hemichannel inhibitor D4 has shown promising effects by reducing neuroinflammation, normalizing neuronal activity in the hippocampus, entorhinal cortex, and lateral septum, and eliminating depressive-like behavior in LPS- and stress-induced depression models [188]. Additionally, overexpression of Cx43 in the hippocampus during early-life stress alleviates cognitive deficits and astrocyte dysfunction, further highlighting the neuroprotective role of this connexin [189].

Overall, astrocytic connexins—particularly Cx43—occupy a central position in the pathogenesis of MDD, linking GJ and hemichannel dysfunction with neuroinflammation, impaired neuronal activity, and cognitive disturbances. Modulation of connexin expression and function through antidepressants, plant-derived compounds, and selective hemichannel inhibitors represents a promising direction for the development of novel personalized therapeutic strategies for depression.

Collectively, the studies reviewed in this section demonstrate the central role of astrocytic Cx43 (and to a lesser extent Cx30) dysfunction in the pathogenesis of major depressive disorder. Reduced expression and impaired function of Cx43 in the prefrontal cortex, hippocampus, and orbitofrontal cortex are consistently observed across various stress models and human postmortem studies. This dysfunction is tightly linked to neuroinflammation, impaired glutamate homeostasis, and depressive-like behaviors. In contrast, neuronal Cx36 often shows increased activity under chronic stress. Both conventional antidepressants and plant-derived compounds restore Cx43 expression and gap junction function, producing significant antidepressant effects. A comprehensive overview of the experimental models, connexin isoforms, modulation approaches, key results, and corresponding references is systematically summarized in Table 10.

##### Bipolar Disorder

**Figure 12:**
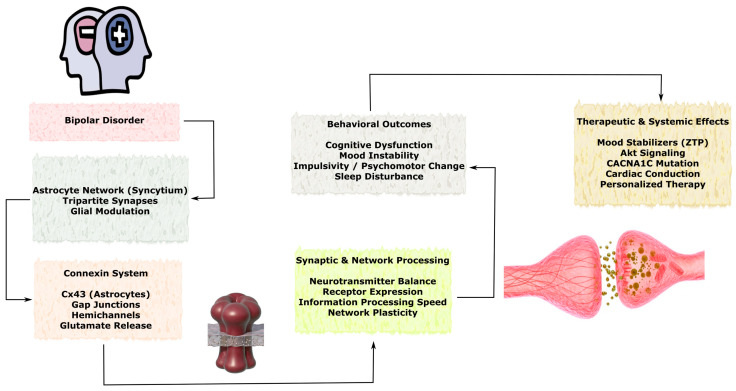
Connexin-Mediated Mechanisms in BD. This schematic diagram illustrates the role of astrocytic connexins in the pathophysiology of BD. Dysregulation of Cx43 in the astrocytic syncytium alters gap junction and hemichannel function, leading to impaired modulation of tripartite synapses and disrupted astrocyte–neuron communication. During depressive episodes, reduced Cx43 expression weakens astrocytic coupling, enhances receptor expression, slows synaptic information processing, and promotes neurotransmitter deficiency. In contrast, manic episodes are characterized by increased Cx43 expression, enhanced interastrocytic communication, reduced receptor density, accelerated synaptic processing, and excessive neurotransmitter release. Microglial activation and inflammatory signaling further modulate astrocytic receptors and connexin function, contributing to mood instability. Pharmacological modulation by mood stabilizers, including zotepine, regulates Cx43-dependent glutamatergic transmission via Akt signaling pathways. Genetic variants, such as mutations in CACNA1C, affect Cx43-mediated GJs in both neural and cardiac tissues, linking BD to altered cardiac conduction and increased arrhythmogenic risk. These interconnected mechanisms underlie cyclic mood switching, cognitive dysfunction, and systemic complications in BD. Abbreviations: BD, bipolar disorder; Cx, connexin; Cx43, connexin 43; GJ, gap junction; HC, hemichannel; ZTP, zotepine; Akt, protein kinase B; CACNA1C, calcium voltage-gated channel subunit alpha1 C; PFC, prefrontal cortex; IL, interleukin; TNF-α, tumor necrosis factor alpha; ATP, adenosine triphosphate; QT, QT interval on electrocardiogram.

Bipolar disorder (BD) is a chronic neuropsychiatric condition characterized by alternating manic and depressive episodes, frequently accompanied by cognitive impairment. Astrocytes, which regulate synaptic activity through tripartite synapses, play a critical role in the pathophysiology of BD, particularly through alterations in the expression of connexins such as Cx43, which form both gap junctions and hemichannels. The principal Cx-dependent mechanisms of intercellular communication and pathological consequences of BD are illustrated in Figure 12.

A proposed pathophysiological model of BD is based on dysregulation of Cx43 expression within the astrocytic syncytium, affecting tripartite synaptic signaling. During depressive episodes, reduced Cx43 expression disrupts astrocytic communication, triggering compensatory upregulation of astrocytic receptors. This slows synaptic information processing and leads to deficits in neurotransmitter availability. Consequently, prolonged activation of behavioral generation systems occurs, impairing the selection of adaptive behavioral responses. In contrast, during manic states, increased Cx43 expression enhances astrocytic communication, resulting in decreased receptor expression and accelerated synaptic processing. This leads to excessive neurotransmitter release and rapid switching between behavioral states. Dysregulation of gliotransmitters, particularly L-glutamate, together with dysfunction of GJs and hemichannels, further exacerbates cognitive deficits characteristic of both phases of BD. The transition between depressive and manic states may represent an attempt of the neural system to compensate for one pathological state by shifting to another, mediated largely by astrocytic dysfunction [27,190].

Experimental studies investigating the effects of ZTP, an atypical antipsychotic with mood-stabilizing properties, in primary cortical astrocytes demonstrated that chronic administration of therapeutic doses of ZTP increases Cx43 expression in the plasma membrane and enhances L-glutamate release through activated hemichannels, an effect suppressed by Akt inhibitors. Supratherapeutic doses of ZTP further amplify these effects, including the emergence of proconvulsant activity, suggesting that the mood-stabilizing action of ZTP depends on activation of astroglial glutamatergic transmission mediated by Cx43. These findings highlight the role of Cx43 in modulating neurotransmission in BD and suggest potential risks associated with high-dose antipsychotic therapy [191].

A genetic risk factor associated with BD involves mutations in the CACNA1C gene, which affect not only neuropsychiatric phenotypes but also cardiac electrical conduction. In cardiomyocytes derived from iPSCs of patients carrying CACNA1C mutations, conduction velocity of electrical impulses was reduced due to impaired intercellular communication through Cx43-containing GJs. Gene therapy aimed at restoring Cx43 expression improved conduction velocity and protected against thioridazine-induced QT interval prolongation, indicating a higher proarrhythmic risk in BD patients receiving psychiatric medications. These findings emphasize the systemic significance of Cx43 in BD, including its role in cardiovascular physiology, and highlight the importance of personalized therapeutic strategies [192].

Microglia also contribute to BD pathophysiology by amplifying neuroinflammatory responses and disrupting glia–neuron interactions. Increased expression of astrocytic receptors and GJs during depressive states may be associated with microglial activation, whereas their reduction during manic phases contributes to neuronal hyperexcitability. Genetic, epigenetic, and chronobiological factors, including stress exposure, further destabilize the astrocytic syncytium and exacerbate imbalances in neural information processing. Within the framework of the tripartite synapse model, assessment of Cx43 expression levels and astrocytic receptor activity across different phases of BD may provide valuable insights for improving diagnostic precision and developing more effective therapeutic approaches [190].

Collectively, the studies reviewed in this section demonstrate the complex and phase-dependent role of Cx43 in bipolar disorder. In depressive episodes, reduced Cx43 impairs astrocytic coupling and glutamatergic transmission, whereas in manic phases Cx43 upregulation enhances intercellular communication. Mood stabilizers such as zotepine restore Cx43 function via the Akt pathway, while genetic CACNA1C mutations impair Cx43 GJs, linking BD to increased cardiovascular risk. Most evidence derives from in vitro astrocyte cultures and iPSC-cardiomyocyte models. A comprehensive overview of the experimental models, connexin isoforms, modulation approaches, key results, and corresponding references is systematically summarized in Table 11.

##### Suicidal Behavior

Suicidal behavior represents a complex and multifactorial clinical phenotype observed across multiple psychiatric disorders, including major depressive disorder and bipolar disorder. Converging evidence suggests that neuroinflammatory processes, impaired neuroplasticity, and glial dysfunction may contribute to increased vulnerability to suicidal behavior. In this context, alterations in Cx-mediated intercellular communication have been reported, particularly in astrocytes and oligodendrocytes.

Postmortem studies of the dorsolateral prefrontal cortex from French-Canadian males who died by suicide revealed decreased expression of Cx30 and Cx43. Analysis of transcription factors identified Sox9 as a regulator of Cx30 expression, suggesting molecular mechanisms underlying astrocytic dysfunction in suicide. These alterations have been replicated in larger cohorts, including datasets from the Stanley Foundation, supporting the association between reduced astrocytic connexin expression and suicidal behavior [28].

In the anterior cingulate cortex of depressed suicide victims, decreased expression of Cx30—localized to oligodendrocytes and myelinated fibers—has also been observed, particularly in deep cortical layers in males. This reduction is accompanied by decreased expression of oligodendrocyte-specific connexins Cx32 and Cx47, as well as lower levels of proteins required for the formation and maintenance of functional gap junctions. These findings indicate impaired heterotypic coupling between astrocytes and oligodendrocytes, which may affect myelination and neuronal activity, thereby contributing to the psychopathology of mood disorders and suicidal behavior [20].

Collectively, the studies reviewed in this section demonstrate that reduced expression of astrocytic and oligodendrocytic Cx30, Cx43, Cx32, and Cx47 is a consistent feature of suicidal behavior in the context of mood disorders. These alterations, observed in the dorsolateral prefrontal cortex and anterior cingulate cortex, are associated with impaired astrocyte–oligodendrocyte coupling, disrupted myelination, and enhanced neuroinflammation. Most evidence derives from postmortem human brain analyses. A comprehensive overview of the experimental models, connexin isoforms, modulation approaches, key results, and corresponding references is systematically summarized in Table 12.

#### 3.4.2. Schizophrenia

**Figure 13:**
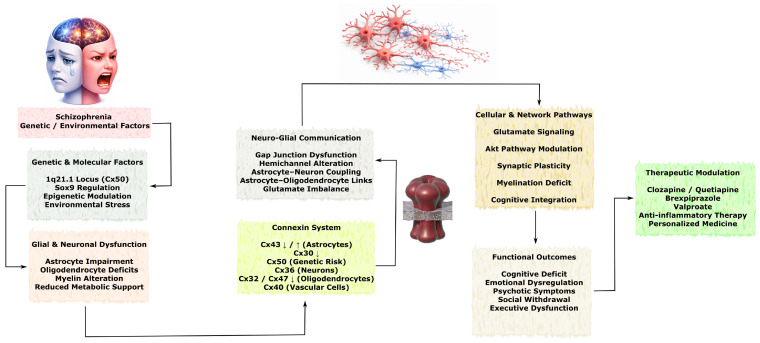
Connexin-Mediated Mechanisms in Schizophrenia. This schematic diagram summarizes the role of connexins in the molecular and cellular mechanisms underlying schizophrenia. Genetic vulnerability, including variants in the 1q21.1 locus and regulatory pathways involving Sox9, interacts with environmental stressors to induce astrocytic, oligodendrocytic, and neuronal dysfunction. These processes lead to dysregulated expression of multiple connexins, particularly astrocytic Cx43 and Cx30, neuronal Cx36, within broader panglial connexin networks that may also involve oligodendrocytic connexins, and vascular Cx40, as well as genetic susceptibility associated with Cx50. Altered connexin-mediated gap junctions and hemichannels disrupt astrocyte–neuron and astrocyte–oligodendrocyte coupling, impair glutamatergic transmission, and reduce network integration. Connexin-dependent modulation of Akt signaling and tripartite synapse function contributes to cognitive deficits, emotional dysregulation, and psychotic symptoms. Reduced astrocytic connexin expression in prefrontal and cingulate cortices is associated with impaired functional connectivity and increased vulnerability to suicidal behavior. Pharmacological modulation by atypical antipsychotics and mood stabilizers partially restores connexin-dependent signaling, highlighting connexins as potential biomarkers and therapeutic targets in schizophrenia and suicide-related psychopathology. Arrows indicate direction of change: ↑ increased expression/activity; ↓ decreased expression/activity. Abbreviations: Cx, connexin; Cx43, connexin 43; Cx30, connexin 30; Cx36, connexin 36; Cx32, connexin 32; Cx47, connexin 47; Cx40, connexin 40; Cx50, connexin 50; Akt, protein kinase B; Sox9, SRY-box transcription factor 9; PFC, prefrontal cortex; ACC, anterior cingulate cortex.

Schizophrenia is a complex neuropsychiatric disorder in which disturbances of neuronal and glial communication have been increasingly implicated, including alterations in GJ signaling mediated by connexins [193]. Connexins such as Cx40, Cx50, Cx36, Cx30, and Cx43 facilitate intercellular exchange of ions and signaling molecules, including glutamate, thereby influencing synaptic activity and cognitive processes. Genetic, molecular, and neurobiological studies highlight associations between connexin-related pathways and glial dysfunction observed in schizophrenia, suggesting that these alterations may contribute to impaired intercellular communication rather than acting as primary disease-initiating mechanisms. Based on data from genetic association studies, preclinical models, and postmortem brain analyses, several key aspects of connexin involvement in these conditions can be identified, including their relationship with glial dysfunction and their contribution to psychopathology. The principal Cx-dependent mechanisms of intercellular communication and connexin-associated alterations described in schizophrenia are schematically illustrated in Figure 13.

Schizophrenia, characterized by generalized cognitive impairment, has been linked to abnormalities in chromosomal regions including 1q21.1, where the connexin genes Cx40 and Cx50 are located. Studies in European populations demonstrated that the Cx50 haplotype (rs989192–rs4950495, AC) occurs more frequently in patients with schizophrenia compared with control groups, and family-based analyses confirmed its increased transmission to probands with schizophrenia. These findings indicate a genetic susceptibility associated with Cx50, whereas Cx40 showed no significant association with the disorder [194]. In contrast to Cx50, Cx36, which is predominantly expressed in neurons of the brain and retina and is located on chromosome 15q14—a region linked to catatonic schizophrenia—did not exhibit mutations that segregate with the disorder in families affected by this subtype. These results exclude Cx36 as a primary causal gene for catatonic schizophrenia despite its positional relevance [195]. At the functional level, loss of glial GJ function—particularly involving Cx30 and Cx43—has been proposed as a potential contributor to impaired coordination of neuronal activity and information processing. Such disruption may underlie severe cognitive deficits observed in schizophrenia. Glial GJs participate in monitoring neuronal activation and forming GJ plaques that support information processing; therefore, their dysfunction may lead to impaired discrimination of neurotransmitter signaling and synaptic properties, thereby exacerbating cognitive impairment [196].

Tripartite synaptic transmission involving astrocytes also plays an important role in schizophrenia through regulation of glutamatergic signaling. Preclinical studies have shown that atypical antipsychotics and mood stabilizers—including clozapine, quetiapine, and brexpiprazole—enhance astroglial L-glutamate release via activation of Cx43 hemichannels. Subchronic administration of these drugs increases Cx43 expression in astrocytic membranes, particularly when combined with valproate. The mechanisms of activation differ: clozapine and quetiapine act through the Akt signaling pathway, whereas brexpiprazole operates independently of Akt. These findings suggest that enhanced astroglial glutamatergic transmission mediated by Cx43 may constitute part of the therapeutic mechanism of antipsychotic drugs in schizophrenia and affective disorders [197].

Regional specificity of connexin alterations further supports their role in cognitive and emotional dysfunction. The hypothesis that glial gap junction dysfunction contributes to cognitive deficits in schizophrenia emphasizes their role in differentiating neurotransmitter signals and highlights the need for further investigation in other brain regions, such as the hippocampus and thalamus [196]. Finally, intracellular signaling pathways such as Akt may participate in regulation of astrocytic connexin expression, reflecting broader alterations in glial signaling networks associated with antipsychotic treatment responses [197].

Collectively, these findings indicate that alterations in connexin expression and function contribute to the pathophysiology of schizophrenia through impaired glial-neuronal communication and disrupted synaptic integration. A comprehensive overview of the experimental models, connexin isoforms, modulation approaches, key results, and corresponding references is systematically summarized in Table 13.

## 4. Role of Connexins in Epilepsy

**Figure 14:**
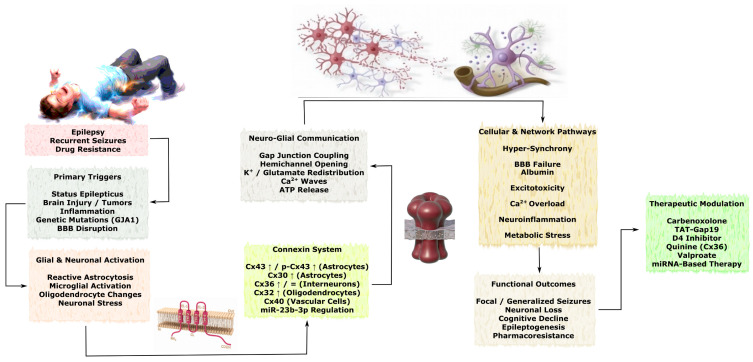
Connexin-Mediated Mechanisms in Epilepsy. This schematic diagram summarizes the role of connexins in the molecular and cellular mechanisms underlying epilepsy. Genetic susceptibility, brain injury, inflammation, and BBB disruption trigger activation of astrocytes, microglia, and oligodendrocytes, leading to dysregulated expression and post-translational modification of multiple connexins, particularly astrocytic Cx43 and Cx30, neuronal Cx36 and oligodendrocytic Cx32. Altered connexin-mediated GJs and hemichannels promote abnormal redistribution of potassium (K^+^) and glutamate, excessive calcium (Ca^2+^) signaling, ATP release, and astrocytic calcium wave propagation, thereby enhancing neuronal synchronization and network hyperexcitability. Increased activity of Cx43 hemichannels contributes to BBB dysfunction, albumin extravasation, and neuroinflammation, further facilitating epileptogenesis. Connexin-dependent pathways regulate excitotoxicity, metabolic stress, and neuronal survival, promoting seizure generation and disease progression. Pharmacological and genetic modulation of connexins, including hemichannel inhibitors and microRNA-based approaches, represents a promising therapeutic strategy for drug-resistant epilepsy. Arrows indicate direction of change: ↑ increased expression/activity. Abbreviations: Cx, connexin; Cx43, connexin 43; Cx30, connexin 30; Cx36, connexin 36; Cx32, connexin 32; Cx40, connexin 40; BBB, blood–brain barrier; ATP, adenosine triphosphate; Ca^2+^, calcium ion; K^+^, potassium ion; GJA1, gap junction protein alpha 1; miR, microRNA; ZO-1, zonula occludens-1; EEG, electroencephalogram.

Epilepsy, one of the most prevalent neurological disorders, is characterized by recurrent seizures caused by abnormal synchronous neuronal activity and remains a major clinical challenge due to pharmacoresistance in approximately one-third of patients treated with conventional anticonvulsant drugs targeting neuronal mechanisms. In recent years, research attention has shifted toward non-neuronal targets, particularly astrocytic proteins. Among them, connexins—including Cx43, Cx30, Cx36, Cx32, and Cx40—play an important role in the pathogenesis of epilepsy. These proteins form GJs and hemichannels, enabling intercellular and extracellular communication that influences neuronal excitability, neuroinflammation, and the integrity of the BBB. Evidence from experimental models, studies of human tissue, and pharmacological investigations highlights several key aspects of connexin involvement in epileptogenesis, their impact on seizure activity, and their potential as targets for novel therapeutic strategies [198]. The principal Cx-dependent mechanisms of intercellular communication and pathological consequences of epilepsy are summarized in Figure 14.

The astrocytic connexin Cx43, widely expressed in the central nervous system, represents a major component of GJs and hemichannels that mediate the exchange of ions, glutamate, and other signaling molecules. In epileptic human tissue and experimental models such as the pilocarpine model of temporal lobe epilepsy, alterations in Cx43 mRNA levels, protein expression, phosphorylation status, and subcellular localization have been observed. For instance, in the hippocampus of rats following pilocarpine-induced status epilepticus, Cx43 expression is markedly increased during both latent and chronic stages, particularly in the CA1, CA3, and dentate gyrus regions, correlating with reactive astrogliosis and neuronal loss [199]. Similarly, in human temporal lobe epilepsy with hippocampal sclerosis, Cx43 redistributes to perivascular astrocytic endfeet, accompanied by increased phosphorylation that alters channel permeability and is associated with BBB disruption, as evidenced by albumin extravasation [200]. In kainic acid–induced models, Cx43 is also overexpressed around blood vessels, further increasing BBB permeability and promoting seizure progression [201]. Mutations in the GJA1 gene, which encodes Cx43, are associated with neurological symptoms including seizures in approximately 30% of patients with oculodentodigital dysplasia, highlighting the genetic contribution of Cx43 to epileptogenesis [202].

Another astrocytic connexin, Cx30, also contributes significantly to epilepsy pathophysiology. In kainate-induced seizure models, Cx30 expression increases and exacerbates behavioral seizure severity by regulating astrocytic glutamate clearance, independently of direct biochemical coupling through GJs [203]. Mice with double knockout of Cx30/Cx43 exhibit increased susceptibility to epileptiform events in brain slices due to impaired redistribution of potassium and glutamate, as well as a higher frequency of spontaneous generalized seizures in chronic epilepsy models. However, Cx30/Cx43 GJs demonstrate dual effects, transporting metabolic substrates to neurons with high energy demands while simultaneously generating astrocytic calcium waves that promote hypersynchronization and proconvulsant activity [202]. In low-grade epilepsy-associated tumors, Cx30 is expressed in the astroglial component, and its high expression in peritumoral reactive astrocytes suggests a role in seizure generation [204].

The neuronal connexin Cx36, predominantly localized between GABAergic interneurons, is also implicated in epileptogenesis. In an in vivo 4-aminopyridine model, blockade of Cx36 with quinine reduces the overall seizure burden by shortening seizure duration, although the number of seizures may increase, indicating its role in neuronal network synchronization [14]. In the pilocarpine model, Cx36 expression remains relatively stable, in contrast to the marked overexpression of Cx43 during focal seizure stages, highlighting distinct contributions of neuronal and glial connexins [205]. In the 4-aminopyridine model, Cx36 expression increases in the hippocampus, particularly at later stages of seizures, potentially contributing to sustained epileptiform activity [206]. Pharmacological inhibition of Cx36 is therefore considered a promising strategy to protect neurons from degeneration and pathological synchronization during seizures [207].

The oligodendrocyte-specific connexin Cx32 also contributes to epilepsy, particularly in brain tumors associated with chronic pharmacoresistant epilepsy. In glioneuronal tumors and oligodendrogliomas, Cx32 is expressed in both neuronal and oligodendrocytic components, and its high immunoreactivity correlates with seizure activity [204]. In the 4-aminopyridine model, Cx32 is overexpressed in the hippocampus, especially in the dentate gyrus, where it is associated with increased oligodendrocyte density and may contribute to early seizure stages [206]. In human epileptic foci, Cx32 expression is significantly higher than in control tissue, suggesting its involvement in pathological synchronization [208].

Pharmacological modulation of connexins has demonstrated significant therapeutic potential. Carbenoxolone, a non-selective gap junction blocker, prevents epileptiform events in low-magnesium in vitro models and improves seizure outcomes in vivo. However, its effects depend on the epilepsy type: it alleviates seizures in temporal lobe epilepsy models but exacerbates them in absence epilepsy [209,210]. In kainic acid models, carbenoxolone reduces the frequency and duration of spontaneous seizures, decreases expression of phosphorylated Cx43 in the hippocampus, and improves the microstructure of the CA1 region [210]. The selective Cx43 hemichannel inhibitor TAT-Gap19 reduces BBB permeability and seizure activity in electroencephalographic recordings in temporal epilepsy models, emphasizing the critical role of hemichannels in epileptogenesis [201]. A novel compound D4, which inhibits hemichannels but not GJs, effectively reduces neuroinflammation, restores synaptic inhibition, and improves survival in the pilocarpine model, representing a promising therapeutic strategy for epilepsy with a strong inflammatory component [211]. Interestingly, valproate, a classical anticonvulsant, unexpectedly increases the activity of Cx43 and Panx1 hemichannels, potentially enhancing glial reactivity, although the clinical relevance of this effect remains unclear [212].

Molecular mechanisms underlying connexin dysfunction include post-translational modifications and microRNA-mediated regulation. In the lithium–pilocarpine model, overexpression of miR-23b-3p reduces Cx43 expression in the hippocampus, decreasing pathological high-frequency oscillations and seizure severity while protecting neurons from necrosis [213]. In human samples of focal cortical dysplasia type IIB, Cx43 forms large aggregates around balloon cells, and elevated Cx43 mRNA levels are observed in approximately 25% of cases of cryptogenic epilepsy, indicating abnormal tissue organization and altered network properties [214]. Reactive astrogliosis accompanied by increased expression of Cx43 and GFAP is particularly prominent in temporal epilepsy and may contribute to generalized seizures through enhanced astrocytic communication [215].

Regional specificity of connexin alterations further underscores their role in epileptogenesis. In the hippocampus, Cx43 [199] and Cx30 [203] promote hypersynchronization, whereas Cx36 regulates GABAergic transmission [205]. In the neocortex, connexins associated with oligodendrocytes and astrocytes, particularly Cx32 and Cx43, contribute to early seizure stages, while Cx36 appears to influence later phases [206]. BBB disruption mediated by Cx43 hemichannels represents a critical factor in temporal lobe epilepsy, supported by evidence of albumin extravasation and reduced expression of the tight-junction protein ZO-1 [201]. Inflammatory processes mediated by glial hemichannels further amplify epileptogenesis, making them attractive targets for therapeutic intervention [211].

Collectively, the studies reviewed in this section demonstrate the complex and often dual role of connexins in epilepsy. While most data derive from pilocarpine, kainate, and 4-aminopyridine models of temporal lobe epilepsy, findings from human tissue and pharmacological studies highlight both shared and distinct connexin-mediated pathways. Depending on the isoform, cellular context, and disease stage, connexins can exert either proconvulsant or anticonvulsant effects. A comprehensive overview of the experimental models, connexin isoforms, modulation approaches, key results, and corresponding references is systematically summarized in Table 14.

## 5. Discussion

Cxs constitute a family of transmembrane proteins that form GJs and hemichannels, enabling direct intercellular and extracellular communication in the CNS. The findings of the present review demonstrate that Cxs—particularly Cx43, Cx30, Cx36, Cx32, Cx40, Cx45, and Cx37—occupy a central position in the pathogenesis of a wide spectrum of neurological conditions, ranging from acute cerebrovascular events such as stroke, TBI, and SCI to chronic neurodegenerative and psychiatric disorders. Their roles are highly context-dependent, spatiotemporally regulated, and cell-type specific, which explains the observed duality of their effects, varying from neuroprotection to neurotoxicity. Taken together, these observations indicate that connexins should be considered dynamic regulators of neuroglial homeostasis rather than purely pathological mediators, as their functional effects depend on disease stage, cellular context, and balance between physiological intercellular coupling and pathological channel activation.

Importantly, a growing body of evidence indicates that therapeutic strategies targeting Cxs should not be limited to the inhibition of connexin channels alone. Instead, they should emphasize selective modulation, normalization, or phase-dependent restoration of the physiological balance of Cx-mediated intercellular communication. Many pharmacological agents preferentially target hemichannels while largely preserving the beneficial functions of GJs; others modulate Cx expression, phosphorylation status, subcellular localization, or protein–protein interactions rather than causing non-specific blockade. In certain diseases and disease stages, enhancement or normalization of specific Cx-mediated pathways may even be therapeutically advantageous.

The most extensively studied and clinically relevant connexin is Cx43, the principal astrocytic connexin. In ischemic stroke models (MCAO, OGD/R, pMCAO), Cx43 demonstrates dynamic expression characterized by early upregulation in the peri-infarct region, subsequent reduction in the infarct core, and phase-dependent changes in functional profile [37,38]. Hyperactivation of Cx43 hemichannels induces massive release of glutamate and ATP, propagation of Ca^2+^ waves [69], and activation of TLR4/NF-κB and JAK2/STAT3 signaling pathways [7,8,9], thereby amplifying neuroinflammation, apoptosis, and necroptosis. Selective hemichannel inhibitors consistently demonstrate neuroprotective effects, including reductions in infarct volume, edema, BBB permeability, and cognitive–motor deficits [6,7,44,67]. However, many therapeutic approaches do not aim for complete suppression of connexin signaling but rather achieve preferential targeting of hemichannels while preserving protective GJ coupling, highlighting the importance of selective functional modulation rather than global inhibition. In contrast, complete Cx43 knockout or truncation of its C-terminal domain exacerbates injury, highlighting the protective role of intact GJs in maintaining ionic and metabolic homeostasis [40,41,42,43,48]. It should be noted that constitutive or astrocyte-specific Cx43 knockout simultaneously eliminates both GJs and hemichannels, making it difficult to distinguish the contribution of each. Similarly, non-selective GJ blockers such as carbenoxolone also inhibit hemichannels. Moreover, constitutive knockout models may trigger developmental compensatory mechanisms that significantly alter the observed phenotype and complicate data interpretation. Conditional or inducible knockout approaches are therefore preferable for more precise functional analysis.

These findings emphasize that preservation of physiological GJ communication may be as important as inhibition of pathological hemichannel opening, thereby supporting a paradigm of selective functional modulation rather than non-specific or complete connexin suppression. Cx-mediated signaling participates not only in the propagation of injury but also in protective mechanisms that limit tissue damage through spatial buffering of ions, redistribution of metabolites, and coordinated glial responses. Preservation of these beneficial functions appears essential for optimal recovery after acute CNS injury.

A similar duality is observed in TBI and SCI, where Cx43 functions as a central regulator of both pathological and reparative processes depending on the temporal phase, injury type, subcellular localization, and post-translational modifications. In TBI models—including FPI [71], CCI [73], and free-fall injury models [74]—Cx43 expression markedly increases within the first hours after trauma, peaking at 6–24 h, particularly in the hippocampus and cortex. Elevated immunoreactivity of phosphorylated p-Cx43 is observed in astrocytes surrounding pyramidal neurons of the CA3 region [71]. This overexpression closely correlates with reactive astrogliosis, brain edema, increased tissue water content, and ATP release through activated hemichannels, thereby intensifying inflammatory cascades and excitotoxicity [73,82]. Phosphorylation of Cx43 at serine-368, mediated by ERK1/2, enhances hemichannel and GJ conductivity, facilitating Ca^2+^ wave propagation, oxidative stress, and seizure susceptibility induced by agents such as pentylenetetrazole [88].

Nevertheless, Cx43 may also exert neuroprotective functions during later stages or under specific conditions. It regulates proliferation of neural stem/progenitor cells (NSPCs) in the subgranular zone of the dentate gyrus, where it localizes to vimentin-positive cells [81]. Additionally, Cx43 participates in mitochondrial transfer from astrocytes to injured neurons through the alternative isoform GJA1-20k, which reduces apoptosis, stabilizes mitochondrial function, and supports neuronal regeneration [5]. Cx43-dependent mechanisms of autophagy and mitophagy demonstrate bidirectional regulation: levels of p-Cx43 and LC3-II peak approximately 6 h after CCI in the hippocampus. Inhibition of p-Cx43 suppresses autophagy, whereas autophagy blockade results in accumulation of cytotoxic p-Cx43. Conversely, activation of autophagy promotes internalization of GJs into neuronal cytoplasm [74,75]. Therapeutic interventions such as BMSC therapy suppress Cx43, Beclin-1, and LC3 expression, thereby reducing autophagy in the hippocampus [83], whereas therapeutic hypothermia [85] and remazolam [91] normalize Cx43 levels, reduce edema and ROS production, and attenuate reactive glial activation.

Selective inhibition of Cx43 hemichannels using tools such as Peptide5, Gap27, AS-ODN, or the chimeric antibody MHC1 consistently reduces secondary injury, ferroptosis, autophagy, edema, apoptosis, and neuropathic pain in CCI and juvenile TBI models. For instance, miR-302 suppresses ERK1/2-mediated phosphorylation of Cx43, thereby reducing apoptosis and cognitive impairment [79], while GJ blockers (carbenoxolone, oxidized ATP) or autophagy inhibitors (3-methyladenine) restore LTP and cognitive function by decreasing Beclin-1 and increasing GLT-1 expression [84]. In SCI, Cx43 demonstrates a wave-like spatiotemporal expression pattern. During the first 1–3 days, epitope masking occurs in gray matter regions with moderate neuronal loss; by day 7, Cx43 disappears from the lesion epicenter but remains localized in GFAP-positive astrocytes in the subpial rim and perivascular regions, reflecting astrocyte transition to a reactive phenotype and involvement in tissue remodeling [95]. Trauma induces excessive ATP release through Cx43 hemichannels, activating P2X7 purinergic receptors and amplifying neuroinflammation, edema, and secondary damage [10]. Such dynamic remodeling of connexin expression further supports the concept that Cx-dependent communication represents a component of injury-induced plasticity, contributing both to inflammatory propagation and to structural and metabolic adaptation of neural tissue.

Combined deletion of Cx43/Cx30 markedly suppresses neuropathic pain, exceeding the analgesic effect of minocycline, while reducing astrogliosis and microglial activation [94]. Selective hemichannel inhibitors (Peptide5, Gap27) in compression and partial transection SCI models reduce Cx43 expression, increase its phosphorylation, decrease TNF-α and IL-1β levels, suppress astrogliosis [11] and ferroptosis [98], stabilize water homeostasis [97], and significantly improve hindlimb locomotor function [12]. HF-rTMS inhibits Cx43 expression and activates autophagy through the mTOR pathway, promoting motor recovery [100]. Furthermore, interaction between Cx43 and sigma-1 receptors (Sig-1R) contributes to neuropathic pain, and pharmacological inhibition of Cx43 (BD1047, carbenoxolone, 43Gap26) disrupts this interaction and alleviates allodynia [101]. HBO demonstrates a phase-dependent effect, decreasing Cx43 transcription and translation during early stages while increasing its expression after two weeks, potentially facilitating regenerative processes [103].

Neuronal connexins Cx36 and Cx32 generally display protective roles during ischemia and injury. Cx36 knockout reduces CSD and infarct size [13], as well as NMDA-induced neurotoxicity [50], whereas Cx32 protects interneurons [16] and oligodendrocytes through autophagic and mitophagic mechanisms [17]. In peripheral nerve injury, Cx32 is critical for myelination, and its mutations or knockout lead to demyelinating neuropathies [15,116]. Cx40, expressed in endothelial cells and cardiomyocytes, modulates post-stroke arrhythmias [53] and vasospasm [54], while Cx37 represents a genetic susceptibility factor for ischemic stroke [57,58].

In Alzheimer’s disease, Cx43 and Cx30 accumulate around Aβ plaques [18,131], enhancing hemichannel activity, Ca^2+^ overload, and neuroinflammation. This initiates a pathological feedback loop mediated by ATP and glutamate release through Cx43 hemichannels [21], followed by activation of purinergic receptors and increased oxidative stress and inflammation [135,140]. In AD models including 5XFAD and APP/PS1, expression of Cx43 and Cx30 increases in the cortex, thalamus, and spinal cord, particularly in reactive astrocytes surrounding dense plaques, whereas Cx47 decreases, disrupting astrocyte–oligodendrocyte coupling and contributing to demyelination and progressive neurodegeneration [18,131,134]. Cx43 knockout reduces MAM contacts with MFN2, Aβ accumulation, neuroinflammation, and cognitive deficits by activating autophagy and reducing apoptosis [138]. Additionally, blockade of Cx43 hemichannels restores astrocytic Aβ phagocytosis and enhances mitochondrial transfer via CD38, demonstrating neuroprotective potential [139]. Interaction between Cx43 and A2AR forms a positive feedback loop that enhances Aβ toxicity; inhibition of A2AR or CD73 attenuates hemichannel activity, emphasizing the role of purinergic signaling [140,141]. In the cortex of patients with Alzheimer’s disease, Cx43 is overexpressed in astrocytic GJs surrounding β/A4-amyloid plaques, which may both support neuronal homeostasis and contribute to cytotoxicity [136], while the direct interaction of Aβ with Cx43 disrupts the structural and functional organization of GJs [133].

In PD, Cx43 may be reduced at late stages in the cortex and basal ganglia, correlating with astrocytic arbor degradation, depression, and insomnia [143], but it may also be increased in rotenone- [145] or LPS-induced PD models, with enhanced phosphorylation in the substantia nigra and globus pallidus [144]. Its inhibition by Gap27 protects dopaminergic neurons and restores dopamine and metabolite levels by suppressing hemichannel activity and neuroinflammation [22,146]. Cx30 knockout accelerates the loss of dopaminergic neurons in the MPTP model, weakening astrogliosis and reducing GDNF expression, highlighting its protective role in maintaining neurotrophic mechanisms [147]. Cx36 modulates synchronization in the striatum and motor cortex, with increased expression in ENK^+^ and PV^+^ neurons in the 6-OHDA model, leading to desynchronization of cortico-basal networks and progression of motor deficits [149]. Its pharmacological blockade (carbenoxolone, quinine) alleviates levodopa-induced dyskinesia [150]. Cx32 contributes to the spread of α-synuclein through uptake and intercellular transfer, as confirmed in postmortem samples from PD patients [152], while Cx26, associated with EGFR, is upregulated in MPP^+^ models and may participate in neuronal death [154]. Gastrodin [145] and baicalin [151] normalize Cx43 and Cx36 expression, exerting a neuroprotective effect.

In amyotrophic lateral sclerosis, astrocytic Cx43 overexpression in SOD1 G93A models creates a toxic feedback loop involving increased intercellular coupling, Ca^2+^ accumulation, and motor neuron degeneration [23]. Pharmacological inhibitors (Gap19, tonabersat) or genetic deletion of Cx43 reduce astroglial and microglial activation and improve survival [155]. Cx30 knockout delays disease onset and reduces neuroinflammation [159], whereas Cx36 knockout attenuates motor neuron death [160]. Meanwhile, Cx47 and Cx32 decline in late disease stages, correlating with reduced EAAT2, axonal degeneration, and microglial activation [19]. Insulin interacts with Cx43 and Cx31, blocking the channels and potentially explaining the protective effect of hyperinsulinemia in ALS [161].

In Huntington’s disease, Cx43 is overexpressed in the striatum in association with reactive astrogliosis and heterogeneous distribution within the caudate nucleus [24]. Retinal studies demonstrate decreased Cx36 and markedly reduced Cx45, potentially contributing to visual processing deficits [162]. In the middle cingulate cortex, reduced EAAT2 expression in the context of Cx43-dependent gliosis exacerbates symptoms [163].

In MDD, a persistent reduction in the expression and functional activity of astrocytic Cx43 and Cx30 is observed in the prefrontal cortex [165], hippocampus [26], and orbitofrontal cortex [174]. This disrupts the integrity of the astrocytic syncytium, reduces intercellular communication through GJs, and enhances neuroinflammation, leading to depression-like behavior [25]. In models of CUS, CSDS, and CORT, the reduction in Cx43 is accompanied by accelerated protein degradation via the ubiquitin–proteasome and autophagy–lysosomal pathways [169,170], activation of pro-inflammatory signaling cascades including JAK2–STAT3 with increased expression of TSPO as a marker of microglial activation [167], and NF-κB signaling including phosphorylation of p65 [168]. Pharmacological blockade of Cx43 or conditional knockout in the PFC reproduces a depression-like phenotype in otherwise intact animals, including anhedonia, increased immobility time in forced swimming and tail suspension tests, as well as systemic and central inflammation [165]. Epigenetic mechanisms also contribute: in depressed suicide victims, enrichment of the repressive histone mark H3K9me3 has been detected in the promoter regions of the Cx43 and Cx30 genes in the PFC, resulting in long-term repression of astrocytic connexins [171].

Restoration of Cx43 function demonstrates pronounced antidepressant effects. Ginsenoside Rg1 increases Cx43 biosynthesis, suppresses its ubiquitination [169,170], and inhibits nuclear translocation of YAP [179], thereby limiting neuroinflammation and restoring functional connectivity in the PFC [179] and hippocampus [180]. Loganin increases Cx43 expression, reduces phosphorylation, and activates the GSK-3β/β-catenin pathway, showing synergism with fluoxetine and accelerating the onset of therapeutic effects [184]. Genistein suppresses miR-221/222, increasing Cx43 levels and reversing depression-like behavior [185]. Fluoxetine normalizes the CORT-induced increase in Cx43 phosphorylation in the hippocampus [175] and enhances antidepressant effects during Cx43 knockdown [175] via cAMP-dependent mechanisms [177]. In addition, the selective hemichannel inhibitor D4 reduces neuroinflammation, normalizes neuronal activity in the hippocampus, entorhinal cortex, and lateral septum, and eliminates depression-like behavior in LPS- and stress-induced depression models [188]. Clinical data show that patients with MDD and comorbid chronic insomnia exhibit reduced serum levels of Cx43, Cx30, and AQP4, which correlate with cognitive impairment and reduced slow-wave sleep [173]. Postmortem studies reveal more than a 60% reduction in Cx43 in the orbitofrontal cortex of patients with depression and alcoholism, accompanied by a decrease in the area and size of immunoreactive puncta [174].

In BD, a proposed model explains mood fluctuations through region-specific dysregulation of Cx43 within the astrocytic syncytium and tripartite synapses. Depressive episodes are associated with decreased Cx43 expression, which disrupts astrocytic communication, increases compensatory expression of astrocytic receptors, slows synaptic information processing, leads to neurotransmitter deficiency, and results in prolonged activation of behavioral generation systems [27]. In contrast, manic episodes are characterized by increased Cx43 expression, enhanced intercellular communication, reduced receptor expression, accelerated synaptic transmission, excess neurotransmitters, and rapid behavioral switching [190]. Chronic administration of zotepine at therapeutic doses increases membrane expression of Cx43 in primary cortical astrocytes and promotes L-glutamate release through activated hemichannels; this effect is mediated via the Akt pathway. Supratherapeutic doses amplify these changes and exhibit pro-convulsant effects, highlighting the dependence of the stabilizing effect on Cx43-mediated astroglial glutamatergic transmission [191]. Mutations in the CACNA1C gene, a genetic risk factor for BD, disrupt intercellular communication via Cx43 in cardiomyocytes, slowing impulse conduction and increasing proarrhythmic risk during psychotropic therapy. Gene therapy restoring Cx43 expression improves conduction and protects against QT-interval prolongation [192].

In schizophrenia, genetic associations with the 1q21.1 region (genes Cx40 and Cx50) reveal an increased frequency of the Cx50 haplotype in patients and increased transmission within families, indicating genetic susceptibility [194]. Reduced expression of Cx30 and Cx43 in the dorsolateral prefrontal cortex and anterior cingulate cortex of patients with schizophrenia and suicidal behavior correlates with cognitive deficits, impaired functional connectivity, and loss of the ability to differentiate cognitive domains, which underlies generalized cognitive impairment [20,28]. Atypical antipsychotics (clozapine, quetiapine, brexpiprazole) and mood stabilizers (valproate) enhance astroglial L-glutamate release via Cx43 hemichannels by increasing membrane expression of Cx43 (clozapine and quetiapine via Akt, brexpiprazole independently), which may contribute to their therapeutic mechanisms in schizophrenia and affective disorders [197].

In temporal lobe epilepsy, Cx43 and Cx30 are overexpressed in the hippocampus and perivascular astrocytic endfeet during the latent and chronic stages, increasing BBB permeability [201], albumin extravasation [200], Ca^2+^ waves, and hypersynchronization of neuronal networks [199,203]. Redistribution of Cx43 to perivascular endfeet and increased phosphorylation correlate with reactive astrogliosis and neuronal loss [200]. Selective hemichannel inhibitors reduce BBB permeability, seizure activity on EEG, neuroinflammation, and restore synaptic inhibition more effectively than non-specific blockers such as carbenoxolone, demonstrating the superiority of targeted approaches [201,211].

These data strongly support the therapeutic potential of selective Cx43 hemichannel inhibitors such as Gap19, Peptide5, TAT-Gap19, D4, and tonabersat as novel neuroprotective agents that preserve protective GJs. Plant-derived compounds, including ginsenoside Rg1, loganin, baicalin, and Tongxinluo, as well as traditional approaches such as acupuncture and hyperbaric oxygen therapy, demonstrate translational clinical potential through normalization or phase-dependent restoration of connexin function.

Thus, Cxs, particularly Cx43, emerge as important therapeutic targets in neurology, linking acute and chronic neurological disorders through the regulation of neuroglial communication, neuroinflammation, and cell fate. The transition from non-specific connexin blockers to selective context- and phase-dependent modulation of Cx activity represents a promising strategy for the development of more precise and effective therapies for central nervous system diseases.

## 6. Limitations of the Study

Several limitations of this review should be acknowledged. First, the study is based on a narrative synthesis rather than a quantitative meta-analysis, as the included studies exhibit substantial heterogeneity in terms of experimental models, animal species, disease stages, and methodological approaches. Such variability limits the possibility of direct statistical comparison and requires cautious interpretation of the findings. Second, a considerable proportion of the analyzed data originates from experimental animal models and in vitro studies, which may only partially reflect the complexity of pathological processes occurring in the human nervous system. Third, differences in experimental techniques, genetic models, and pharmacological tools used to investigate Cx may partly account for the inconsistencies observed across studies. In addition, although a systematic search was conducted in major scientific databases, the potential influence of publication bias cannot be completely excluded. Finally, a formal quantitative assessment of risk of bias and evidence grading was not performed, and the methodological quality of the included studies was evaluated primarily through qualitative analysis.

## 7. Conclusions

Cxs represent key regulators of intercellular communication in both the CNS and PNS, coordinating neuronal and glial activity through the formation of GJs and hemichannels. The synthesis of extensive experimental and clinical evidence presented in this review convincingly demonstrates that multiple Cx isoforms, particularly Cx43, Cx30, Cx36, and Cx32, play important and often dual roles in the pathogenesis of a broad spectrum of nervous system disorders, ranging from acute injuries, including stroke, TBI, SCI, and PNI, to chronic neurodegenerative and psychiatric disorders.

The biological effects of Cxs are highly dependent on cell type, disease stage, and various contextual factors. They may contribute both to the propagation of injury and neuroinflammation and to essential neuroprotective mechanisms, including spatial buffering of ions, redistribution of metabolites, and coordination of glial responses. This dual nature is particularly evident in acute CNS injury, where dynamic changes in Cx43 expression and function influence both the progression of secondary damage and the development of adaptive and reparative processes. A similar pattern is observed in chronic neurodegenerative and psychiatric disorders, where alterations in Cx-dependent signaling more often reflect mechanisms that amplify or propagate pathological processes, including chronic inflammation, impaired neuroglial communication, and network dysfunction.

The body of evidence presented here highlights the need to move beyond the simplified concept of non-specific blockade of connexin channels toward a more advanced paradigm of selective and phase-dependent modulation of Cx-mediated intercellular communication. Many contemporary therapeutic approaches primarily aim to inhibit pathologically activated hemichannels while preserving the protective functions of GJs. In some cases, restoration or normalization of physiological intercellular communication may be as important a therapeutic strategy as suppression of pathological channel activation.

Thus, Cxs, and particularly Cx43, represent an important integrative component linking neuroinflammation, metabolic coordination, and neuroglial communication across various nervous system disorders. The transition from non-specific blockers toward selective and phase-dependent modulation of Cx activity opens promising perspectives for the development of novel neuroprotective and neuroregenerative strategies in modern neurology and psychiatry. Of particular interest are emerging data demonstrating the potential to modulate Cx-dependent pathways using mimetic peptides, selective hemichannel inhibitors, natural compounds, cell-based therapies, and non-pharmacological interventions that influence intracellular signaling cascades. Expanding knowledge of Cx involvement in mitochondrial regulation, autophagy, exosome-mediated signaling, and neuroinflammatory pathways provides new opportunities for the development of tissue-specific and phase-specific therapeutic strategies targeting nervous system diseases.

Despite significant progress, important questions remain regarding the distinct functions of GJs and hemichannels, the regulatory mechanisms of specific Cx isoforms in different cell types, and the influence of compensatory mechanisms in genetic knockout models. Further research aimed at clarifying the molecular mechanisms of Cx-mediated communication and developing selective modulators may facilitate the creation of more precise and effective therapeutic approaches for both acute and chronic disorders of the nervous system.

Thus, Cxs, particularly Cx43, represent an important integrative link connecting neuroinflammation, metabolic coordination, and neuroglial communication across diverse CNS and PNS pathologies. The transition from non-specific blockers toward phase-dependent modulation of Cx activity represents a promising direction for the development of neuroprotective and neuroregenerative strategies in modern medicine.

## Figures and Tables

**Table 1:** Parameters of the search strategy in scientific databases.

Database	Keywords and Search Terms	Boolean Operators	Limits
PubMed	“connexins”, “gap junctions”, “hemichannels”, “Cx43”, “Cx36”, “Cx32”, “astrocytes”, “glial cells”, “brain injury”, “traumatic brain injury”, “spinal cord injury”, “peripheral nerve injury”, “stroke”, “cerebral ischemia”, “neurodegeneration”, “Alzheimer’s disease”, “Parkinson’s disease”, “amyotrophic lateral sclerosis”, “Huntington’s disease”, “psychiatric disorders”, “schizophrenia”, “depression”, “bipolar disorder”, “epilepsy”, “neuroinflammation”	AND, OR, NOT	Publication years 2015–2026; no language restrictions; full-text availability not required
Scopus	“connexins”, “gap junctions”, “hemichannels”, “Cx43”, “Cx36”, “Cx32”, AND (“brain injury” OR “traumatic brain injury” OR “spinal cord injury” OR “stroke” OR “cerebral ischemia” OR “neurodegeneration” OR “Alzheimer’s disease” OR “Parkinson’s disease” OR “amyotrophic lateral sclerosis” OR “psychiatric disorders” OR “schizophrenia” OR “depression” OR “epilepsy”)	AND, OR	Publication years 2015–2026; no language restrictions
Web of Science	(“connexins” OR “gap junctions” OR “hemichannels” OR “Cx43” OR “Cx36” OR “Cx32”) AND (“brain injury” OR “traumatic brain injury” OR “spinal cord injury” OR “stroke” OR “neurodegeneration” OR “Alzheimer’s disease” OR “Parkinson’s disease” OR “amyotrophic lateral sclerosis” OR “psychiatric disorders” OR “schizophrenia” OR “depression” OR “epilepsy”) AND (“astrocytes” OR “glial cells” OR “neuroinflammation”)	AND, OR	Publication years 2015–2026; no language restrictions

**Table 2:** Role of Connexins in Stroke: Experimental Models, Modulation Approaches, and Key Results. Arrows indicate direction of change or relationship: ↑ increase; ↓ decrease; → indicates effect or association.

No.	Experimental Model	Subject (Animals/Cells)	Stroke Type	Connexin Isoform	Cx Modulation	Key Results	Reference
1	in vitro (OGD/R) + in vivo (MCAO)	Primary astrocytes, mixed astrocyte + cortical neuron culture; rats	Ischemic (OGD/R and MCAO)	Cx43	Cx43 overexpression (vector transfection)	OGD/R → ↓ Cx43 expression + cytoplasmic translocation, ↑ Ca^2+^, CaMKII/CREB activation, ↑ Ephrin-A4, astrocyte contraction and neurite growth suppression; overexpression fully reverses all changes after OGD/R and restores neurite growth (via ↓ Ephrin-A4); in vivo Cx43 restoration correlates with neuronal recovery after MCAO	[3]
2	in vivo (MCAO + intra-arterial BMSC administration)	Wistar rats	Ischemic (MCAO)	Cx43	Increased Cx43 expression via BMSC	MCAO 2 h; BMSC administered 24 h post-MCAO; significant functional recovery improvement, ↑ proliferation (BrdU^+^, Ki67^+^), astrocyte differentiation and Cx43 + synaptophysin expression in the peri-infarct zone; BMP2/4–Cx43 and Cx43–synaptophysin correlations	[4]
3	in vivo (MCAO + enriched environment)	Rats	Ischemic/reperfusion (MCAO)	Cx43	Decreased Cx43 expression (EE + Gap19)	MCAO + reperfusion; EE and Gap19 significantly improve neurological outcome, ↓ infarct volume, neuronal damage, inflammatory cytokines and oxidative stress; Gap19 enhances EE neuroprotection by suppressing Cx43 and the TLR4/MyD88/NF-κB pathway	[6]
4	in vitro (hemin) + in vivo (collagenase IV)	Primary astrocytes; C57BL/6J mice	Intracerebral hemorrhage (ICH)	Cx43 (Cx43 hemichannels)	Gap19 (hemichannel inhibitor + ↓ expression via ubiquitination)	Post-ICH (in vivo) delayed Gap19 administration significantly ↓ hematoma volume, neurological deficits, inflammatory cytokines and reactive astrogliosis (via ↑ nuclear YAP translocation → ↑ SOCS1/3 → inhibition of TLR4-NFκB and JAK2-STAT3); effect fully blocked by verteporfin	[7]
5	in vivo (MCAO) + in vitro (OGD)	Mice	Ischemic/reperfusion (MCAO)	Cx43	Gap19 (specific hemichannel inhibitor)	Gap19 administered immediately (i.c.v.) or TAT-Gap19 4 h post-reperfusion; significantly ↓ infarct volume, neuronal damage, neurological deficits, caspase-3 and Bax, ↑ Bcl-2; activates JAK2/STAT3 (stronger effect than Gap26); neutralized by AG490	[8]
6	in vivo (MCAO) + in vitro (OGD + LPS)	Mice	Ischemic/reperfusion (MCAO)	Cx43	Gap19 (selective hemichannel inhibitor)	MCAO; Gap19 improves neurology, ↓ infarct volume and white-matter damage; suppresses Cx43, TLR4 and cytokines (TNF-α, IL-1β); inhibits hemichannels in astrocytes	[9]
7	in vivo (distal MCAO)	Cx36 KO and WT mice	Ischemic (distal MCAO)	Cx36	Cx36 knockout	After distal MCAO, Cx36 deletion significantly ↓ frequency and duration of CSD, ↓ infarct size and improves motor function	[13]
8	in vivo (global ischemia)	Mice (including Cx32 Y/− KO)	Global ischemia	Cx32, Cx36, Cx43	Observation of ↑ Cx32/Cx36 + Cx32 KO	Global ischemia → selective post-translational ↑ of Cx32 and Cx36 proteins in parvalbumin^+^ CA1 interneurons before neuronal death; Cx32 KO increases vulnerability	[16]
9	in vitro (OGD/R) + in vivo (I/R)	Neurons	Ischemic/reperfusion	Cx32	Cx32 inhibition	OGD/R and I/R ↑ Cx32 and autophagy; Cx32 inhibition further activates autophagy/mitophagy via Nur77 (mitochondrial translocation) and protects neurons	[17]
10	ex vivo (postmortem brain slices)	Human (acute and chronic ischemia)	Ischemic	Cx43	Observation of ↑ Cx43 in chronic phase	In chronic model (multiple infarcts) significantly ↑ Cx43 immunoreactivity in astrocytes of the peri-infarct zone (neurons better preserved than in acute model)	[37]
11	in vivo (pMCAO)	Rats/mice	Ischemic (permanent MCAO)	Cx43	Observation of Cx43 dynamics	2–3 h after pMCAO Cx43 ↑ in ischemic core, sharply ↓ at 6 h (due to cell death); in peri-infarct—intense staining	[38]
12	in vivo (neonatal HI) + hUCB	Rats	Perinatal hypoxic–ischemic injury	Cx43	hUCB transplantation (↓ Cx43)	HI induces reactive astrogliosis + Cx43 regulation; hUCB (short- and long-term) accelerates inflammation resolution and ↓ Cx43 expression	[39]
13	in vivo (MCAO)	Cx43+/− and WT mice	Ischemic (MCAO)	Cx43	Heterozygous Cx43 KO	At 24 h and 4 days post-MCAO, Cx43+/− show significantly larger infarct, ↑ apoptosis (caspase-3) and ↓ astrogliosis; ↑ Cx30 in penumbra	[40]
14	in vivo (focal MCAO)	Cx43+/− and WT mice	Ischemic	Cx43	Heterozygous Cx43 KO	At 4 days post-MCAO, infarct volume in Cx43+/− is significantly larger	[41]
15	in vivo (MCAO)	Astrocyte-specific Cx43 KO (Cre+)	Ischemic	Cx43	Astrocyte-specific KO	At 4 days post-MCAO, astrocyte KO → ↑ infarct volume, ↑ apoptosis (TUNEL, caspase-3) and inflammation (CD11b)	[42]
16	in vivo (MCAO) + in vitro	Cx43ΔCT mice	Ischemic (MCAO)	Cx43	Truncated C-terminus of Cx43	At 4 days post-MCAO, Cx43ΔCT → enhanced brain damage, ↓ astrogliosis, ↑ inflammatory invasion; altered channel activity and Ca^2+^ waves in culture	[43]
17	in vivo (pMCAO)	MK4 transgenic mice + WT	Ischemic (pMCAO)	Cx43	MAPK-site mutations (MK4) + TAT-Gap19	MK4 significantly ↓ infarct volume and improves behavior; TAT-Gap19 is also neuroprotective	[44]
18	in vivo (bilateral carotid artery occlusion)	Rats	Global ischemia	Cx43 (+ Cx40)	ERK inhibitors + siRNA	Post-ischemia ↑ Cx43 phosphorylation; ERK inhibition prevents brain damage and protects BBB	[45]
19	in vivo (MCAO)	Cx43±, AQP4−/− + CMP mice	Ischemic (MCAO)	Cx43	Heterozygous Cx43± + CMP	Post-MCAO, Cx43± and CMP → ↑ infarct volume, ↓ neurogenesis in SVZ and peri-infarct, ↑ IL-1β/TNF-α	[46]
20	in vivo (MCAO) + in vitro (OGD/R)	Mice + astrocyte cultures	Ischemic (MCAO)	Cx43 (+ AQP4)	SKF-86002 (↓ Cx43/AQP4)	SKF-86002 ↓ infarct, edema, apoptosis, astrocyte proliferation and inflammation	[47]
21	ex vivo (NVU analysis)	Cx30/Cx43 KO and Cx43ΔCT transgenic mice	Baseline NVU organization	Cx43 (+ Cx30)	Cx30/Cx43 KO and Cx43ΔCT	Absence of Cx43/C-terminus → disrupted AQP4 organization, ↑ microhemorrhages and BBB permeability	[48]
22	in vivo (microsphere embolism) + NPC	Rats	Severe cerebral ischemia	Cx43	NPC (↑ Cx43)	Ischemia ↓ Cx43 in capillaries; NPC restore Cx43 and remodel NVU	[49]
23	in vivo (NMDA + photothrombotic ischemia)	WT and Cx36 KO mice	Excitotoxicity + ischemia	Cx36	Cx36 KO + mefloquine	Cx36 KO and blockade completely prevent NMDAR-dependent neurodegeneration and ↓ ischemic neuronal death	[50]
24	in vitro (organotypic culture + OGD)	Rat hippocampal culture + Cx36-CFP mice	Ischemic (OGD)	Cx36	Carbenoxolone (GJ blocker)	At 4 h and 24 h after 30 min OGD, resilient interneurons express Cx36; GJ blockade sharply reduces pyramidal neuron death	[51]
25	in vivo (BCAS)	Mice	Ischemic white-matter damage	Cx30/Cx32	BCAS (↓ Cx30/Cx32)	At 30 days post-BCAS, A/O gap junctions are disrupted, oligodendrogenesis ↓ and cognitive deficits appear	[52]
26	in vivo (MCAO) + rAAV9-Gja5	Mice	Ischemic (MCAO)	Cx40	Cx40 overexpression (rAAV9-Gja5)	Permanent MCAO ↓ Cx40 in sinoatrial node; Cx40 restoration significantly improves post-stroke arrhythmia	[53]
27	in vivo (SAH)	Rats	Subarachnoid hemorrhage (SAH)	Cx40	40Gap27 (Cx40 inhibitor)	Post-SAH NO/cGMP/PKG ↑ Cx40; 40Gap27 completely blocks vasodilation	[54]
28	in vitro (OxyHb) + in vivo (SAH)	Smooth muscle cells; rats	SAH + cerebral vasospasm	Cx43	Cx43 siRNA + PKC inhibitors	SAH ↑ Cx43/GJIC via PKC; Cx43 siRNA and PKC inhibitors completely abolish CVS and DCI (day 7)	[55]
29	in vivo (SAH)	Rabbits	Subarachnoid hemorrhage (SAH)	Cx43/Cx45 (heteromers)	CBX (blocker)	SAH day 7 → significant ↑ Cx43/Cx45 heteromers; CBX suppresses vasospasm	[56]
30	Genetic analysis (SNP)	Patients	Ischemic stroke	Cx37	Genetic polymorphisms	AG/GG rs1764390 and CC rs1764391 genotypes ↑ risk of ischemic stroke	[57]
31	Genetic analysis (SNP + GMDR)	Patients	Ischemic stroke	Cx37 (+ PDE4D)	Genetic polymorphisms + smoking	rs1764391-T and rs966221-G + interactions with rs918592/smoking significantly ↑ ischemic stroke risk	[58]
32	in vivo (SAH) + transcriptomics	Astrocytes (SAH model)	Subarachnoid hemorrhage (SAH)	Cx43	Cx43–ACKR3 axis modulation	Cx43–ACKR3 axis enhances neuroinflammation and neuronal apoptosis after SAH	[59]
33	in vivo + in vitro (SY5Y, U87)	Animals + cell lines	Ischemic stroke	Cx43	Leptin (↓ Cx43 via ERK1/2)	Leptin ↓ infarct volume and ↑ Cx43 in vivo; in vitro ↓ Cx43, glutamate and apoptosis (blocked by ERK1/2 inhibitors)	[60]
34	in vivo (I/R) + H-89	Sprague-Dawley rats	Ischemia–reperfusion	Cx36	Astragaloside IV (↑ Cx36/PKA) + H-89	I/R 2 h + 24 h reperfusion; AST-IV ↓ infarct and apoptosis; ↑ Cx36/PKA, ↓ Bax/Bcl-2 (effect abolished by H-89)	[61]
35	in vitro (OGD PC12)	PC12 cells	Ischemic (OGD)	Cx36	Leonurine (↓ Cx36/CaMKII)	OGD ↑ Cx36 and pCaMKII; leonurine ↑ viability and ↓ apoptosis (effect via Cx36/CaMKII)	[62]
36	in vivo (MCAO) + CBX	Sprague-Dawley rats	Ischemia–reperfusion (I/R)	Cx43	TXL (Cx43 regulation) + CBX	MCAO 90 min; TXL ↓ neurological deficit and apoptosis on days 3, 7 and 14; CBX completely abolishes the effect	[63]
37	in vivo (embolic stroke + delayed rt-PA)	Rats	Ischemic (with delayed thrombolysis)	Cx43	Acupuncture (↓ ERK1/2-Cx43)	Acupuncture ↓ infarct volume, improves neurology and BBB by suppressing neurotoxic astrocyte polarization	[64]
38	in vivo (I/R) + 8-Bromo-cAMP/H89	Lewis rats	Ischemia–reperfusion (I/R)	Cx43 (exosomal)	PKA activation (8-Bromo-cAMP)	8-Bromo-cAMP ↑ Cx43-containing exosomes, improves cognitive function and BBB	[65]
39	in vivo (focal ischemia)	Rats	Ischemic	Cx43	Observation of ↑ Cx43	Within 60 days post-ischemia, reactive astrocytes ↑ Cx43 and proliferation in the glial scar	[66]
40	in vivo (MCAO + delayed rt-PA)	SHR rats	Ischemic (with hemorrhagic transformation)	Cx43 (p-Cx43)	Cx43 inhibitors	MCAO 1.5 h + rt-PA at 4.5 h; assessment at 24 h; rt-PA ↑ p-Cx43 → HT; Cx43 inhibitors attenuate HT	[67]
41	in vivo (MCAO 90 min + 7 days) + fluorocitrate	Sprague-Dawley rats	Ischemic (MCAO)	Cx43 (+ GFAP)	Inhibition of reactive astrocytes (fluorocitrate)	MCAO 90 min + 7 days reperfusion; fluorocitrate (from reperfusion onset) fully restores SYP/CRTC1 and memory	[68]
42	in vivo (dMCAO) + in vitro (TSZ)	Rats + SH-SY5Y	Ischemic (dMCAO) + thalamic damage	Cx43	MLKL–Cx43 interaction	dMCAO ↑ MLKL and Cx43 in thalamus; MLKL opens Cx43 hemichannels → ↑ Ca^2+^ and necroptosis	[69]

Abbreviations: OGD/R—oxygen–glucose deprivation/reperfusion; MCAO—middle cerebral artery occlusion; pMCAO—permanent middle cerebral artery occlusion; dMCAO—distal middle cerebral artery occlusion; I/R—ischemia/reperfusion; ICH—intracerebral hemorrhage; SAH—subarachnoid hemorrhage; HI—hypoxia–ischemia; BCCAO—Bilateral Common Carotid Artery Occlusion; CSD—cortical spreading depolarization; Cx43, Cx36, Cx32, Cx40, Cx30, Cx37, Cx45—connexins 43, 36, 32, 40, 30, 37 and 45, respectively; KO—knockout; Cx43ΔCT—truncated C-terminal domain of connexin 43; BMSC—bone marrow stromal cells; hUCB—human umbilical cord blood cells; NPC—neural progenitor cells; EE—enriched environment; Gap19, Gap26, Gap27, 40Gap27—connexin mimetic peptide inhibitors; CBX—carbenoxolone; TXL—Tongxinluo; AST-IV—astragaloside IV; NVU—neurovascular unit; BBB—blood–brain barrier; DCI—delayed cerebral ischemia; CVS—cerebral vasospasm; SNP—single nucleotide polymorphism; BrdU—5-bromo-2′-deoxyuridine; TUNEL—terminal deoxynucleotidyl transferase dUTP nick end labeling; GFAP—glial fibrillary acidic protein; AQP4—aquaporin-4; rt-PA—recombinant tissue plasminogen activator; HT—hemorrhagic transformation; SYP—synaptophysin; CRTC1—CREB-regulated transcription coactivator 1; MLKL—mixed lineage kinase domain-like protein; TSZ—necroptosis inducer (TNF-α + Smac mimetic + z-VAD-fmk); YAP—Yes-associated protein; PKA—protein kinase A; ERK1/2—extracellular signal-regulated kinase 1/2; TLR4—Toll-like receptor 4; MyD88—myeloid differentiation primary response 88; NF-κB—nuclear factor kappa B; SOCS1/3—suppressor of cytokine signaling 1/3; JAK2–STAT3—Janus kinase 2—signal transducer and activator of transcription 3; Bcl-2—B-cell lymphoma 2 protein; Bax—Bcl-2-associated X protein; AG490—JAK2 inhibitor; Nur77—nuclear receptor subfamily 4 group A member 1; SVZ—subventricular zone; CD11b—integrin alpha M microglial marker; PKC—protein kinase C; NO—nitric oxide; cGMP—cyclic guanosine monophosphate; PKG—protein kinase G; ACKR3—atypical chemokine receptor 3; CaMKII—calcium/calmodulin-dependent protein kinase II; NMDAR—N-methyl-D-aspartate receptor; CFP—cyan fluorescent protein; rAAV9—recombinant adeno-associated virus serotype 9.

**Table 3:** Role of Connexins in Traumatic Brain Injury: Experimental Models, Modulation Approaches, and Key Results. Arrows indicate direction of change or relationship: ↑ increase; ↓ decrease.

No.	Experimental Model	Subject (Animals/Cells)	TBI Type	Connexin Isoform	Cx Modulation	Key Results	Reference
1	in vitro (compressed nitrogen-oxygen gas) + co-culture	Primary cortical neurons + astrocytes (C57BL/6 mice)	in vitro TBI model	Cx43 (GJA1-20K)	GJA1-20K overexpression	GJA1-20K overexpression ↓ p-Cx43, ↑ dendrite length, ↑ mitochondrial function and mitochondrial transfer from astrocytes to neurons; protects neurons	[5]
2	in vivo (FPI)	Sprague-Dawley rats	Moderate FPI (2.6–2.8 atm)	Cx43 (p-Cx43)	Observation of ↑ p-Cx43	p-Cx43 ↑ in ipsilateral hippocampus at 1 h, peak at 6 h, persists up to 24 h; localized in astrocytes around CA3 neurons; colocalization with p-ERK	[71]
3	in vivo (FPI)	Sprague-Dawley rats	Moderate FPI	Cx43 and Cx36	Observation	Cx43 ↑ in reactive astrocytes at 24–72 h; Cx36 ↑ in CA3 neurons at 1 h, then ↓	[72]
4	in vivo (CCI) + AS-ODN	Rats	CCI	Cx43	AS-ODN against Cx43 (intracerebroventricular)	AS-ODN ↓ Cx43 expression, ↓ brain edema and reactive astrogliosis at 6, 24 and 48 h post-CCI	[73]
5	in vivo (weight drop)	Sprague-Dawley rats	Weight drop	Cx43 (p-Cx43) + LC3-II	Observation + p-Cx43 inhibitor	p-Cx43 ↑ (peak at 6 h); LC3-II ↑ up to 24 h; p-Cx43 inhibition ↓ autophagy in hippocampal neurons	[74]
6	in vivo (TBI)	Rats	TBI	Cx43 (p-Cx43)	3-methyladenine (autophagy inhibitor)	Neuronal autophagy ↓ p-Cx43 level in hippocampal astrocytes after TBI	[75]
7	in vivo (CCI) + H2	Mice + astrocytes (LPS)	CCI	Cx43	H2 (molecular hydrogen)	H2 ↑ NEDD4, enhances mitophagy, ↓ Cx43; NEDD4 ubiquitinates Cx43; improves cognitive function	[76]
8	in vivo (CCI) + NAC	Wistar rats	CCI	Cx40	NAC (antioxidant)	CCI ↑ Cx40 linearly with oxidative stress; NAC ↓ Cx40, neurological deficits and oxidative stress	[77]
9	in vivo (TBI) + GS-Rb1	Mice	TBI	Cx40	GS-Rb1 (ginsenoside Rb1)	GS-Rb1 ↓ brain damage and ↑ Cx40 at 6 h; effect via ERK1/2 (abolished by U0126)	[78]
10	in vitro (pulsatile shear stress) + in vivo (CCI)	SH-SY5Y cells + rats	CCI	Cx43	miR-302 (overexpression)	Pulsatile stress ↑ p-ERK1/2 and p-Cx43; miR-302 ↓ Cx43 phosphorylation, improves cognitive function and ↓ brain damage after CCI	[79]
11	in vivo (CCI) + NSC transplantation	Wistar rats	CCI	Cx43	NSC transplantation	NSC ↑ Cx43 expression at transplantation site and CCI border at 1, 2 and 4 weeks (*p* < 0.01–0.05); promotes integration	[80]
12	in vivo (TBI) + in vitro (NSC)	NSC + TBI model	TBI	Cx43	αCT1 peptide (Cx43 modulator)	αCT1 ↓ NSC proliferation and ↑ caspase 3/7; ↓ total Cx43 and p-S368; Cx43 positively regulates NSC after TBI	[81]
13	in vivo (weight drop)	Sprague-Dawley rats	Weight drop (modified model)	Cx43 + occludin	Observation	Cx43 ↑ (peak at 24 h), occludin ↓ (minimum at 3 days); changes correspond to brain edema development	[82]
14	in vivo (weight drop) + BMSC	Rats	Weight drop	Cx43	BMSC (mesenchymal stem cells)	BMSC ↓ Cx43, LC3 and beclin-1 expression in hippocampus; suppress autophagy after TBI	[83]
15	in vivo (TBI)	Rats	TBI	Cx43	CBX + 3-MA	CBX and 3-MA ↓ neuronal autophagy, improve neurological deficit and cognitive function; Cx43 activates P2X7R and ↓ GLT-1	[84]
16	in vivo (weight drop) + hypothermia	Rats	Weight drop	Cx43 + GLT-1	Mild induced hypothermia (33 °C, 4 h)	Hypothermia ↓ Cx43 and ↑ GLT-1 in hippocampus, reduces edema and neurological deficits	[85]
17	in vivo (needle stab wound)	WT and Cx43 KO mice	Needle stab wound	Cx43	Cx43 KO + observation	Cx43 ↑ in reactive astrocytes from 6 dpi, persists to 15 dpi; Cx43 KO enhances microgliosis and astrogliosis; Cx43 is a marker of astrogliosis	[86]
18	in vivo (TBI model) + siRNA	(Pediatric/adolescent TBI model)	TBI	Cx43	siRNA against Cx43	siRNA Cx43 ↓ astrogliosis, improves motor recovery at 1–3 days, but no effect on edema	[87]
19	in vivo (mild TBI/concussion)	WT and Cx43S368A mice	Mild TBI/concussion	Cx43 (S368 phosphorylation)	Cx43S368A mutant	After mild TBI ↑ p-Cx43 (S368) and hemichannels; S368A mutant ↓ seizure susceptibility (PTZ test)	[88]
20	in vitro + in vivo (TBI)	Astrocytes + neurons + rats	TBI model	Cx43 (GJA1-20K in exosomes)	GJA1-20K exosomes from astrocytes	GJA1-20K exosomes ↓ neuronal apoptosis, ↑ mitochondrial function and restore damaged neurons	[89]
21	in vivo (CCI)	Rats	CCI	Cx40	Observation + chloroquine (autophagy inhibitor)	Cx40 ↓ from day 2 to 6; coincides with ↑ autophagy (LC3-II, p62); chloroquine prevents Cx40 decrease	[90]
22	in vitro (OGD/R) + in vivo (TBI model)	Astrocytes; rats	TBI model	Cx43 (p-Cx43)	Remazolam	↓ Cx43/p-Cx43 and ROS, reduced astrocyte injury and brain edema; modulated A1/A2 polarization (↓ C3, ↑ S100A10)	[91]

Abbreviations: TBI—traumatic brain injury; FPI—fluid percussion injury; CCI—controlled cortical impact; NSC—neural stem cells; BMSC—bone marrow stromal cells; Cx43, Cx36, Cx40—connexins 43, 36 and 40, respectively; GJA1-20K—truncated isoform of connexin 43; p-Cx43—phosphorylated connexin 43; KO—knockout; AS-ODN—antisense oligodeoxynucleotide; siRNA—small interfering RNA; CBX—carbenoxolone; αCT1—connexin 43 mimetic peptide; miR-302—microRNA-302; ERK1/2—extracellular signal-regulated kinase 1/2; p-ERK—phosphorylated extracellular signal-regulated kinase; LC3, LC3-II—microtubule-associated protein 1 light chain 3; beclin-1—autophagy-related protein beclin-1; 3-MA—3-methyladenine; P2X7R—purinergic receptor P2X7; GLT-1—glutamate transporter 1; p62—sequestosome 1; NAC—N-acetylcysteine; GS-Rb1—ginsenoside Rb1; U0126—ERK pathway inhibitor; NEDD4—neural precursor cell expressed developmentally downregulated protein 4; H_2_—molecular hydrogen; PTZ—pentylenetetrazole seizure model; dpi—days post-injury.

**Table 4:** Role of Connexins in Spinal Cord Injury: Experimental Models, Modulation Approaches, and Key Results. Arrows indicate direction of change or relationship: ↑ increase; ↓ decrease.

No.	Experimental Model	Subject (Animals/Cells)	SCI Type	Connexin Isoform	Cx Modulation	Key Results	Reference
1	in vivo (height drop)	Mice with astrocyte-specific Cx30/Cx43 deletion and controls	Experimental SCI (drop)	Cx43 (hemichannels)	Astrocyte-specific Cx30/Cx43 deletion	Significant ↓ ATP release, ↓ astrogliosis and microglial activation, smaller lesion size and significant improvement in motor function	[10]
2	in vivo (MASCIS)	Rats	10 g weight drop from 12.5 mm at T10 level	Cx43	Peptide5 (Cx43 mimetic, intrathecal at 24 h)	Improved locomotion (BBB), ↓ Cx43, ↑ p-Cx43, ↓ TNF-α and IL-1β, ↓ astrocytosis, ↓ microglial activation, ↑ motor neuron survival at 5 weeks	[11]
3	in vivo (mild contusion)	Rats	Mild spinal cord contusion	Cx43	Systemic (i.p.) Peptide5 (immediately, 2 h and 4 h post-injury)	Significant improvement in hindlimb motor function (3–6 weeks), ↓ mechanical allodynia (1 and 6 weeks), ↓ total Cx43, ↑ p-Cx43 at 8 h, ↓ lesion size, ↓ gliosis and macrophage/microglial activation at 2 and 6 weeks, ↑ neuronal survival	[12]
4	in vivo (weight drop)	Cx43/Cx30 KO and control mice	SCI (weight drop)	Cx43	Cx43/Cx30 deletion	Complete prevention of thermal hyperalgesia and mechanical allodynia (develops at 4–8 weeks in WT); ↓ gliosis (GFAP)	[94]
5	in vivo (compression)	Rats	Compressive SCI	Cx43	Observation of Cx43 expression	Loss of Cx43 immunoreactivity at 1–3 days in neuron-depleted zones; complete absence of Cx43 at 7 days in epicenter; co-localization with GFAP in reactive astrocytes	[95]
6	in vivo (compression + partial transection)	Rats	Compressive SCI and partial transection	Cx43	asODN against Cx43 (intracerebroventricular)	↑ locomotion (sustained ≥4 weeks), ↓ edema, ↓ tissue damage, ↓ GFAP, ↓ neutrophil and albumin infiltration	[96]
7	in vivo (SCI model)	Rodents	Experimental SCI	Cx43 (hemichannels)	Peptide4 (Gap27) and Peptide5	Peptide5 (and Peptide4) significantly ↓ edema; Peptide5 ↓ Cx43, ↓ GFAP^+^ astrocytes, ↓ neuronal loss (NeuN, SMI-32)	[97]
8	in vivo (“infinite vertical impactor”)	Rats	SCI	Cx43	Gap27 (Cx43 inhibitor) + Fer-1	Gap27 ↓ ferroptosis (↑ GPX4, GSH; ↓ 4-HNE, MDA), ↑ SLC7A11, ↓ P-mTOR/mTOR; improves functional recovery (BBB, inclined plane)	[98]
9	in vivo (two models of incomplete SCI)	Mice	Incomplete SCI	Cx43 (hemichannels)	MHC1 (chimeric antibody against Cx43 hemichannels)	MHC1 ↓ hemichannel activation, ↓ gliosis, ↓ lesion size, ↑ white/gray matter preservation, ↑ neuronal survival, significant improvement in locomotion (up to 8 weeks)	[99]
10	in vivo (complete T8 transection) + HF-rTMS	Mice	Complete T8 transection	Cx43	HF-rTMS (15 Hz daily)	HF-rTMS ↓ Cx43, ↑ LC3II/P62 (autophagy activation), ↑ mTOR/p-mTOR/p-S6; improved BMS and motor function	[100]
11	in vivo (thoracic hemisection)	Rats	Thoracic hemisection	Cx43	Sig-1R blockade (BD1047) + Gap26	SCI ↑ Sig-1R and Cx43 in astrocytes; Sig-1R blockade ↓ Cx43, ↓ gliosis and mechanical allodynia	[101]
12	in vivo (peripheral nerve injury, neuropathic pain model)	Rats	Neuropathic pain model (SCI-related)	Cx36	siRNA Cx36 (intrathecal)	siRNA Cx36 ↓ Cx36 in dorsal horn, induces tactile allodynia; effect attenuated by MK-801 (NMDA antagonist)	[102]
13	in vivo (SCI model) + HBO	Rats	SCI	Cx43 + VEGF	Hyperbaric oxygen (2.0 ATA, 100% O_2_)	HBO improves BBB, ↓ histological damage, ↑ VEGF, ↓ Cx43 at 3–7 days, ↑ Cx43 at day 14	[103]
14–15	in vitro + in vivo (epSPC)	epSPC and epSPCi (ependymal spinal progenitor cells)	SCI	Cx50	Cx50 KO/overexpression + clotrimazole	Cx50 ↑ in epSPC (uninjured), ↓ in epSPCi; Cx50 promotes glial differentiation and regulates Sox2	[104,105]
16	in vivo (T9 transection + contusion)	mKO mice (conditional muscle Cx43/45 KO)	Complete T9 transection and T9 contusion	Cx43/Cx45 (muscle)	Conditional muscle Cx43/45 KO (mKO)	mKO ↓ muscle atrophy, improves locomotion (especially after incomplete injury); effect is sex-dependent	[106]
17	in vivo (suprasacral and sacral SCI)	Sprague-Dawley rats	Suprasacral (SSCI) and sacral (SCI) SCI	Cx43 + C-kit	Observation	SSCI ↑ Cx43 and C-kit + detrusor hyperreflexia; SCI ↓ Cx43 and C-kit + areflexia	[107]

Abbreviations: SCI—spinal cord injury; KO—knockout; Cx43, Cx30, Cx36, Cx45, Cx50—connexins 43, 30, 36, 45 and 50, respectively; ATP—adenosine triphosphate; GFAP—glial fibrillary acidic protein; TNF-α—tumor necrosis factor alpha; IL-1β—interleukin-1 beta; BBB—Basso, Beattie, Bresnahan locomotor scale; BMS—Basso Mouse Scale; asODN—antisense oligodeoxynucleotide; Gap26, Gap27, Peptide4, Peptide5—connexin mimetic peptide inhibitors; Fer-1—ferrostatin-1 (ferroptosis inhibitor); GPX4—glutathione peroxidase 4; GSH—glutathione; 4-HNE—4-hydroxynonenal; MDA—malondialdehyde; SLC7A11—cystine/glutamate antiporter subunit; mTOR—mechanistic target of rapamycin; P-mTOR—phosphorylated mTOR; p-S6—phosphorylated ribosomal protein S6; LC3-II—microtubule-associated protein 1 light chain 3 II; p62—sequestosome 1; MHC1—monoclonal antibody targeting Cx43 hemichannels; HF-rTMS—high-frequency repetitive transcranial magnetic stimulation; Sig-1R—sigma-1 receptor; BD1047—sigma-1 receptor antagonist; siRNA—small interfering RNA; MK-801—NMDA receptor antagonist; VEGF—vascular endothelial growth factor; HBO—hyperbaric oxygen; ATA—atmospheres absolute; epSPC—ependymal spinal progenitor cells; epSPCi—injury-activated ependymal spinal progenitor cells; Sox2—SRY-box transcription factor 2; mKO—muscle-specific connexin knockout; C-kit—stem cell factor receptor.

**Table 5:** Role of Connexins in Peripheral Nerve Injury: Experimental Models, Modulation Approaches, and Key Results. Arrows indicate direction of change or relationship: ↑ increase; ↓ decrease; → indicates effect or association.

No.	Experimental Model	Subject (Animals/Cells)	Injury Type	Connexin Isoform	Cx Modulation	Key Results	Reference
1	in vivo (genetic knockout)	cx32-null mice (males cx32−/Y and females cx32−/−)	Hereditary demyelinating neuropathy (CMTX model)	Cx32	Complete Cx32 knockout	Progressive demyelinating peripheral neuropathy from 3 months; motor fibers more affected than sensory; heterozygous females (random inactivation) show less demyelination than homozygotes; no myelin abnormalities in CNS	[15]
2	in vivo (CCI of sciatic nerve)	Rats	Chronic constriction injury of sciatic nerve (CCI)	Cx43 + miR-1/BDNF	Observation of ↓ miR-1	CCI → time-dependent ↓ miR-1 in sciatic nerve, ↑ BDNF and Cx43 in nerve and DRG; Cx43 appears in endoneurium of injured nerves	[109]
3	in vivo (inferior alveolar nerve injury)	Rats	Inferior alveolar nerve injury	Cx43	Gap27 (Cx43 inhibitor in trigeminal ganglion)	Injury → prolonged mechanical allodynia of whisker pad and eyelid skin + satellite glial cell activation; ↑ Cx43 in satellite glial cells of trigeminal ganglion; Gap27 ↓ glial activation and allodynia	[110]
4	in vivo (CCI + CIPN models)	Mice	Peripheral nerve injury (CCI) and chemotherapy-induced peripheral neuropathy (oxaliplatin/paclitaxel)	Cx43 (hemichannels)	Peptide5 (intrathecal)	CCI → ↑ Cx43 in L3–L5 astrocytes at day 10; Peptide5 ↓ mechanical hypersensitivity, ↓ Cx43, ↓ microglia/astrocytes, ↓ NLRP3 inflammasome (ASC, caspase-1); no effect in CIPN	[111]
5	in vivo (transection + delayed neurorrhaphy)	Rats	Common peroneal nerve transection + delayed neurorrhaphy	Cx43/Cx45 (muscle hemichannels)	Boldine (oral hemichannel inhibitor)	Boldine ↑ evoked response in tibialis anterior muscle at 2 weeks, ↓ fiber atrophy at 4 weeks, ↑ electrophysiological activity and muscle maturation at 6 weeks post-neurorrhaphy	[112]
6	in vivo (PSNL)	Mice	Partial sciatic nerve ligation (PSNL)	Cx43 (dorsal horn astrocytes)	siRNA Cx43/Ad-Cx43 (intrathecal)	PSNL → ↓ Cx43 from day 7 (maintenance, not initiation of pain); siRNA Cx43 induces hypersensitivity; Ad-Cx43 attenuates it; effect via ↓ GLT-1 and ↑ glutamatergic transmission (MK801/CNQX attenuate)	[113]
7	in vitro (astrocytes) + in vivo (PSNL)	Astrocyte cultures + mice	Partial sciatic nerve ligation (PSNL)	Cx43	Lycopene (repeated intrathecal)	Lycopene (in vitro) reverses TNF-α-induced ↓ Cx43; in vivo repeated administration ↑ Cx43, ↓ mechanical hypersensitivity	[114]
8	in vivo (SNL L5)	Rats	L5 spinal nerve ligation (SNL)	Cx43	siRNA Cx43 (intrathecal)	siRNA Cx43 ↓ mechanical hypersensitivity; correlates with ↓ Cx43 (not Cx36/GFAP) in spinal cord	[115]
9	in vivo (transgenic mice)	Transgenic mice (T55I or R75W + IRES-EGFP under Cnp promoter)	CMTX model (Cx32 mutations)	Cx32 (T55I, R75W)	Transgenic expression of mutants	Mutants localize perinuclearly and fail to form plaques; cause demyelinating peripheral neuropathy and CNS myelination defects (R75W stronger)	[116]
10	in vivo (sciatic nerve compression)	Rats	Sciatic nerve compression	Cx26, Cx32, Cx43	Observation	Cx32 in paranodal regions; Cx26 and Cx43 in perineurium; after compression Cx32 temporarily disappears then recovers; Cx43 rapidly appears in endoneurium	[117]
11	in vivo (ob/ob)	ob/ob mice	Diabetic peripheral neuropathy	Cx32, Cx26, Cx43	COMP-Ang-1 (intraperitoneal days 7–21)	COMP-Ang-1 ↑ Nf68/GAP43, ↑ Cx32/Cx26, ↓ Cx43/TNFα, ↓ macrophage/T-cell infiltration, improves microvascular regeneration	[118]
12	in vivo (Cx26 CKO)	Cx26 CKO mice (under Sox2 promoter)	GJB2-associated deafness model	Cx26	Conditional Cx26 knockout in cochlear supporting cells	Severe hearing loss, loss of hair cells and Deiters’ cells, abnormal innervation, demyelination, degeneration of spiral ganglion neurons	[119]
13	in vivo (sciatic nerve injury)	Rats	Sciatic nerve injury	Cx36	Observation	Cx36 expressed in neurons and satellite glial cells of DRG; after injury ↓ Cx36 mRNA in L4 DRG	[120]
14	in vivo (oxaliplatin)	Cx36 KO, Het and WT mice	Chemotherapy-induced peripheral neuropathy (oxaliplatin)	Cx36	Complete Cx36 knockout	Oxaliplatin induces tactile hypersensitivity in WT; Cx36 KO significantly attenuates it	[121]
15	in vivo (pT-ION)	Mice	Partial transection of infraorbital nerve (pT-ION)	Cx36	Mefloquine (Cx36 inhibitor)	pT-ION ↑ Cx36, GluK2, TRPA1, p-ERK in trigeminal ganglion; mefloquine more strongly attenuates cold allodynia than mechanical	[122]
16	in vivo (axotomy)	Adult cats	Nerve transection	Cx36, Cx37, Cx40, Cx43, Cx45	Observation + dye coupling	After axotomy motoneurons regain dye coupling; connexin expression is maintained	[123]
17	in vitro (mixed cultures) + ex vivo (DRG neurons) + in vivo (mice)	HEK-293T (NMNAT overexpression), DRG neurons, mice (AAV-PHP.eB Cx36 KD)	Axotomy, CZ-48, neuroinflammation	Cx43 (HEK), Cx36 (neurons)	GJIC (via Cx43/Cx36), Cx36 inhibition	High NAD inhibits SARM1 not only in its own cell but in 5–10 neighboring cells via Cx43-GJIC (visualized by PC11 → PAD11 probe). In neurons Cx36 mediates protective signal transfer from healthy to axotomized axons. Cx36 knockdown in vivo → neuroinflammation, SARM1 activation, axon degeneration and behavioral deficits. Healthy neurons protect injured axons via GJIC	[125]
18	in vivo (sciatic nerve)	Rats	Normal and compression	Cx29, Cx32	Observation	Cx29 in inner myelin layer, paranode, mesaxon; colocalizes with Kv1.2; Cx32 in outer layers	[126]
19	in vivo (CNS/PNS myelin)	Mice and rats	Normal	Cx29	Observation	Cx29 in internodal and paranodal regions of small CNS myelin sheaths; in PNS precedes Cx32	[127]
20	in vivo (sciatic nerve injury)	Rats	Sciatic nerve injury	Cx37	Observation of ↑ Cx37 mRNA	↑ Cx37 mRNA proximal/distal in sciatic nerve at 7–14 days; correlates with thermal hyperalgesia	[128]
21	in vivo (TMJ inflammation)	Rats	Capsaicin or CFA injection into TMJ	Cx26, Cx36, Cx40, Cx43	Observation	↑ Cx26, Cx36, Cx40 in trigeminal ganglion; Cx43 unchanged; formation of Cx26 plaques between neurons and satellite glial cells	[129]

Abbreviations: CMTX—X-linked Charcot–Marie–Tooth disease; CCI—chronic constriction injury; CIPN—chemotherapy-induced peripheral neuropathy; PSNL—partial sciatic nerve ligation; SNL—spinal nerve ligation; pT-ION—partial transection of infraorbital nerve; TMJ—temporomandibular joint; DRG—dorsal root ganglion; CNS—central nervous system; PNS—peripheral nervous system; KO—knockout; WT—wild type; Het—heterozygous; CKO—conditional knockout; Cx26, Cx29, Cx32, Cx36, Cx37, Cx40, Cx43, Cx45—connexins 26, 29, 32, 36, 37, 40, 43 and 45, respectively; miR-1—microRNA-1; BDNF—brain-derived neurotrophic factor; TNF-α—tumor necrosis factor alpha; NLRP3—NLR family pyrin domain containing 3 inflammasome; ASC—apoptosis-associated speck-like protein containing CARD; GLT-1—glutamate transporter 1 (EAAT2); MK-801—NMDA receptor antagonist; CNQX—AMPA receptor antagonist; TRPA1—transient receptor potential ankyrin 1; GluK2—kainate receptor subunit 2; ERK—extracellular signal-regulated kinase; EGFP—enhanced green fluorescent protein; Cnp—2′,3′-cyclic nucleotide 3′ phosphodiesterase promoter; COMP-Ang-1—cartilage oligomeric matrix protein-angiopoietin-1; Nf68—neurofilament 68 kDa; GAP43—growth associated protein 43; CFA—complete Freund’s adjuvant; Kv1.2—voltage-gated potassium channel subunit 1.2; HEK-293T—human embryonic kidney 293T cells; NMNAT—nicotinamide mononucleotide adenylyltransferase; NAD—nicotinamide adenine dinucleotide; SARM1—sterile alpha and TIR motif containing 1; AAV-PHP.eB—adeno-associated virus PHP.eB serotype; GJIC—gap junction intercellular communication.

**Table 6:** Role of Connexins in Alzheimer’s Disease: Experimental Models, Modulation Approaches, and Key Results. Arrows indicate direction of change or relationship: ↑ increase; ↓ decrease; → indicates effect or association.

No.	Experimental Model	Subject (Animals/Cells)	AD Model Type	Connexin Isoform	Cx Modulation	Key Results	Reference
1	in vivo (5XFAD)	5XFAD mice (cortex and thalamus)	5XFAD (amyloid AD model)	Cx43, Cx30, Cx47	Observation	In aged 5XFAD ↑ Cx43 and Cx30 (astrogliosis), ↓ Cx47 and Cx47/Cx43 co-localization; oligodendrocyte depletion and myelin deficit	[18]
2	in vitro + ex vivo (acute hippocampal slices)	Astrocytes + APP/PS1 hippocampal slices	APPswe/PS1dE9	Cx43 + Pannexin 1 (hemichannels)	Observation of hemichannel activation	Aβ plaques activate Cx43 hemichannels (and Pannexin 1 in reactive astrocytes); ATP/glutamate release → vicious Ca^2+^ cycle; Cx43 knockout ↓ gliotransmitter release and neuronal damage	[21]
3	in vivo (5xFAD, spinal cord)	5xFAD mice (12 months)	5xFAD	Cx43, Cx30, Cx47, Cx32	Observation	↑ Cx43/Cx30 around plaques, ↑ Cx47 in oligodendrocytes, ↓ Cx32; focal myelin deficit with preserved axons	[131]
4	Postmortem + in vitro	Human brain + cultures	PD	Cx43 (microglial hemichannels)	TAT-Cx43@LNPs (hemichannel blocker)	↑ Cx43 in perilesional microglia; blockade of microglial Cx43 hemichannels → neuroprotective phenotype, ↓ neurotoxicity, slowing of disease progression	[132]
5	in vitro (primary astrocytes)	Primary mouse astrocytes + Aβ25-35	Aβ-induced model	Cx43	Aβ25-35 treatment	Aβ disrupts functional gap junctions, ↑ Cx43 hemichannel activity; ↑ intracellular Cx43 pool and ER retention; 4-phenylbutyrate restores trafficking	[133]
6	in vivo (APP/PS1)	APP/PS1 mice (≥4 months)	APP/PS1	Cx43 and Cx30	Observation	Local ↑ Cx43/Cx30 around Aβ plaques (astroglial processes); overall ↑ expression in 18-month-old mice	[134]
7	in vivo (APP/PS1 + astrocyte Cx43 KO)	APP/PS1/Gfap-Cx43 KO mice (12 months)	APP/PS1	Cx43 (astrocytes)	Astrocyte-specific Cx43 KO	Cx43 KO improves cognitive function, ↓ astrogliosis (GFAP), ↑ synapses; no effect on Aβ plaques; ↓ astrogliosis activation modulators	[135]
8	ex vivo (postmortem human brain)	Human AD brain	Sporadic AD	Cx43	Observation	↑ Cx43 immunoreactivity in cortical areas with Aβ plaques; Cx43 in astrocytic gap junctions around plaques	[136]
9	in silico + in vitro + ex vivo (human data)	Human AD transcriptome/proteome + Gja1−/− astrocytes	Human AD + Gja1 KO	GJA1 (Cx43)	Gja1 KO in astrocytes	GJA1 ↑ in AD, correlates with Aβ/tau and cognitive deficit; KO ↓ Apoe, ↓ Aβ phagocytosis, ↑ neuroprotection	[137]
10	in vivo (APPswe/PS1dE9)	APPswe/PS1dE9 mice (9 months)	APPswe/PS1dE9	Cx43	Observation of ↑ Cx43	↑ Cx43 and MFN2 (MAM); Cx43 ↑ MAM → autophagy inhibition (LC3B-I → LC3B-II) → ↓ Aβ clearance	[138]
11	in vivo + in vitro (NVU/BBB)	in vivo AD model + in vitro BBB + astrocytes	Aβ toxicity	Cx43 + CD38	Observation	Aβ ↑ CD38- and Cx43-positive cells in NVU and BBB; ↑ Cx43 hemichannel permeability	[139]
12	in vitro (primary astrocytes)	Primary astrocytes + Aβ1-42	Aβ1-42	Cx43 (hemichannels) + A2AR	Aβ1-42 + A2AR modulation	Aβ → ↑ Cx43 hemichannels via A2AR → ↑ ATP → adenosine (CD73) → positive feedback; A2AR directly binds Cx43	[140]
13	in vivo + ex vivo (APP/PS1 and Aβ models)	APP/PS1 mice + hippocampal slices + Aβ1-42	APP/PS1 and icv Aβ1-42	Cx43 (hemichannels) + A2AR	Pharmacological/genetic A2AR blockade	Aβ ↑ Cx43 hemichannel activity (early event); A2AR (PKC pathway) regulates hemichannels; A2AR blockade prevents dysregulation	[141]

Abbreviations: AD—Alzheimer’s disease; Aβ—amyloid beta peptide; APP/PS1, APPswe/PS1dE9—transgenic amyloid AD mouse models; 5xFAD—five familial Alzheimer’s disease mutation mouse model; KO—knockout; Gja1—gene encoding connexin 43; Cx30, Cx32, Cx43, Cx47—connexins 30, 32, 43 and 47, respectively; Pannexin 1—pannexin 1 channel protein; ATP—adenosine triphosphate; Ca^2+^—calcium ion; GFAP—glial fibrillary acidic protein; NVU—neurovascular unit; BBB—blood–brain barrier; CD38—cluster of differentiation 38 ectoenzyme; CD73—ecto-5′-nucleotidase; A2AR—adenosine A2A receptor; PKC—protein kinase C; Apoe—apolipoprotein E; MFN2—mitofusin 2; MAM—mitochondria-associated membranes; LC3B—microtubule-associated protein 1 light chain 3B (autophagy marker); icv—intracerebroventricular administration.

**Table 7:** Role of Connexins in Parkinson’s Disease: Experimental Models, Modulation Approaches, and Key Results. Arrows indicate direction of change or relationship: ↑ increase; ↓ decrease.

No.	Experimental Model	Subject (Animals/Cells)	PD Model Type	Connexin Isoform	Cx Modulation	Key Results	Reference
1	in vivo (LPS)	C57BL/6 mice	LPS-induced PD model	Cx43	Gap27 (Cx43 inhibitor)	LPS ↓ total Cx43 (~60%), ↑ p-Cx43 (2-fold); Gap27 ↓ loss of dopaminergic neurons, restores dopamine levels, ↓ microgliosis/astrogliosis and inflammation, ↑ neurotrophic factors	[22]
2	Postmortem brain analysis	Human brain (cortex and basal ganglia)	Sporadic late-stage PD	Cx43	Observation of ↓ Cx43	Significant loss of Cx43 in late-stage PD (especially in specific areas); correlates with non-motor symptoms (depression, sleep disturbances); simplification of astrocytic arborization	[143]
3	in vivo + in vitro (rotenone)	Rats + astrocyte cultures	Rotenone model of PD	Cx43	Observation of ↑ Cx43	Rotenone ↑ Cx43 and p-Cx43 in astrocytes (in vivo and in vitro), ↑ GJIC; selectively in basal ganglia (SN, ST, GPe, GPi)	[144]
4	in vivo (rotenone) + gastrodin	Rats	Rotenone model of PD	Cx43	Gastrodin (↓ Cx43)	Gastrodin ↓ Cx43 phosphorylation and GJIC; prevents development of PD	[145]
5	in vivo (6-OHDA)	C57BL/6 mice	6-OHDA model of PD	Cx43	Gap27 (mimetic peptide)	6-OHDA ↑ Cx43, ↓ p-Cx43 (S368); Gap27 ↓ death of dopaminergic neurons, normalizes Cx43/pS368	[146]
6	in vivo (MPTP)	WT and Cx30 KO mice	MPTP model of PD	Cx30	Cx30 KO	MPTP ↑ Cx30; Cx30 KO accelerates DA neuron loss, weakens astrogliosis, ↓ GDNF and S100a10 in astrocytes	[147]
7	in vivo (MPTP) + FGF-2	Rats	MPTP model of PD	Cx43	FGF-2 (implantation)	MPTP ↑ Cx43; FGF-2 further ↑ Cx43-positive puncta; changes in astroglial cells	[148]
8	in vivo (6-OHDA)	Rats	6-OHDA model of PD	Cx36	Observation	↑ Cx36 in striatum and motor cortex; ↑ in ENK+ striatal neurons, ↓ in PV+ interneurons	[149]
9	in vivo (LID model)	Rats	Levodopa-induced dyskinesia (LID)	Cx36	Carbenoxolone/quinine (GJ blockade)	↑ Cx36 in striatum and motor cortex (ENK+ and PV+ neurons); GJ blockade ↓ AIMs	[150]
10	in vivo (6-OHDA) + Baicalin	Rats	6-OHDA model of PD	Cx36	Baicalin	6-OHDA ↓ Cx36 in cortex and striatum; Baicalin ↑ Cx36 and TH, improves symptoms	[151]
11	in vivo + in vitro (α-syn)	Cells + mice (PD/MSA models)	α-synucleinopathies	Cx32	Observation + Cx32 blockade	Cx32 mediates uptake of oligomeric α-syn by neurons and oligodendrocytes; ↑ Cx32 in PD/MSA	[152]
12	Postmortem human brain analysis	Human brain (striatum, GPe/GPi, STN)	Sporadic PD	Cx36	Observation of ↑ Cx36	↑ Cx36 in GPe/GPi and striatum in PD (+50–109%); role of GJ in basal ganglia synchrony	[153]
13	in vitro (MPP+)	SH-SY5Y cells	MPP+-induced model	Cx26 (GJB2)	Observation	MPP+ alters EGFR pathway gene expression, including GJB2 (Cx26); potential role in DA neuron apoptosis	[154]

Abbreviations: PD—Parkinson’s disease; LPS—lipopolysaccharide; 6-OHDA—6-hydroxydopamine; MPTP—1-methyl-4-phenyl-1,2,3,6-tetrahydropyridine; MPP^+^—1-methyl-4-phenylpyridinium; LID—levodopa-induced dyskinesia; AIMs—abnormal involuntary movements; KO—knockout; WT—wild type; Cx26, Cx30, Cx32, Cx36, Cx43—connexins 26, 30, 32, 36 and 43, respectively; GJB2—gene encoding connexin 26; GJ—gap junctions; GJIC—gap junction intercellular communication; SN—substantia nigra; ST—striatum; GPe—globus pallidus externa; GPi—globus pallidus interna; STN—subthalamic nucleus; DA—dopaminergic; TH—tyrosine hydroxylase; ENK—enkephalin-positive neurons; PV—parvalbumin-positive interneurons; α-syn—alpha-synuclein; MSA—multiple system atrophy; FGF-2—fibroblast growth factor 2; GDNF—glial cell line-derived neurotrophic factor; S100a10—S100 calcium-binding protein A10; EGFR—epidermal growth factor receptor; LNPs—lipid nanoparticles.

**Table 8:** Role of Connexins in Amyotrophic Lateral Sclerosis: Experimental Models, Modulation Approaches, and Key Results. Arrows indicate direction of change or relationship: ↑ increase; ↓ decrease; → indicates effect or association.

No.	Experimental Model	Subject (Animals/Cells)	ALS Model Type	Connexin Isoform	Cx Modulation	Key Results	Reference
1	in vivo (mSOD1-Tg)	mSOD1-Tg and non-Tg mice (lumbar spinal cord)	mSOD1 transgenic ALS model	Cx43, Cx30 (astrocytes), Cx47, Cx32 (oligodendrocytes)	Observation	At progressive and terminal stages ↑ Cx43/Cx30 and GFAP/AQP4 (astrogliosis), ↓ Cx47/Cx32 in oligodendrocytes (especially with SOD1 accumulation), ↓ EAAT2; neuronal loss and microglial activation	[19]
2	in vivo + in vitro (hiPSC)	SOD1G93A mice + hiPSC astrocytes and MN	SOD1G93A + hiPSC ALS model	Cx43	Cx43 knockout/blockade (pan- and hemichannel)	↑ Cx43 and hemichannels in SOD1G93A astrocytes; ↑ GJIC, ↑ intracellular Ca^2+^; Cx43 blockade ↓ gliotransmitter release and protects motor neurons	[23]
3,4	in vivo (SOD1G93A) + hiPSC	SOD1G93A mice + hiPSC astrocytes/MN (familial and sporadic ALS)	SOD1G93A + hiPSC ALS model	Cx43 (hemichannels)	Astrocyte-specific Cx43 KO + Gap27/tonabersat	Astrocyte-specific Cx43 KO slows disease progression, protects MN and improves survival; Gap27/tonabersat block hemichannels → neuroprotection and ↓ MN excitability	[155,156]
5	Postmortem analysis + transcriptomics	Human spinal cord + GJA1 stratification	Sporadic ALS	GJA1 (Cx43)	Observation of ↑ GJA1	↑ GJA1 in motor neurons in ALS; positive correlation with microglial activation, negative with neuronal; GJA1 is a novel neuroimmune gene	[157]
6	Postmortem analysis	Human spinal cord	Sporadic ALS	Cx43	Observation of ↑ Cx43	↑ Cx43 in astrocytes surrounding depleted motor neurons; suggested role in reactive glia and neurotoxicity	[158]
7	in vivo (mSOD1 + Cx30 KO)	mSOD1 + Cx30 KO mice	mSOD1 ALS model	Cx30	Cx30 KO	Cx30 ↑ at presymptomatic stage; Cx30 KO delays ALS onset, preserves MN, ↓ inflammatory astrogliosis and Cx43	[159]
8	in vitro + ex vivo (spinal cord)	MN cultures + ALS tissues	ALS (human and SOD1G93A)	Cx36	Cx36 knockdown	Cx36 ↓ in spinal cord in ALS; Cx36 knockdown ↓ mSOD1-induced MN death	[160]
9	in silico (molecular docking)	Cx31/Cx43 models	ALS (hypothetical link with T2D)	Cx31, Cx43	Insulin (docking)	Insulin blocks Cx31/Cx43 pores; explains protective effect of T2D (hyperinsulinemia) in ALS	[161]

Abbreviations: ALS—amyotrophic lateral sclerosis; mSOD1—mutant superoxide dismutase 1; SOD1G93A—glycine-to-alanine substitution at position 93 of SOD1; hiPSC—human induced pluripotent stem cells; MN—motor neurons; KO—knockout; Cx30, Cx31, Cx32, Cx36, Cx43, Cx47—connexins 30, 31, 32, 36, 43 and 47, respectively; GJA1—gene encoding connexin 43; GJIC—gap junction intercellular communication; GFAP—glial fibrillary acidic protein; AQP4—aquaporin 4; EAAT2—excitatory amino acid transporter 2; Ca^2+^—calcium ion; T2D—type 2 diabetes mellitus.

**Table 9:** Role of Connexins in Huntington’s Disease: Experimental Models, Modulation Approaches, and Key Results. Arrows indicate direction of change or relationship: ↑ increase; ↓ decrease.

No.	Experimental Model	Subject (Animals/Cells)	HD Model Type	Connexin Isoform	Cx Modulation	Key Results	Reference
1	Postmortem immunohistochemical analysis	Human brain (caudate nucleus and globus pallidus)	Sporadic HD (human)	Cx26, Cx32, Cx40, Cx43, Cx50	Observation	Cx43 ↑ in astrocytes and neurons surrounding degenerating neurons; ↑ reactive astrocytosis (GFAP); Cx26/Cx32/Cx40/Cx50—no significant changes; suggested enhancement of astrocytic gap junctions for spatial buffering	[24]
2	in vivo (transgenic mice)	R6/2 mice	Transgenic HD model (R6/2)	Cx36, Cx45 (retina)	Observation of ↓ Cx36/Cx45	Retinal degeneration with photoreceptor apoptosis; ↓ Cx36 in the outer plexiform layer and ↓ Cx45 in the inner plexiform layer; mutant huntingtin and ubiquitin expressed in neurons and glia	[162]
3	Postmortem brain analysis	Human brain (middle cingulate cortex)	Sporadic HD (human)	Cx43 (astrocytes)	Observation of ↑ Cx43	↑ mutant huntingtin density (1C2) + ↑ activation of microglia (Iba1, HLA-DP/DQ/DR) and astrocytes (Cx43, GFAP, EAAT2) in the middle cingulate cortex; correlates with mood symptoms	[163]

Abbreviations: HD—Huntington’s disease; R6/2—transgenic Huntington’s disease mouse model expressing mutant huntingtin; Cx26, Cx32, Cx36, Cx40, Cx43, Cx45, Cx50—connexins 26, 32, 36, 40, 43, 45 and 50, respectively; GFAP—glial fibrillary acidic protein; EAAT2—excitatory amino acid transporter 2; Iba1—ionized calcium-binding adapter molecule 1; HLA-DP/DQ/DR—major histocompatibility complex class II microglial activation marker; 1C2—antibody detecting mutant huntingtin protein (polyglutamine expansion); IHC—immunohistochemistry; FRAP—fluorescence recovery after photobleaching.

**Table 10:** Role of Connexins in Depression: Experimental Models, Modulation Approaches, and Key Results. Arrows indicate direction of change or relationship: ↑ increase; ↓ decrease; → indicates effect or association.

No.	Experimental Model	Subject (Animals/Cells)	Depression Model Type	Connexin Isoform	Cx Modulation	Key Results	Reference
1	in vivo (CSS) + astrocyte isolation	Mice (mPFC, hippocampus, amygdala, VTA)	Chronic social stress	Cx30, Cx43	Observation + Cx30/Cx43 overexpression/suppression	CSS → ↓ Cx30/Cx43 in mPFC and hippocampus, correlates with reduced neuronal activity; ↑ Cx30/Cx43 in these regions suppresses depressive behavior, while suppression of Cx30/Cx43 induces it	[26]
2	in vivo (Gap27 + LPS)	Mice	LPS-induced (inflammatory) model + Gap27 in PFC	Cx43	Gap27 (Cx43 inhibitor) + LPS	Cx43 blockade in PFC + LPS → depressive behavior (↓ sucrose preference, ↑ immobility time); ↑ peripheral inflammatory cytokines; LPS ↓ Cx43 and GJIC in PFC	[165]
3	in vitro + in vivo (Rg1)	Primary astrocytes + CUS rats	CUS + Rg1	Cx43	Rg1 (↑ Cx43) + Gap26/CBX	Rg1 ↑ Cx43 and GJIC, improves antidepressant-like behavior; effect abolished by Gap26/CBX	[166]
4	in vivo (CUS) + Cx43 KO	Mice (astrocyte-specific Cx43 KO in PFC)	CUS	Cx43	Astrocyte-specific Cx43 KO	Cx43 KO activates JAK2-STAT3 → ↑ TSPO and depressive behavior; CORT-induced GJ dysfunction and ↑ TSPO abolished by JAK2-STAT3 inhibitor	[167]
5	in vivo (LPS in PFC) + celecoxib	Rats	LPS-induced model (in PFC)	Cx43	Celecoxib (restores Cx43)	LPS → depressive behavior + ↓ Cx43/GJIC; celecoxib improves behavior, DMN rsFC, ↑ LY diffusion, normalizes p-Cx43 via NF-κB/p65	[168]
6	in vivo (CUS) + Rg1	Rats	CUS	Cx43	Rg1 (↓ Cx43 ubiquitination)	Rg1 ↓ Cx43 ubiquitination, attenuates neuroinflammation and depressive behavior	[169]
7	in vitro (Rg1 + CORT)	Primary rat astrocytes (prefrontal cortex and hippocampus)	CORT-induced stress in vitro	Cx43	Rg1 (↑ Cx43 mRNA, ↓ Cx43 degradation via ubiquitin-proteasome and autophagic-lysosomal pathways; improves GJIC)	Pretreatment with Rg1 prevents CORT-induced reduction in Cx43 biosynthesis, suppresses its degradation via ubiquitin-proteasome and autophagic-lysosomal pathways and restores Cx43-dependent gap junction function in astrocytes	[170]
8	Postmortem brain analysis	Human brain (neocortex, cerebellar cortex, thalamus, caudate nucleus)	Sporadic MDD (suicide victims)	CX30, CX43	Observation of ↓ CX30/CX43	Widespread reduction in CX30/CX43 across all regions (except cerebellum); ↑ H3K9me3 (epigenetic repression) in PFC	[171]
9	in vivo (CUMS) + HMGB1	Mice	CUMS	Cx36	Glycyrrhizic acid/quinine (↓ Cx36)	CUMS ↑ Cx36 in hippocampal neurons + ↑ HMGB1/TNF-α/IL-1β; Cx36 inhibitors attenuate depressive behavior and inflammation	[172]
10	Clinical (case–control)	76 patients with CID + 32 healthy controls (human serum)	Chronic insomnia (CID)	CX30, CX43 (and AQP4)	Observation (↓ levels)	Significant reduction in serum AQP4, CX30 and CX43 in CID patients. Positive correlation of AQP4, CX30 and CX43 with percentage and total slow-wave sleep time. Positive correlation of AQP4 and CX30 with MoCA-C score (cognitive function); negative correlation of AQP4 with spatial working memory errors (Nine Box Maze). Indicates astrocyte dysfunction linked to sleep quality impairment and cognitive deficits	[173]
11	Postmortem brain analysis	Orbitofrontal cortex (OFC): 23 MDD patients, 16 with alcohol dependence, 13 with MDD + alcoholism comorbidity, 20 healthy controls	Severe depressive disorder (MDD), alcoholism, comorbidity	Cx43	Observation (↓ level and immunoreactivity)	>60% reduction in Cx43 protein level (Western blot) in all three psychiatric groups vs. controls. ↓ immunoreactivity area and size of Cx43-immunoreactive puncta in all groups. ↓ density of immunoreactive puncta only in alcoholism. No differences between suicide and non-suicide victims. OFC dysfunction involves altered Cx43 gap junctions and/or hemichannels in the pathophysiology of depression and alcoholism	[174]
12	in vivo (genetic knockdown + chronic CORT ± fluoxetine)	Mice (hippocampus), constitutive Cx43 KD + wild-type	Chronic corticosterone (CORT) + fluoxetine	Cx43 (hippocampus)	Constitutive Cx43 knockdown; CORT ↑ p-Cx43; fluoxetine normalizes p-Cx43 and reduces expression/activity	Constitutive Cx43 deficiency produces antidepressant-like/anxiolytic behavior + improved cognitive function. CORT induces anxiety/depression, reversed by chronic fluoxetine. Antidepressants act partly by reducing Cx43 expression and/or phosphorylation in the hippocampus	[175]
13	in vivo (genetic and pharmacological Cx43 inactivation)	C57BL/6 mice (Cx43 KD) + outbred Swiss mice (shRNA-Cx43, CBX)	Depression/anxiety model (tail suspension test, elevated plus maze)	Cx43 (astrocytes, hippocampus + amygdala)	Constitutive Cx43 KD, shRNA-Cx43 (hippocampus/amygdala), CBX (10 mg/kg)	Cx43 inactivation enhances fluoxetine antidepressant effect (↑ extracellular 5-HT in hippocampus, stronger effect in tail suspension test). shRNA-Cx43 or CBX in hippocampus potentiates fluoxetine antidepressant action. Cx43 inactivation in amygdala (but not hippocampus) attenuates diazepam anxiolytic effect in elevated plus maze. Chronic CBX reproduces the effects	[176]
14	in vitro	Human astrocytoma cells (human astrocytic cells)	Study of acute fluoxetine effect on astrocytes in the context of depression	Cx43	Fluoxetine: ↑ Cx43	Acute fluoxetine increases Cx43 expression in astrocytes without changing AQP4, partly via cAMP-dependent pathway and independently of serotonin	[177]
15	in vivo (SNI)	Mice	Neuropathic pain (SNI)	Cx43	Observation + amitriptyline	SNI ↓ Cx43 in hippocampus; amitriptyline improves behavior and normalizes Cx43	[178]
16	in vivo (CUS) + Rg1	Rats	CUS	Cx43	Rg1 (Cx43–YAP)	Rg1 ↑ cytoplasmic Cx43–YAP interaction, ↓ nuclear YAP translocation → antidepressant effect	[179]
17	in vivo (CUS) + Rg1	Rats	CUS	Cx43	Rg1 (↑ Cx43 in hippocampus)	Rg1 improves astrocyte GJIC in hippocampus, attenuates depressive behavior	[180]
18	in vivo (CUS) + Rg1	Rats	CUS	Cx43	Rg1 (↑ Cx43)	Rg1 ↑ Cx43 in PFC and hippocampus, improves antidepressant-like behavior	[181]
19–20	in vivo (Mahonia alkaloids)	Rats (reserpine model) + astrocytes (CORT)	Reserpine + CORT	Cx43	Mahonia alkaloids (↓ miR-205 → ↑ Cx43)	Alkaloids ↓ miR-205, ↑ Cx43, improve behavior and neurotrophic factors	[182,183]
21	in vivo (loganin from Cornus)	CUS rats + primary astrocytes	CUS + CORT	Cx43	Loganin (↑ Cx43)	Loganin ↑ Cx43, improves behavior; effect abolished by Gap26	[184]
22	in vivo (CUS) + genistein	Mice	CUS	Cx43	Genistein (↓ miR-221/222 → ↑ Cx43)	Genistein ↓ miR-221/222, ↑ Cx43, improves behavior	[185]
23	in vivo (CUS) + hypericin	Rats	CUS	Cx43	Hypericin (restores GJ)	Hypericin normalizes GJ ultrastructure; effect abolished by CBX	[186]
24	in vivo (CRS + KRG)	Rats (prelimbic cortex)	Chronic restraint stress (CRS, 8 h/day, 28 days)	Cx43	KRG (25–100 mg/kg i.g.) ± CBX (GJIC blocker)	CRS induces depressive symptoms + GJIC dysfunction. KRG (dose-dependently) improves behavior (↓ immobility in FST, ↑ sucrose preference), ↑ LY diffusion and ↑ Cx43 in PLC/PFC. CBX induces depression and ↓ GJIC; KRG fully reverses CBX effects, proving that KRG antidepressant action is mediated by improvement of astrocytic gap junction function (not only astrocyte number)	[187]
25	in vivo (LPS/CUS) + D4	Mice	LPS + CUS	Cx (hemichannels)	D4 (selective Cx hemichannel inhibitor)	D4 ↓ hemichannel activity, neuroinflammation and depressive behavior; normalizes c-Fos in hippocampus, entorhinal cortex, etc.	[188]
26	in vivo (maternal separation) + Cx43	Mice (neonatal MS)	Early-life stress increasing depression risk	Cx43	Cx43 overexpression	MS → cognitive impairment + astrocyte dysfunction; ↑ Cx43 attenuates them	[189]

Abbreviations: MDD—major depressive disorder; CSS—chronic social stress; CUS/CUMS—chronic unpredictable stress (chronic unpredictable mild stress); CRS—chronic restraint stress; MS—maternal separation; SNI—spared nerve injury; LPS—lipopolysaccharide; CORT—corticosterone; CID—chronic insomnia disorder; PFC—prefrontal cortex; mPFC—medial prefrontal cortex; OFC—orbitofrontal cortex; VTA—ventral tegmental area; PLC—prelimbic cortex; Cx30, Cx36, Cx43—connexins 30, 36 and 43, respectively; GJIC—gap junction intercellular communication; GFAP—glial fibrillary acidic protein; EAAT2—excitatory amino acid transporter 2; AQP4—aquaporin 4; BDNF—brain-derived neurotrophic factor; CREB—cAMP response element-binding protein; GSK-3β—glycogen synthase kinase 3 beta; JAK2–STAT3—JAK2–STAT3 signaling pathway; TSPO—translocator protein; HMGB1—high mobility group box 1; IL-1β—interleukin -1 beta; TNF-α—tumor necrosis factor alpha; H3K9me3—histone H3 lysine 9 trimethylation; CBX—carbenoxolone; Gap26, Gap27—connexin mimetic peptide inhibitors; D4—selective connexin hemichannel inhibitor; Rg1—ginsenoside Rg1; KRG—Korean red ginseng; YAP—Yes-associated protein; miR-205, miR-221/222—microRNAs; c-Fos—neuronal activation marker; DMN—default mode network; rsFC—resting-state functional connectivity; MoCA-C—Montreal Cognitive Assessment (Chinese version); LY—Lucifer Yellow dye.

**Table 11:** Role of Connexins in Bipolar Disorder: Experimental Models, Modulation Approaches, and Key Results. Arrows indicate direction of change or relationship: ↑ increase; → indicates effect or association.

No.	Experimental Model	Subject (Animals/Cells)	BD Model Type	Connexin Isoform	Cx Modulation	Key Results	Reference
1	in vitro (primary cultures)	Rat cortical primary astrocytes	Pharmacological model (ZTP)	Cx43	ZTP (acute/subchronic/chronic administration)	Therapeutic concentrations of ZTP (chronic) ↑ release of astroglial L-glutamate through activated hemichannels and ↑ Cx43 expression in the plasma membrane; effect mediated by activation of the Akt signaling pathway; supratherapeutic doses enhance the effect	[191]
2	in vitro (iPSC-cardiomyocytes)	Cardiomyocytes derived from iPSC of BD patients	Genetic model (CACNA1C mutation associated with BD)	Cx43	CACNA1C mutation + Cx43 gene therapy	CACNA1C mutation → impairment of intercellular Cx43 gap junctions → slowing of electrical impulse conduction in the heart; restoration of Cx43 expression normalizes conduction and protects against thioridazine-induced QT prolongation	[192]

Abbreviations: BD—bipolar disorder; Cx43—connexin 43; ZTP—zotepine (atypical antipsychotic with mood-stabilizing properties); iPSC—induced pluripotent stem cells; CACNA1C—gene encoding the α1C subunit of L-type voltage-gated calcium channels; Akt—protein kinase B; GJ—gap junctions; HC—hemichannels; QT—QT interval on electrocardiogram.

**Table 12:** Role of Connexins in Schizophrenia and Suicidal Behavior: Experimental Models, Modulation Approaches, and Key Results. Arrows indicate direction of change or relationship: ↓ decrease; → indicates effect or association.

No.	Experimental Model	Subject (Animals/Cells)	Model Type	Connexin Isoform	Cx Modulation	Key Results	Reference
1	Postmortem brain analysis	Human brain (anterior cingulate cortex, N = 48 suicide victims with depression vs N = 23 controls)	Suicidal behavior in the context of depression	Cx30 (oligodendrocytes + myelinated fibers)	Observation of ↓ Cx30	↓ Cx30 in oligodendrocytes and myelinated fibers in deep layers of ACC; c expression of oligodendrocytic Cx (Cx32/Cx47) and Cx-interacting proteins → impairment of astrocyte-oligodendrocyte communication	[20]
2	Postmortem analysis + microarray/qRT-PCR	Human brain, dorsolateral prefrontal cortex (DLPFC)	Suicidal behavior	Cx30, Cx43	Observation of ↓ Cx30/Cx43 + Sox9	↓ expression of Cx30 and Cx43 in DLPFC; Sox9—transcription factor regulating Cx30	[28]

Abbreviations: ACC—anterior cingulate cortex; DLPFC—dorsolateral prefrontal cortex; Sox9—SRY-box transcription factor 9; Cx30, Cx32, Cx43, Cx47—connexins 30, 32, 43 and 47, respectively; GFAP—glial fibrillary acidic protein; qRT-PCR—quantitative real-time polymerase chain reaction; IHC—immunohistochemistry.

**Table 13:** Role of Connexins in Schizophrenia: Experimental Models, Modulation Approaches, and Key Results. Arrows indicate direction of change or relationship: ↑ increase.

No.	Experimental Model	Subject (Animals/Cells)	Model Type	Connexin Isoform	Cx Modulation	Key Results	Reference
3	Genetic study (case–control + family-based)	Schizophrenia patients (190 white + 99/163 families)	Genetic predisposition to schizophrenia	Cx50 (and Cx40)	Polymorphisms rs989192-rs4950495	Cx50 A-C haplotype significantly associated with schizophrenia; Cx40—no association	[194]
4	Genetic mutation analysis	Family with catatonic schizophrenia (15q14)	Hereditary catatonic schizophrenia	Cx36 (CX36)	Mutation screening	No causal mutations in CX36; the gene is not etiological in this pedigree	[195]
5	in vitro (primary cultures)	Rat cortical primary astrocytes	Pharmacological model (antipsychotics/mood stabilizers)	Cx43	CLZ, QTP, BPZ (± VPA)	Atypical antipsychotics (CLZ, QTP, BPZ) ↑ release of astroglial L-glutamate through activated Cx43 hemichannels (acute/subchronic); subchronically ↑ Cx43 expression in plasma membrane; effect partly via Akt	[197]

Abbreviations: Cx36, Cx40, Cx43, Cx50—connexins 36, 40, 43 and 50, respectively; CLZ—clozapine; QTP—quetiapine; BPZ—brexpiprazole; VPA—valproate; Akt—protein kinase B.

**Table 14:** Role of Connexins in Epilepsy: Experimental Models, Modulation Approaches, and Key Results. Arrows indicate direction of change or relationship: ↑ increase; ↓ decrease; → indicates effect or association.

No.	Experimental Model	Subject (Animals/Cells)	Epilepsy Model Type	Connexin Isoform	Cx Modulation	Key Results	Reference
1	in vivo (4-AP)	Adult rats (ECoG)	4-AP-induced epilepsy (neocortex)	Cx36 (neuronal GJ)	Quinine (selective Cx36 blocker) ± CBX/octanol	Cx36 blockade before induction slightly ↓ epileptogenesis; after 25–30 seizures—new pattern (>15 Hz), ↓ seizure duration and amplitude; qualitatively different effect from global GJ blockade	[14]
2	in vivo (pilocarpine)	Mice	PISE (4 h, 1 day, 1 week, 2 months)	Cx43, Cx40	Observation of ↑ Cx43/Cx40	Cx43/Cx40 ↑ in CA1/CA3 and DG at latent (1 week) and chronic (2 months) stages; localized in astrocytes; correlates with neuronal loss and epileptogenesis	[199]
3	Postmortem human analysis + experimental models	Human hippocampus (MTLE-HS) + models	MTLE-HS	Cx43, Cx30	Observation (redistribution)	No ↓ total Cx43/Cx30 expression, but pronounced perivascular redistribution of Cx43 + ↑ C-terminal phosphorylation; BBB damage (albumin) → loss of GJIC	[200]
4	in vivo (kainic acid)	TLE mice	TLE (kainate)	Cx43 (HC)	TAT-Gap19 (Cx43-HC inhibitor)	↑ Cx43 around vessels; TAT-Gap19 ↓ seizure frequency and BBB permeability	[201]
5	in vivo + ex vivo (kainate)	Mice + brain slices	Kainate-induced seizures	Cx30 (astrocytes)	Observation of ↑ Cx30	Cx30 ↑ during seizures; regulates seizure severity and glutamate clearance (independent of GJ)	[203]
6	Postmortem analysis	Human surgical samples (tumors + peritumoral cortex)	Tumor-associated epilepsy	Cx43, Cx32	Observation of ↑ Cx43/Cx32	↑ Cx43/Cx32 in glioneuronal tumors and reactive astrocytes; ↓ Cx43 in high-grade gliomas; suggested role in seizures	[204]
7	in vivo (pilocarpine)	Wistar rats	Pilocarpine model (F0, F3, F5)	Cx36, Cx43	Observation	Cx36 unchanged; Cx43 ↑ by 40% after focal seizures (F3)	[205]
8	in vivo (4-AP)	Rats	4-AP-induced epilepsy	Cx32, Cx36, Cx43	Observation of ↑	↑ Cx32/Cx36/Cx43 ipsi- and contralaterally; Cx32/Cx43 in DG and hilus; Cx36 later	[206]
9	Postmortem + surgical material	Human epileptic foci	Refractory epilepsy	Cx32, Cx43	Observation of ↑	Significantly ↑ Cx32/Cx43 in epileptic foci (immuno-EM: more colloidal gold particles)	[208]
10	in vitro + in vivo	Brain slices + WAG/Rij rats	Low Mg^2+^ (in vitro) + absence epilepsy (in vivo)	Astrocytic Cx (mainly Cx43)	Trimethylamine (GJ opening)/CBX	GJ opening ↑ SLES in focal model; CBX ↓ SLES in focal but ↑ seizures in absence epilepsy; Cx43 > Cx36	[209]
11	in vivo (pilocarpine TLE)	Rats	Pilocarpine TLE	Cx43	CBX	CBX improves CA1 microstructure, ↓ frequency/duration of SES; ↓ Cx43 and p-Cx43(Ser368)	[210]
12	in vivo (pilocarpine TLE)	Mice	Pilocarpine TLE	Cx (HC)	D4 (selective Cx-HC inhibitor)	D4 ↓ HC permeability, neuroinflammation, alters synaptic inhibition, ↑ survival, long-term suppression of glial activation	[211]
13	in vitro (HeLa)	HeLa cells transfected with Cx43/Cx30/Cx26/Panx1	—	Cx43, Cx30, Cx26, Panx1 (HC)	VPA	VPA ↑ HC activity (especially after stimulation) and ATP release via Cx43-HC	[212]
14	in vivo (lithium-pilocarpine SE)	Rats	Lithium-pilocarpine SE	Cx43	miR-23b-3p (↓ Cx43)	miR-23b-3p ↓ Cx43 → ↓ FRs, ↓ seizure severity, ↓ brain damage	[213]
15	Postmortem + surgical	Human brain (FCD, cryptogenic epilepsy)	FCD + cryptogenic epilepsy	Cx43	Observation (redistribution)	In FCD IIB—large Cx43 clusters around balloon cells; ↑ Cx43 mRNA in 25% of cryptogenic epilepsies	[214]
16	Postmortem analysis	Human hippocampus (MTLE)	MTLE	Cx43	Observation of ↑	Pronounced astrocytosis + significant ↑ Cx43 in reactive astrocytes; contributes to seizure generalization	[215]

Abbreviations: TLE—temporal lobe epilepsy; MTLE-HS—mesial temporal lobe epilepsy with hippocampal sclerosis; FCD—focal cortical dysplasia; SE—status epilepticus; SES—spontaneous epileptic seizures; PISE—pilocarpine-induced status epilepticus; 4-AP—4-aminopyridine; Cx26, Cx30, Cx32, Cx36, Cx40, Cx43, Cx50—connexins 26, 30, 32, 36, 40, 43 and 50, respectively; Panx1—pannexin 1; GJ—gap junctions; GJIC—gap junction intercellular communication; HC—hemichannels; GFAP—glial fibrillary acidic protein; CBX—carbenoxolone; TAT-Gap19—selective connexin 43 hemichannel inhibitor; D4—selective connexin hemichannel inhibitor; VPA—valproate; ZO-1—zonula occludens 1; FRs—fast ripples; miR-23b-3p—microRNA-23b-3p; ATP—adenosine triphosphate; BBB—blood–brain barrier; CA1, CA3—hippocampal Cornu Ammonis regions 1 and 3; DG—dentate gyrus; SLES—seizure-like events; ECoG—electrocorticography.

## Data Availability

No new data were created or analyzed in this study. Data sharing is not applicable to this article.

## Supplementary Materials

The following supporting information can be downloaded at: https://www.mdpi.com/article/10.3390/molecules31081341/s1. Table S1. Expanded Search Strategy for Each Database; Table S2. Characteristics and distribution of included studies by thematic categories, n = 215; Figure S1. PRISMA flow diagram illustrating the process of study identification, screening, eligibility assessment, and inclusion in the review.

## Abbreviations

The following abbreviations are used in this manuscript:

Cx	Connexins
HCs	hemichannels
Cx43	Connexin 43
p-Cx43	phosphorylated connexin 43
TBI	traumatic brain injury
SCI	spinal cord injury
PNI	peripheral nerve injury
Cx36	Connexin 36
Cx32	Connexin 32
Cx47	Connexin 47
Cx30	Connexin 30
Cx29	Connexin 29
Cx26	Connexin 26
Cx37	Connexin 37
Cx40	Connexin 40
Cx45	Connexin 45
Cx50	Connexin 50
GJs	Gap junctions
CL	intracellular loop
NTH	N-terminal helices
CSD	cortical spreading depolarization
NVU	Neurovascular unit
MCAO	middle cerebral artery occlusion
pMCAO	permanent MCAO
dMCAO	distal MCAO
OGD-R	oxygen–glucose deprivation/re-oxygenation
ICH	intracerebral hemorrhage
SAH	subarachnoid hemorrhage
NO	nitric oxide
PKC	protein kinase C
CBX	carbenoxolone
AS-ODN	antisense oligodeoxynucleotides
siRNA	small interfering RNA
ERK1/2	extracellular signal-regulated kinase 1/2
PI3K	phosphoinositide 3-kinase
BBB	blood–brain barrier
AQP4	aquaporin-4
MLKL	mixed lineage kinase domain-like protein
SVZ	subventricular zone
rtPA	recombinant tissue plasminogen activator
SNP	single nucleotide polymorphism
PNS	peripheral nervous system
CNS	central nervous system
EE	enriched environment
TNF-α	tumor necrosis factor-α
TLR4	Toll-like receptor 4
YAP	Yes-associated protein
CRTC1	CREB-regulated transcription coactivator 1
H/R	hypoxia/re-oxygenation
hUCB	human umbilical cord blood cells
TXL	Tongxinluo
BMP2/4	bone morphogenetic proteins 2/4
BMSCs	bone marrow stromal cells
NPCs	neural progenitor cells
GFAP	glial fibrillary acidic protein
ACKR3	atypical chemokine receptor 3
I/R	ischemia/reperfusion
CVS	cerebral vasospasm
BCAS	bilateral carotid artery stenosis
GAD67	glutamate decarboxylase 67
α7-nAChR	α7 nicotinic acetylcholine receptor
NOS	nitric oxide synthases
VHL	Von Hippel–Lindau
S368	serine 368
PTZ	pentylenetetrazol
ROS	reactive oxygen species
GLT-1	glutamate transporter-1
LPS	lipopolysaccharide
NAC	N-acetylcysteine
GSH	glutathione
MDA	malondialdehyde
ATP	adenosine triphosphate
OxATP	oxidized ATP
LTP	long-term potentiation
3-MA	3-methyladenine
NSCs	neural stem cells
αCT1	α-connexin carboxyl-terminal peptide
CCI	cortical impact
FPI	fluid percussion injury
GJA1-20K	20-kDa isoform of gap junction alpha-1 protein
GS-Rb1	ginsenoside Rb1
H_2_	molecular hydrogen
NSPCs	neural stem/progenitor cells
P2X7	purinergic receptor P2X7
mTOR	mechanistic target of rapamycin
SLC7A11	solute carrier family 7 member 11
GPX4	glutathione peroxidase 4
4-HNE	4-hydroxynonenal
HBO	hyperbaric oxygen therapy
HF-rTMS	high-frequency repetitive transcranial magnetic stimulation
Sig-1R	sigma-1 receptor
GlyT2	glycine transporter 2
VEGF	vascular endothelial growth factor
epSPCs	ependymal spinal cord progenitor cells
DRG	dorsal root ganglion
SGCs	satellite glial cells
BDNF	brain-derived neurotrophic factor
miR-1	microRNA-1
NLRP3	NOD-like receptor family pyrin domain-containing 3
SARM1	sterile alpha and TIR motif-containing protein 1
PSNL	partial sciatic nerve ligation
SNL	spinal nerve ligation
CFA	complete Freund’s adjuvant
CIPN	chemotherapy-induced peripheral neuropathy
COMP-Ang1	cartilage oligomeric matrix protein–angiopoietin-1
TRPA1	transient receptor potential ankyrin 1
GluK2	ionotropic kainate receptor 2
IMPs	intramembranous particles
MAM	mitochondrial-associated membrane
A2AR	adenosine A2A receptors
AD	Alzheimer’s disease
Aβ	amyloid-beta
MFN2	mitofusin-2
ApoE	Apolipoprotein E
OPCs	Oligodendrocyte progenitor cells
CD73	ecto-5′-nucleotidase
4-PBA	4-phenylbutyric acid
PD	Parkinson’s disease
SNpc	Substantia nigra pars compacta
α-syn	α-synuclein
MPTP	1-methyl-4-phenyl-1,2,3,6-tetrahydropyridine
6-OHDA	6-hydroxydopamine
GDNF	glial cell line-derived neurotrophic factor
bFGF	basic fibroblast growth factor
EGFR	epidermal growth factor receptor
LID	levodopa-induced dyskinesia
PV^+^	parvalbumin-positive
ENK^+^	enkephalin-positive
Ca^2+^	calcium ion
ALS	Amyotrophic lateral sclerosis
SOD1	superoxide dismutase 1
SOD1-G93A	glycine-to-alanine substitution at position 93 of SOD1
GJA1	Gap junction protein alpha 1
EAAT2	Excitatory amino acid transporter 2
iPSC	induced pluripotent stem cell
GAP19	Connexin 43 hemichannel inhibitory peptide
HD	Huntington’s disease
mHTT	mutant huntingtin
PolyQ	Polyglutamine expansion
CN	caudate nucleus
GP	globus pallidus
GSK-3β	glycogen synthase kinase 3 beta
JAK2	Janus kinase 2
CORT	corticosterone
STAT3	Signal transducer and activator of transcription 3
IL	interleukin
CUS	chronic unpredictable stress
PFC	prefrontal cortex
CSDS	chronic social defeat stress
BD	Bipolar disorder
ZTP	zotepine
Akt	protein kinase B
CACNA1C	calcium voltage-gated channel subunit alpha1 C
Sox9	SRY-box transcription factor 9
ACC	anterior cingulate cortex
