# Supplementary material for: The Dual Role of Connexins in Stroke, Neurotrauma, Neurodegenerative and Psychiatric Disorders: A Global Systematic Review
Source: Molecules. 2026 Apr 19;31(8):1341. doi: 10.3390/molecules31081341 (PMC13118607; doi:10.3390/molecules31081341)
Supplement: Supplementary file 1 [file molecules-31-01341-s001.zip › molecules-4231134-supplementary.pdf]

Table S1. Expanded Search Strategy for Each Database. "" — exact keyword; () — keyword grouping; "AND" — both terms required; "OR" — either term required.

Database	Search Strategy
**PubMed**	(("connexin" OR "gap junction" OR "gap junction proteins" OR "hemichannel " OR "hemichannel activity" OR "connexon" OR Cx43 OR "connexin 43" OR Cx36 OR "connexin 36" OR Cx30 OR "connexin 30" OR "connexin 26" OR "connexin 32" OR "connexin 47" OR "pannexin" OR "pannexin channels" OR "pannexin 1" OR "intercellular communication" OR "astrocytic networks" OR "astrocytic syncytium" OR "neuronal synchronization" OR "neuronal coupling") AND ("brain injury" OR "traumatic brain injury" OR "spinal cord injury" OR "peripheral nerve injury" OR "neurodegeneration" OR "neurodegenerative diseases" OR "Alzheimer disease" OR "Parkinson disease" OR "amyotrophic lateral sclerosis" OR "multiple sclerosis" OR "Huntington disease" OR "psychiatric disorders" OR "schizophrenia" OR "depression" OR "major depressive disorder" OR "bipolar disorder" OR "anxiety disorders" OR stroke OR "cerebrovascular accident" OR "ischemic stroke" OR "intracerebral hemorrhage" OR "cerebral ischemia" OR "glial cells" OR "astrocytes" OR "microglia" OR "oligodendrocytes" OR "neuroinflammation" OR "neuroinflammatory response" OR "glial activation" OR "glial reactivity" OR "cytokine release" OR "blood-brain barrier disruption" OR OR "blood-brain barrier" OR "neuronal death" OR "apoptosis" OR "oxidative stress" OR "mitochondrial dysfunction" OR "epilepsy" OR "epileptic seizures" OR "epileptogenesis"))
**Scopus**	TITLE-ABS-KEY ("connexin" OR "gap junction" OR "hemichannel" OR "connexon" OR "connexin 43" OR "connexin 36" OR "connexin 30" OR "connexin 26" OR "connexin 32" OR "connexin 47" OR "pannexin" OR "pannexin 1" OR "intercellular communication" OR "astrocytic networks" OR "astrocytic syncytium" OR "neuronal synchronization" OR "neuronal coupling") AND TITLE-ABS-KEY ("brain injury" OR "traumatic brain injury" OR "spinal cord injury" OR "peripheral nerve injury" OR "neurodegeneration" OR "neurodegenerative diseases" OR "Alzheimer disease" OR "Parkinson disease" OR "amyotrophic lateral sclerosis" OR "multiple sclerosis" OR "Huntington disease" OR "psychiatric disorders" OR "schizophrenia" OR "depression" OR "major depressive disorder" OR "bipolar disorder" OR "anxiety disorders" OR "stroke" OR "cerebrovascular accident" OR "ischemic stroke" OR "intracerebral hemorrhage" OR "cerebral ischemia" OR "glial cells" OR "astrocytes" OR "microglia" OR "oligodendrocytes" OR "neuroinflammation" OR "neuroinflammatory response" OR "glial activation" OR "glial reactivity" OR "cytokine release" OR "blood-brain barrier disruption" OR "blood-brain barrier" OR "neuronal death" OR "apoptosis" OR "oxidative stress" OR "oxidative damage" OR "mitochondrial dysfunction" OR "epilepsy" OR "epileptic seizures" OR "epileptogenesis")
**Web of Science**	TOPIC: ("connexin" OR "gap junction" OR "hemichannel" OR "connexon" OR "connexin 43" OR "connexin 36" OR "connexin 30" OR "connexin 26" OR "connexin 32" OR Cx47 OR "connexin 47" OR "pannexin 1" OR "intercellular communication" OR "astrocytic networks" OR "astrocytic syncytium" OR "neuronal synchronization" OR "neuronal coupling") AND TOPIC: ("brain injury"

	OR "traumatic brain injury" OR TBI OR "spinal cord injury" OR SCI OR "peripheral nerve injury" OR neurodegeneration OR "neurodegenerative diseases" OR "Alzheimer disease" OR "Parkinson disease" OR "amyotrophic lateral sclerosis" OR "multiple sclerosis" OR "Huntington disease" OR "psychiatric disorders" OR "schizophrenia" OR "depression" OR "major depressive disorder" OR "bipolar disorder" OR "anxiety disorders" OR stroke OR "cerebrovascular accident" OR "ischemic stroke" OR "intracerebral hemorrhage" OR "cerebral ischemia" OR "glial cells" OR "astrocytes" OR "microglia" OR "oligodendrocytes" OR "neuroinflammation" OR "neuroinflammatory response" OR "glial activation" OR "glial reactivity" OR "cytokine release" OR "blood-brain barrier disruption" OR "blood-brain barrier" OR "neuronal death" OR "apoptosis" OR "oxidative stress" OR "oxidative damage" OR "mitochondrial dysfunction" OR "epilepsy" OR "epileptic seizures" OR "epileptogenesis")

Table S2. Characteristics and distribution of included studies by thematic categories, n=215.

Section	Category	Number	List
Introduction	Articles	6	1, 29, 30, 31, 32, 33
Materials and Methods	Articles	1	34
Role of Connexins in Stroke	Experimental	42	3, 4, 6, 7, 8, 9, 13, 16, 17,37, 38, 39, 40, 41, 42, 43, 44, 45, 46, 47, 48, 49, 50, 51, 52, 53, 54, 55, 56, 57, 58, 59, 60, 61, 62, 63, 64, 65, 66, 67, 68, 69
	Reviews	3	2, 35,36
Traumatic Brain Injury	Experimental	22	5, 71, 72, 73, 74, 75, 76, 77, 78, 79, 80, 81, 82, 83, 84, 85, 86, 87, 88, 89, 90, 91
	Reviews	1	70
Spinal Cord Injury	Experimental	17	10, 11, 12, 94, 95, 96, 97, 98, 99, 100, 101, 102, 103, 104, 105, 106, 107
	Reviews	2	92, 93
Peripheral Nerve Injury	Experimental	21	15, 109, 110, 111, 112, 113, 114, 115, 116, 117, 118, 119, 120, 121, 122, 123, 125, 126, 127, 128, 129
	Reviews	2	108,124
Alzheimer's Disease	Experimental	13	18, 21,131,132,133, 134, 135, 136, 137, 138, 139, 140, 141

	Reviews	1	130
Parkinson's Disease	Experimental	13	22, 143, 144, 145, 146, 147, 148, 149, 150, 151, 152, 153, 154
	Reviews	1	142
Amyotrophic Lateral Sclerosis	Experimental	9	19, 23, 155, 156, 157, 158, 159, 160, 161
Huntington's Disease	Experimental	3	24, 162, 163
Depression	Experimental	26	26,165, 166, 167, 168, 169, 170, 171,172, 173, 174, 175, 176, 177, 178, 179, 180, 181, 182, 183, 184, 185, 186, 187, 188, 189
	Reviews	2	25, 164
Bipolar Disorder	Experimental	2	191, 192
	Reviews	2	27, 190
Suicidal Behavior	Experimental	2	20, 28
Schizophrenia	Experimental	3	194, 195, 197
	Reviews	2	193, 196
Epilepsy	Experimental	17	14, 199, 200, 201, 203, 204, 205, 206, 207, 208, 209, 210, 211, 212, 213, 214, 215
	Reviews	2	198, 202

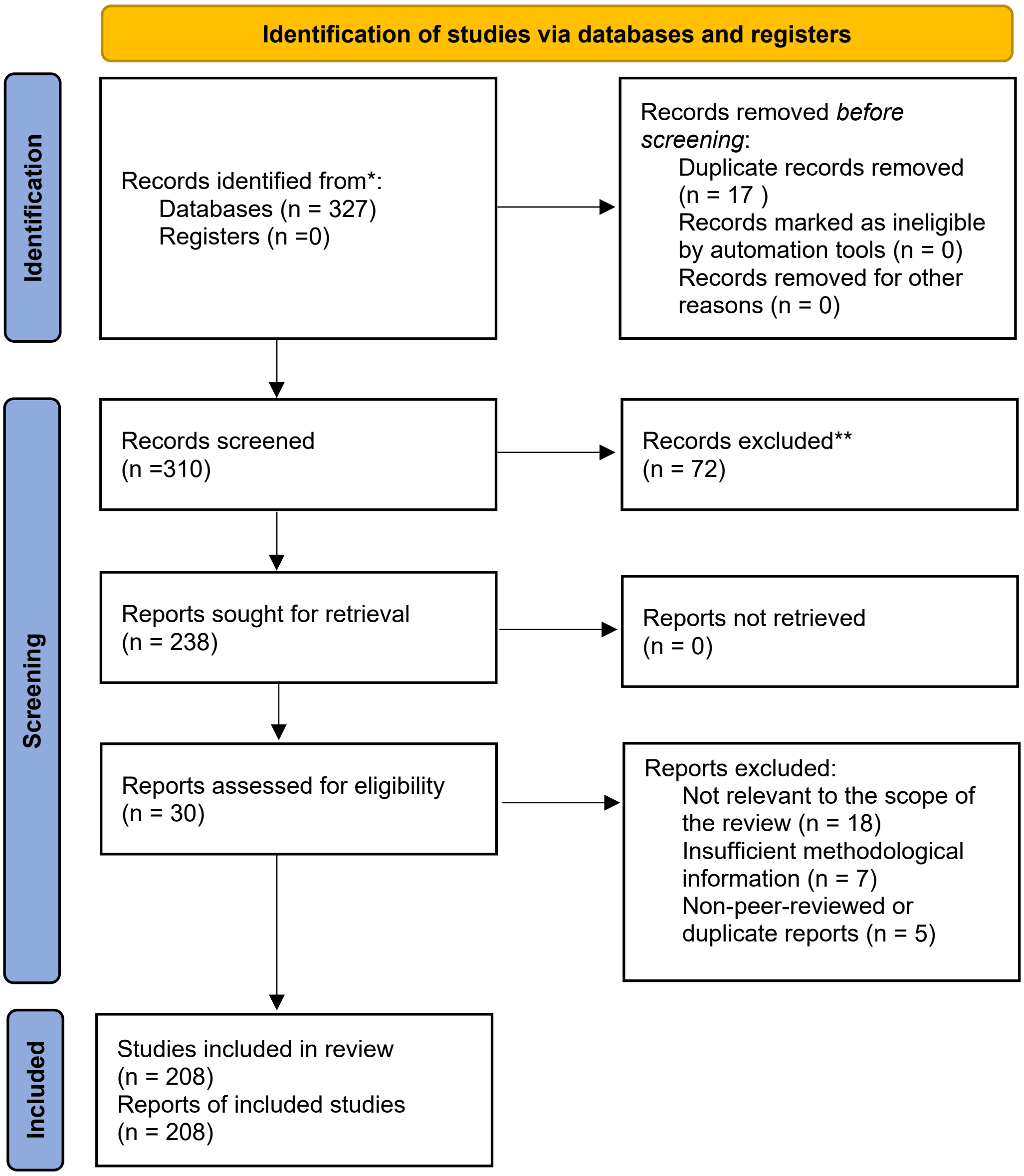

Figure S1. PRISMA flow diagram illustrating the process of study identification, screening, eligibility assessment, and inclusion in the review. An expanded PRISMA-ScR flowchart is provided in the Supplementary Materials.
